# Tight gene co-expression in BCB positive cattle oocytes and their surrounding cumulus cells

**DOI:** 10.1186/s12958-022-00994-3

**Published:** 2022-08-13

**Authors:** Bailey N. Walker, Jada Nix, Chace Wilson, Mackenzie A. Marrella, Savannah L. Speckhart, Lydia Wooldridge, Con-Ning Yen, Jocelyn S. Bodmer, Laila T. Kirkpatrick, Sarah E. Moorey, David E. Gerrard, Alan D. Ealy, Fernando H. Biase

**Affiliations:** 1 School of Animal Sciences, Virginia Polytechnic Institute and State University, 175 W Campus Dr, Blacksburg, VA 24061 USA; 2 Department of Animal Science, University of Tennessee, Knoxville, TN USA

**Keywords:** Oocyte, Developmental competence, Transcriptome, Single cell, Embryos

## Abstract

**Background:**

Cytoplasmic and nuclear maturation of oocytes, as well as interaction with the surrounding cumulus cells, are important features relevant to the acquisition of developmental competence.

**Methods:**

Here, we utilized Brilliant cresyl blue (BCB) to distinguish cattle oocytes with low activity of the enzyme Glucose-6-Phosphate Dehydrogenase, and thus separated fully grown (BCB positive) oocytes from those in the growing phase (BCB negative). We then analyzed the developmental potential of these oocytes, mitochondrial DNA (mtDNA) copy number in single oocytes, and investigated the transcriptome of single oocytes and their surrounding cumulus cells of BCB positive versus BCB negative oocytes.

**Results:**

The BCB positive oocytes were twice as likely to produce a blastocyst in vitro compared to BCB- oocytes (*P* < 0.01). We determined that BCB negative oocytes have 1.3-fold more mtDNA copies than BCB positive oocytes (*P* = 0.004). There was no differential transcript abundance of genes expressed in oocytes, however, 172 genes were identified in cumulus cells with differential transcript abundance (FDR < 0.05) based on the BCB staining of their oocyte. Co-expression analysis between oocytes and their surrounding cumulus cells revealed a subset of genes whose co-expression in BCB positive oocytes (*n* = 75) and their surrounding cumulus cells (*n* = 108) compose a unique profile of the cumulus-oocyte complex.

**Conclusions:**

If oocytes transition from BCB negative to BCB positive, there is a greater likelihood of producing a blastocyst, and a reduction of mtDNA copies, but there is no systematic variation of transcript abundance. Cumulus cells present changes in transcript abundance, which reflects in a dynamic co-expression between the oocyte and cumulus cells.

**Supplementary Information:**

The online version contains supplementary material available at 10.1186/s12958-022-00994-3.

## Background

Folliculogenesis is a dynamic process that involves the synchronic action of multiple cell types and many signaling pathways operating at specific spatial and temporal dimensions [1]. Following the activation of the primordial follicle, the oocyte will become transcriptionally active, accumulating ribonucleic acids (RNA) and proteins from thousands of genes [2, 3], rearrangement of organelles, and chromatin condensation [4, 5]. The somatic cells (granulosa) surrounding the oocyte will proliferate and differentiate [6] to form the cumulus-oocyte complex. The processes involving oocyte cytoplasmic maturation and the differentiation of cumulus cells (CC) are essential for the acquisition of developmental competence by the oocyte [7, 8], successful fertilization, and embryonic development [9].

Interestingly, it is possible to identify oocytes that have completed growth and have undergone most of their cytoplasmic maturation with a supravital dye of blue coloration, brilliant cresyl blue (BCB) [10]. Cumulus-oocyte complexes can be exposed to BCB for oocyte staining and further used for standard in vitro embryo production. Studies performed in mice have indicated that fully-grown oocytes have a significant reduction of Glucose-6-Phosphate Dehydrogenase (G6PDH) enzyme activity relative to growing oocytes [11]. When active, in immature oocytes, the enzyme G6PDH participates in the pentose phosphate pathway which produces the co-enzyme nicotinamide adenine dinucleotide phosphate (NADP^+^) and a hydrogen ion (H^+^). The BCB dye captures electrons from the electron transport chain and becomes colorless [10]. Indeed, bovine oocytes that show loss of BCB coloration have greater G6PDH activity compared to those oocytes that remain blue following BCB staining [12]. Thus, BCB staining serves as a visual marker for cytoplasmic maturation in oocytes.

The categorization of oocytes by BCB staining has been associated with many molecular and physical properties in mice, pigs, sheep, and cattle. For instance, BCB positive oocytes (with low G6PDH activity) have a greater diameter [13, 14] and lipid content [15] as well as more centrally located mitochondria [16] and cortical granules distributed towards the periphery [16, 17] relative to BCB negative oocytes. The BCB positive and negative oocytes also differ in transcriptome profiles [18–20] and mitochondria abundance [15, 19, 21, 22]; however, the findings are not consistent across reports. Most notably, BCB positive oocytes have a greater yield of blastocysts produced in vitro relative to their BCB negative counterparts [12, 16, 19, 23–25].

Significant gaps remain in our understanding of the acquisition of developmental competence by oocytes. Furthermore, knowledge about the interaction between oocytes and cumulus cells and the mechanisms that lead to a coordinated regulation of gene transcription in both compartments of the cumulus-oocyte complex during folliculogenesis is strikingly limited. Here, we tested three complementary hypotheses related to the distinction of cattle oocytes based on their growth phase using BCB staining as a proxy: (i) BCB positive oocytes have greater developmental competence relative to BCB negative oocytes; (ii) BCB positive oocytes have a greater number of mitochondrial DNA (mtDNA) relative to the BCB negative oocytes; and (iii) BCB positive and negative oocytes, alongside their surrounding cumulus cells, have differing transcriptome profiles that relate to the oocyte stage of maturation. Collectively, our findings reveal increased regulation of gene transcription in oocytes and cumulus cells if oocytes complete their growth and achieve higher developmental competence relative to immature oocytes.

## Methods

Raw data were deposited in the Gene Expression Omnibus repository [26], accession GSE199210. The codes used for all analytical procedures were deposited in the figshare repository [27], which can be accessed at https://biase-lab.github.io/oocyte_cumulus_BCB/index.html, and are presented as Supplementary code. Reagents were purchased from Sigma-Aldrich (Milwaukee, WI) unless otherwise specified. All samples obtained for this experiment were obtained post-mortem and there was no handling of live animals for this experiment.

### Overview of the experimental design

The focal point of the paper was the separation of cumulus-oocyte complexes into BCB positive and BCB negative, which indicates whether the oocyte has completed its growth (BCB positive) or not (BCB negative). In experiment 1, we tested whether the oocytes classified as BCB positive would have greater developmental competence relative to the oocytes classified as BCB negative. In experiment 2, we compared the abundance of mtDNA copies between oocytes that were classified as BCB positive and BCB negative. In experiment 3, we classified the cumulus-oocyte complexes based on the oocyte staining with BCB, and produced RNA-sequencing data from the oocytes and the corresponding cumulus. First, we compared the transcriptome within a compartment (oocytes and cumulus cells separately), based on the oocyte staining. Next, we estimated co-expression between genes expressed in oocytes and the corresponding cumulus cells and compared the networks based on their BCB classification.

#### Experiment 1: assessment of blastocyst yield in oocytes classified by BCB staining

##### Collection of cumulus-oocyte complexes

Cattle (*Bos taurus*) ovaries were obtained from an abattoir and transported to the laboratory in a saline solution (0.9% NaCl) with antibiotic and antimycotic (1x). We then aspirated cumulus-oocyte complexes from antral follicles ranging from 3 to 8 mm in diameter with an 18-gauge needle attached to a 10 ml syringe into oocyte collection media (TCM-199 with Hank’s salts supplemented with 10% fetal bovine serum (FBS), 104.7 mM gentamicin, and 250 mM pyruvate). We only kept oocytes with a homogeneous cytoplasm and three or more compact layers of cumulus cells.

##### ***BCB Staining***

The BCB staining procedure followed previously described procedures [12, 16, 19, 23], with modifications. Immediately after collecting cumulus-oocyte complexes from follicles, the cumulus-oocyte complexes were washed in phosphate-buffered saline solution containing 0.2% [w/v] bovine serum albumin (BSA) fraction V. Next, we incubated cumulus-oocyte complexes in a solution containing BCB (Sigma) at 26 µM diluted in PBS and 0.2% BSA for 15 min at 38.5 °C in 5% CO_2_, humidified atmosphere. After incubation, the cumulus-oocyte complexes were washed three times in PBS 0.2% BSA. The classification was carried out by two researchers with the aid of a stereoscope. The cumulus-oocyte complexes that had no blue coloration in the ooplasm were classified as BCB negative, whereas cumulus-oocyte complexes that had a blue coloration in the ooplasm were classified as BCB positive.

##### In Vitro*** Maturation (IVM)***

All procedures were based on a standard protocol for in vitro production of embryos [28]. We washed the cumulus-oocyte complexes one time in oocyte maturation medium (TCM-199 + Earle’s Salts supplemented with 25 µg/ml follicle-stimulating hormone, 2 µg/ml estradiol, 46 µM gentamycin, 1 mM glutamax, 250 mM sodium pyruvate, and 10% FBS). Next, we placed the cumulus-oocyte complexes with the same BCB classification in groups of ten into 50 µl droplets of maturation medium under mineral oil followed by incubation at 38.5 °C under 5% CO2, 95% air atmosphere, for approximately 22 h.

##### In Vitro* Fertilization (IVF)*

Following IVM, we washed cumulus-oocyte complexes three times in HEPES (4-(2-hydroxyethyl)-1-piperazineethanesulfonic acid)-buffered synthetic oviductal fluid (HEPES-SOF [28, 29], containing 3 mg/ml fraction V BSA, 0.2 mM sodium pyruvate, 7.5 µg/ml gentamicin, 5.3 mM sodium-lactate, 107.7 mM sodium chloride, 10 mM HEPES, 2 mM sodium bicarbonate, 1.17 mM calcium chloride dihydrate, 1.19 mM potassium phosphate monobasic, 7.16 mM potassium chloride, and 0.49 mM magnesium chloride hexahydrate), followed by two washes in fertilization medium, and placed in a final fertilization medium plate (SOF-Fert [28, 29], containing 6 mg/ml essentially fatty acid free BSA, 1 mM sodium pyruvate, 10.5 mM gentamicin, 0.01 mg/ml heparin, 1 mM caffeine, 5.3 mM sodium-lactate, 107.7 mM sodium chloride, 25.07 mM sodium bicarbonate, 1.17 mM calcium chloride dihydrate, 1.19 mM potassium phosphate monobasic, 7.16 mM potassium chloride, and 0.49 mM magnesium chloride hexahydrate).

For fertilization, we used straws containing heterospermic bovine frozen semen (three bulls), prepared with the density gradient isolation procedure according to a protocol previously described [28] using BoviPure and BoviDilute (Nidacon International, Molndal, Sweden). We added sperm to the solution containing cumulus-oocyte complexes at a final concentration of 1,000,000 sperm/ml. Oocytes and sperm were co-cultured for 16 to 18 h and incubated under the same conditions as described for IVM.

##### In Vitro*** Culture (IVC)***

We removed the cumulus cells from presumptive zygotes by gentle pipetting, followed by three washes in HEPES-SOF and two washes in SOF culture media (SOF-BE1 [28, 29], containing 4 mg/ml essentially fatty acid free BSA, 5.3 mM sodium-lactate, 107.7 mM sodium chloride, 25.07 mM sodium bicarbonate, 1.17 mM calcium chloride dihydrate, 1.19 mM potassium phosphate monobasic, 7.16 mM potassium chloride, and 0.49 mM magnesium chloride hexahydrate, 1 mM glutamax, 0.4 mM sodium pyruvate, 1 × MEM-non-essential amino acid solution, 1 × BME-essential amino acid solution, 52.3 mM gentamicin, 0.5 mM sodium citrate, and 2.77 mM myo-inositol). Then, groups of 25–30 putative zygotes were placed in 50 µl SOF-BE1 drops under mineral oil and cultured for 8 days at 38.5 °C in a humidified tri-gas (5% CO_2_, 5% O_2_, 90% N_2_) [28] incubator. Blastocyst yield was recorded on day 7 post-fertilization. All blastocysts, from early to hatched, were included in the statistical analysis.

##### Statistical analysis of blastocyst yield based on oocyte BCB staining

For each replicate, the number of embryos that developed to blastocyst stage and the number of putative zygotes with arrested development prior to blastocyst formation were recorded. Blastocyst yield (percentage) was then calculated as the proportion of blastocysts developed relative to the total number of putative zygotes cultured. We analyzed count data (success of blastocyst development or developmental arrest) using a general linear model with a binomial family, which results in logistic regression analysis [30]. We used the number of blastocysts and the number of putative zygotes that failed to develop into blastocysts as the dependent variable, and added oocyte groups (BCB positive, BCB negative, controls) and the replicate (four replicates) to the model as fixed-effects. We assessed the effect of staining category on blastocyst proportion with the Wald’s test of probability ratio [31] and the Likelihood ratio test [32]. Hypothesis tests on linear contrasts (BCB positive and BCB negative, BCB positive and control, and BCB negative and control) were further assessed with odds ratio [33] and multiple comparisons of probabilities. Statistical significance in proportions of blastocyst was assessed at alpha = 0.05.

#### Experiment 2: quantification of mtDNA copy number in oocytes separated by BCB Staining

##### Extraction and linearization of mtDNA from single oocytes

We exposed mitochondrial DNA (mtDNA) from single oocyte samples by using 4 µl of Lucigen QuickExtract DNA Extraction Solution (Lucigen, Middleton, WI) per oocyte and incubating the solution at 65ºC for 15 min followed by 98 °C for 2 min in a thermocycler.

Next, we used the SWA1 endonuclease (New England BioLabs, Ipswich, MA) to linearize the circular mtDNA through the cleaving of one target site (ATTT/AAAT). Linearization was achieved by the addition of a mix containing 1 µl of SWA1 endonuclease (10 IU), and 0.6 µl of NEbuffer r3.1 (New England BioLabs, Ipswich, MA) to each oocyte lysate. We then incubated this solution at 25 °C for ~ 16 h in a thermocycler followed by inactivation at 65ºC for 20 min. Samples were stored at -20 °C until used.

##### Preparation of a standard curve

We used purified bovine mtDNA in our standard curve. To purify mitochondria, we obtained postmortem samples of the masseter muscle (*Bos taurus*), cut into pieces weighing 1.5 g and placed in a 50 ml centrifuge tube containing 10 ml of homogenization buffer (100 mM sucrose, 180 mM potassium chloride, 50 mM tris-base, 5 mM magnesium dichloride, 1 mM ATP sensitive potassium, and 10 mM of ethylenediaminetetraacetic acid, in a solution at a pH of 7.4).

We chopped the samples to fine pieces and added 4 µg of protease from *Bacillus lincheniformis* (MilliporeSigma, Burlington, MA) followed by incubation for 10 min on ice. We then homogenized samples and suspended them to a final volume of 35 ml with homogenization buffer. We then filtered the solution through two layers of cheesecloth into a beaker on ice and transferred the filtrate into a 50 ml centrifuge tube. We centrifuged the samples at 138 × g at 4 °C for 10 min. We filtered the supernatant using two layers of cheesecloth and transferred it into new 50 ml centrifuge tubes. Then we centrifuged the samples at 8820 × g, at 4 °C for 10 min, discarded the supernatant, and the pellet containing the purified mitochondria was washed with 200 µl of mannitol sucrose medium (220 mM mannitol, 70 mM sucrose, 10 mM tris–HCL, and 100 mM egtazic acid (EGTA), in solution at a pH 7.4). We stored purified mitochondria in microcentrifuge tubes at -20 °C until further use. We isolated mtDNA from 200 µl mitochondria extract with a Zymo Quick-DNA Miniprep Plus Kit (Zymo Research, Irving, CA) using the ‘Biological Fluids and Cells’ workflow protocol.

We linearized mtDNA in a solution of 10.5 µl water (MilliporeSigma, Burlington, MA), 2.5 µl SWA1 endonuclease (25 IU), and 2.5 µl NEbuffer r3.1 (1x) and 55.3 ng of purified *Bos taurus* mtDNA in a final reaction volume of 20 µl. Samples were incubated at 25 °C for ~ 16 h and enzyme inactivation was achieved by incubation at 65 °C for 20 min. Samples were stored at -20 °C.

We prepared a standard curve with linearized mtDNA. First, we quantified the mtDNA on a Qubit 4 Fluorometer (Invitrogen™, Thermo Fisher Scientific, Waltham, MA) using the dsDNA HS assay kit (Invitrogen™, Thermo Fisher Scientific, Waltham, MA). Then, we produced twofold serial dilutions, starting with 0.436 ng/µl of purified and linearized mtDNA. We prepared a standard curve with eight points, and we confirmed the concentration by quantifying the first four dilutions on a Qubit 4 Fluorometer and a dsDNA HS assay kit. Each reaction contained 6 µl of the template.

##### Real-time quantitative polymerase chain reaction

We obtained the sequences for primer (F: 5'-CCTACAAACGCTCCTTCCACT-3', R: 5'-AGAGAATATAGGGCGGTGATTACT-3') and custom Taqman probe (FAM-TTGTTGGGGGTAGAGCTAAGTTGGT-MGBNFQ) sequences from reference [34].

A total of 53 BCB positive and 40 BCB negative oocytes were used for this experiment. The linearized mtDNA from each oocyte (6 µl) was mixed with the PCR reaction mix (1 × Taqman Fast Advanced Master Mix (Thermo Fisher Scientific, Waltham, MA), forward and reverse mtDNA primers at 0.01 mM, and 0.5 µl of the Taqman probe (Thermo Fisher Scientific, Waltham, MA)) in a final volume of 100 µl. This solution containing the lysate from a single oocyte was mixed thoroughly and split into two 50 µl reactions. The reactions were carried out on MicroAmp™ Optical 96-Well Reaction Plates (Thermo Fisher Scientific, Waltham, MA) using an Applied Biosystems 7500 Fast PCR system (Thermo Fisher Scientific, Waltham, MA). The cycling conditions were as follows: 50 °C for 2 min, 95 °C for 2 min, followed by 40 cycles of 95 °C for 15 s and 60 °C for 30 s. All crossing points were assigned using the default parameters and the absolute quantification of copy number was obtained automatically from the 7500 Software (v2.0.6).

##### Statistical analysis of mtDNA copy number relative to oocyte BCB staining

Due to assaying reactions in three plates, we analyzed the data using Analysis of Variance [35] (type III) in R software [36]. The model contained the fixed effects of plate (1, 2 or 3) and oocyte group (BCB positive or BCB negative). Next, we assessed the significance of the difference between the least-square means using the Tukey Honestly Significant Difference test [37]. Statistical significance in averages of copy number of mtDNA between groups of oocytes was assessed at alpha = 0.05.

#### Experiment 3: differential transcriptome analysis of COCs separated by BCB Staining

##### Separation of oocytes from cumulus cells

We separated cumulus cells from oocytes based on a previously described procedure [38]. We placed each individual cumulus-oocyte complex in 2 µl drops of Trypsin (TrypLE Express, Grand Island, NY). Oocytes were mechanically separated from their surrounding cumulus cells with gentle pipetting. Then, we collected cumulus cells (~ 3 µl volume) and placed in a microcentrifuge tube (200 µl) in liquid nitrogen. We washed individual oocytes three times in 2 µl drops of PBS containing 0.2% BSA fraction V and collected oocytes, in a minimal volume, in individual microcentrifuge tubes (200 µl) and froze them in liquid nitrogen. All frozen cells were stored at -80 °C until RNA extraction.

##### RNA extraction and library preparation

All extractions and analytical procedures were performed on individual oocytes and cumulus cells from one cumulus-oocyte complex following the protocol described elsewhere [39]. We extracted total RNA with TRIzol™ Reagent with the aid of Phasemaker Tubes for improved yield and purity of the RNA [39–42]. Total RNA was stored in 70% ethanol at -80 °C [39].

Prior to proceeding with library preparation, we assessed the RNA integrity resulting from our extraction in a 2100 Bioanalyzer using the Agilent RNA 6000 Pico Kit (Agilent Technologies, Germany). This assessment required the totality of the oocyte sample to be utilized, thus, this was only performed on test samples to assess the quality and rigor of our procedures.

For preparation of libraries to undergo efficient single-cell RNA sequencing, the following procedures were modified from the mcSCRB-seq protocol [39, 43]. Seventy percent ethanol was removed from the RNA pellets and dried out for few minutes. Next, the RNA pellet was resuspended in a solution containing an oligo-dTVN primer (5’-AAGCAGTGGTATCAACGCAGAGTACT_30_VN-3’). Tubes were heated to 72 °C for three minutes to denature secondary RNA structures. Then, 5 µl of RT mix was added, containing 200 U/µl Maxima H Minus Reverse Transcriptase, 1 × Maxima RT Buffer, 7.5% PEG 8000, 10 mM dNTPs, and 2 µM of a template-switching oligo (5’-AAGCAGTGGTATCAACGCAGAGTACATrGrG + G-3’) to the reaction. Tubes were incubated at 42 °C for 1.5 h to generate full-length cDNA. Next, we purified the cDNA product with AMPure XP beads to remove reagents prior to amplification.

Immediately following the purification, a preamplification mix was added containing 1.25 U Terra polymerase, 1 × Terra direct buffer, and 0.1 µM of primer (5’-AAGCAGTGGTATCAACGCAGAGT-3’), to each tube. Amplification was performed with 8 PCR cycles under the following conditions: 98 °C for 15 min, 68 °C for 5 min, and 72 °C for 10 min. After the final cycle, the reaction was held at 8 °C. Finally, we purified products with AMPure XP beads. Then, we assessed quality of the amplification the 2100 Bioanalyzer and the Agilent High Sensitivity DNA kit. We quantified the products with a Qubit 4 fluorometer.

We used one ng of the amplified cDNA as a template for library preparation using the Nextera DNA Flex Library Prep kit following the manufacturer’s protocol. We amplified the tagmented cDNA with 13 cycles of PCR. Following a purification of the library with AMPure XP beads, we assessed the DNA profile with a 2100 Bioanalyzer, using the Agilent High Sensitivity DNA kit and quantified with a Qubit 4 fluorometer. Libraries were sent to VANTAGE, Vanderbilt Technologies for Advanced Genomics at Vanderbilt University, to produce paired-end sequencing with reads 150 nucleotides long using an Illumina HiSeq 2500 platform.

##### Processing of raw data and quantification of transcript abundance

We processed and analyzed data from 38 samples: 19 individual oocytes and 19 groups of surrounding cumulus cells. These samples were classified based on BCB staining, resulting in nine pairs of cumulus cells and oocytes that were BCB positive and ten pairs of cumulus cells and oocytes that were BCB negative. Paired-end reads were trimmed at the 5’ and 3’ ends, then aligned to the ARS-UCD1.2 assembly of the *Bos taurus* genome with *HiSat2* [44]. Following that, unmapped reads and those with low quality scores were filtered out with *Samtools* [45]. Aligned reads were sorted and indexed using Picard [46]. Aligned reads were quantified against the Ensembl gene annotation (*Bos taurus* ARS-UCD1.2.98), with duplicates removed, using *featurecounts* [47]. Following quantification, further analysis was performed on R software [36]. In oocytes and cumulus cells, lowly expressed genes with less than two counts per million (cpm) or one transcript per million (TPM) in eight or fewer samples were filtered out.

##### Statistical analyses of RNA-seq data

All statistical analyses were performed in R software [36]. We carried out differential gene expression analysis contrasting BCB positive vs. BCB negative oocytes and BCB positive vs. BCB negative cumulus cells using the packages edgeR [48] and DeSeq2 [49]. Differential transcript abundance was inferred as significant if false discovery rate (FDR) < 0.01 for both algorithms.

Co-expression analysis is the quantification of a correlation metric between the transcript abundance of two genes [50]. For co-expression analysis between genes expressed in oocytes and cumulus cells, we normalized the raw counts using the trimmed mean of M values (TMM) approach [51], followed by an arcsine transformation log(x + √(× 2 + 1)) [52]. This normalization and transformation produce robust co-expression networks [53]. Then, we calculated the Pearson’s correlation [54] between the genes expressed in pair of oocytes and corresponding cumulus cells using the WGCNA package [55]. We estimated the empirical False Discovery Rate (eFDR) using the approach and formula described elsewhere [56, 57].

## Results

### Blastocyst yield from COCs separated based on BCB staining

**Fig. 1:**
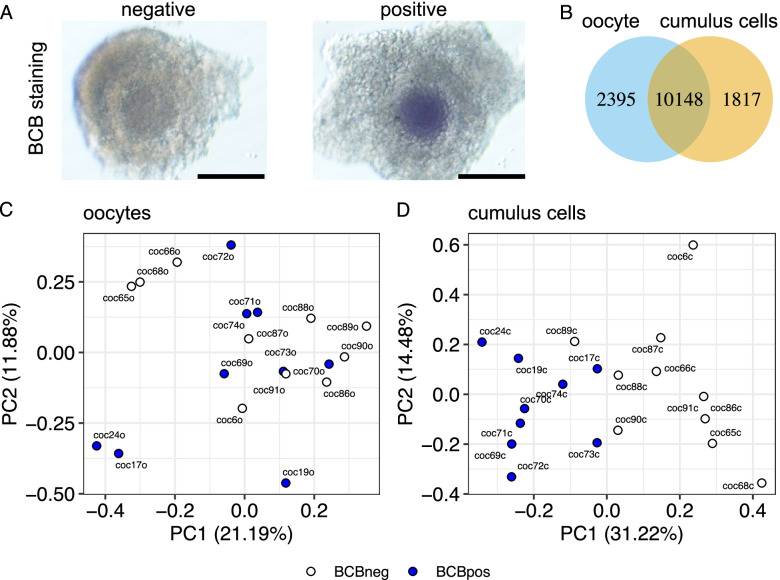
Transcriptome analysis of cumulus-oocyte complexes. **A** Schematics of sample classification based on BCB staining. **B** Number of protein-coding or long noncoding genes with transcripts quantified in single oocytes and corresponding cumulus cells. **C** Principal component analysis of oocytes. **D** Principal component analysis of cumulus cells. For both (**C** and **D**), empty circles indicate BCB negative, and blue circles indicate BCB positive

Following the aspiration of follicles from bovine ovaries for the collection of cumulus-oocyte complexes, we selected 697 COCs based on their morphology (homogeneous oocyte cytoplasm and three or more compact layers of cumulus cells) for staining with BCB dye. Next, we classified the cumulus-oocyte complex based on whether oocytes retained (BCB positive) or lost (BCB negative) the blue coloration (Fig. 1A). Forty-three and 57% of the cumulus-oocyte complexes were classified as BCB positive (*N* = 302) or BCB negative (*N* = 395), respectively. We used 121 non-stained COCs as controls.

We proceeded with in vitro oocyte maturation and in vitro production of embryos with the groups of BCB classified cumulus-oocyte complexes and control cumulus-oocyte complexes (not exposed to the dye). Our analysis showed a significant effect of selecting cumulus-oocyte complexes for BCB staining on blastocyst yield (*P* = 0.0015, Wald’s test [32], *P* = 0.0012, Likelihood ratio test [32], Table 1). Putative zygotes produced from BCB positive oocytes yielded 18% (± 2.4 SE) of blastocysts on day seven of in vitro culture (~ 190 h post fertilization), compared to 9% (± 1.5 SE) of blastocysts produced from zygotes originated from BCB negative cumulus-oocyte complexes (*P* = 0.001, Table 2). Neither group was significantly different from the blastocyst yield obtained with the control zygotes (14% ± 3.5 SE) that originated from cumulus-oocyte complexes not exposed to BCB (Table 2).

**Table 1:** Summary of the Wald’s and Likelihood ratio tests of proportion following the logistic regression analysis for blastocysts on day seven of culture

Variable	Wald’s test			Likelihood ratio test	
	DF	${\upchi }^{2}$	P	${\upchi }^{2}$	P
Group	2	12.96	< 0.01	13.39	< 0.01
Replicate	3	6.24	0.1	-	-

*DF* Degrees of freedom, ${\chi }^{2}$ Chi-square, *P* Probability

**Table 2:** Summary of the hypothesis tests for linear contrasts between COC groups

	Multiple comparison of means, Tukey			Odds ratio		
Contrast	Estimate	SE	P	Odds ratio	SE	P
BCB + vs BCB-	0.84	0.23	< 0.01	2.1	0.1	< 0.01
BCB + vs Control	0.29	0.33	0.66	1.2	0.35	0.66
BCB- vs Control	-0.55	0.35	0.24	0.61	0.18	0.24

*SE* Standard error, *P* Probability

### Mitochondrial DNA abundance in single oocytes classified by BCB staining

The absolute quantification of mtDNA copies in single oocytes showed an overall average of 1,681,647 ± 827,192. An analysis of variance revealed a significant effect of oocyte groups (BCB positive, BCB negative, *P* = 0.0044, Supplementary Table S1). A Tukey’s test identified that the mean value of mtDNA copy number was significantly different between BCB positive (1,475,377 ± 762,595 SE) and BCB negative (1,908,041 ± 844,937 SE) oocytes (*P* = 0.0044, Supplementary Table S2, Supplementary figure S1).

### Transcriptome profiling of single oocytes and the corresponding cumulus cells

We sequenced the transcriptome of 19 cumulus-oocyte complexes, of which nine contained BCB positive oocytes and ten contained BCB negative oocytes, respectively. For genome-wide analysis of gene transcript abundance, we separated the cumulus cells from the oocytes ([57] see material and methods for details). Overall, we produced an average of 39,426,362 and 43,536,143 pairs of reads for single oocytes and cumulus cells, respectively. After filtering for lowly expressed genes, we quantified the transcript abundance for 12,543 and 11,965 protein coding or long noncoding genes in oocytes and cumulus cells, respectively (Fig. 1B). A principal component analysis with all genes quantified indicated no clear trend of separation of the oocytes based on the BCB staining (Fig. 1C); however, a pattern of separation of cumulus cells emerged based on whether the oocytes were BCB positive or negative (Fig. 1D).

### Co-expression analysis between oocytes and surrounding cumulus cells

**Fig. 2:**
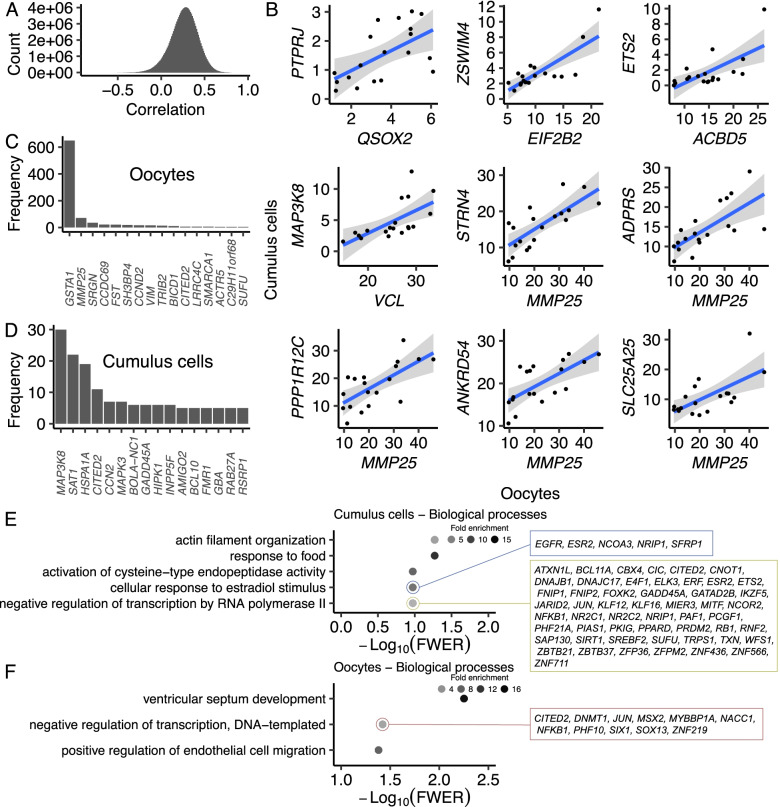
Gene co-expression networks between oocytes and surrounding cumulus cells. **A** Distribution of Pearson’s correlation coefficients for genes expressed in oocytes and cumulus cells. **B** Scatterplots with representative genes co-expressing (r $\ge$ 0.85, eFDR < 1 × 10^–5^) between oocytes and cumulus cells. Connectivity of genes significantly co-expressed (r $\ge$ 0.85, eFDR < 1 × 10^–5^) in (**C**) oocytes and (**D**) cumulus cells. Biological processes significantly enriched (FWER < 0.1) in genes co-expressed (r $\ge$ 0.85, eFDR < 1 × 10^–5^) between (**E**) cumulus cells and (**F**) oocytes

We quantified a Pearson’s correlation coefficient (r) between genes expressed in oocytes and in cumulus cells, using all 19 pairs of oocytes and corresponding cumulus cells. The median of all coefficients was 0.27 (Fig. 2A), which often indicates an inclination of co-expression between genes, when compared to a null distribution, which is centered at zero (Supplementary figure S2). Following this global trend, the lowest negative coefficient calculated was -0.83, but there were 1,125 pairs of genes showing coefficients $\ge$ 0.85 (eFDR < 1 × 10^–5^). Some examples of these highly correlated pairs of genes are depicted in Fig. 2B. These 1,125 pairs of genes showing a high degree ($\ge$ 0.85) of co-expression were composed of 163 and 832 oocyte and cumulus cells genes, respectively. Notably, the gene Glutathione S-Transferase Alpha 1 expressed in oocytes concentrated over one-half of all the connections with cumulus genes, followed by eight genes with more than ten connections (*BICD1*, *CCDC69*, *CCND2*, *FST*, *SH3BP4*, *SRGN*, *TRIB2*, *VIM*, Fig. 2C, Supplementary figure S3, Supplementary Table S3). By comparison, in cumulus cells, the gene Mitogen-Activated Protein Kinase Kinase Kinase 8 had the greatest number of connections with oocyte genes and only three other genes had more than ten connections (*CITED2*, *HSPA1A*, *SAT1*, Fig. 2D, Supplementary figure S3, Supplementary Table S3). Collectively, these results demonstrated a general co-regulation of genes between oocyte and cumulus cells, but only a few hub genes emerged in both compartments of the cumulus-oocyte complex.

Genes that form regulatory networks tend to group in functional clusters. Therefore, we tested whether there was enrichment of the genes in oocytes or cumulus cells that showed significant co-expression (r $\ge$ 0.85, eFDR < 1 × 10^–5^). In cumulus cells, there were five biological processes significantly enriched (FWER < 0.11, Supplementary Table S4), ‘actin filament organization’, ‘activation of cysteine-type endopeptidase activity’, ‘cellular response to estradiol stimulus’, ‘negative regulation of transcription by RNA polymerase II’, and ‘response to food’. In oocytes, the biological processes significantly enriched (FWER < 0.1, Supplementary Table S5) were ‘negative regulation of transcription, DNA-templated’, ‘positive regulation of endothelial cell migration’, and ‘ventricular septum development’. The presence of categories involved in transcription in both compartments (oocyte and cumulus cells) supports the existence of gene regulatory networks across an oocyte and the surrounding cumulus cells.

### Differential gene expression in oocytes and cumulus cells

**Fig. 3:**
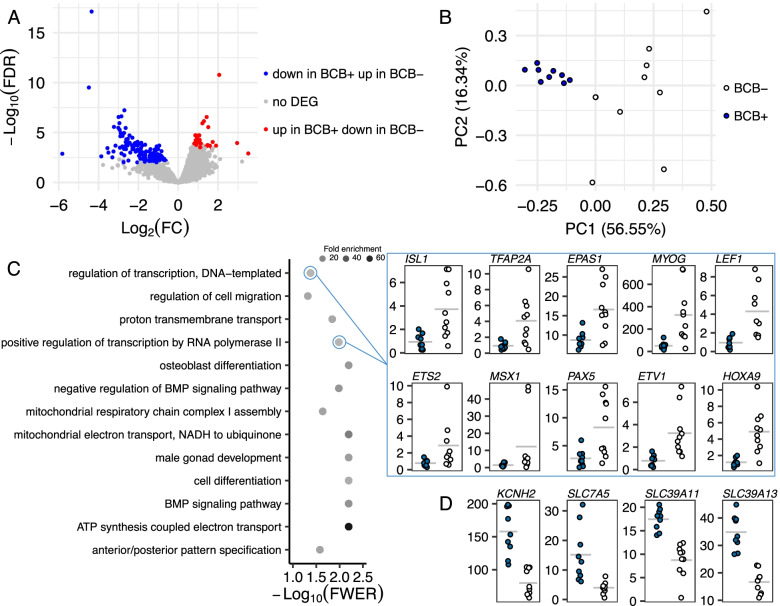
Differential transcript abundance in cumulus cells. **A** Depiction of the genes with significant differences in transcript abundance in cumulus cells surrounding oocytes categorized by BCB staining. **B** PCA plot of the 172 differentially expressed genes in cumulus cells collected from BCB positive or negative oocytes. **C** Biological processes categories enriched in 122 with greater transcript abundance in cumulus cells obtained from BCB negative oocytes relative to their counterparts. **D** Four genes with greater abundance in cumulus cells surrounding BCB positive oocytes and present in ‘transmembrane transport’

Next, we asked if gene transcript abundance was different between oocytes and cumulus cells obtained from oocytes classified based on BCB staining. Notably, there was no differential transcript abundance in oocytes separated by BCB staining (Supplementary Table S6). By comparison, 50 genes presented greater transcript abundance in cumulus cells obtained from BCB positive oocytes, and 122 presented greater transcript abundance in cumulus cells obtained from BCB negative oocytes (FDR < 0.01, Fig. 3A, Supplementary Table S7). Interestingly, cumulus cells surrounding BCB positive oocytes have a very similar pattern of transcript abundance for these 172 differentially expressed genes, whereas cumulus cells surrounding BCB negative oocytes have greater variability in transcript abundance (Fig. 3B).

We then tested these 172 differentially expressed genes in cumulus cells for enrichment of biological processes. There were several biological processes significantly enriched (FWER < 0.01) among the 122 with greater transcript abundance in cumulus cells obtained from BCB negative oocytes relative to their counterparts (Fig. 3C, Supplementary Table S8). A representative number of genes was observed in two categories associated with regulation of transcription (*EPAS1*, *ETS2*, *ETV1*, *HOXA9*, *ISL1*, *LEF1*, *MSX1*, *MYOG*, *PAX5*, *TFAP2A*), and another category highly represented was ‘cell differentiation’. It was also notable that ‘negative regulation of BMP signaling pathway’ (*SMAD6*, *BAMBI*, *SMAD7*, *SFRP1*) was enriched in the genes with greater transcript abundance in cumulus cells obtained from BCB negative oocytes (Fig. 3C, Supplementary Table S8). Testing for enriched categories among 50 genes with greater abundance in cumulus cells obtained from BCB positive oocytes revealed four genes present in ‘transmembrane transport’ (*KCNH2*, *SLC39A11*, *SLC39A13*, *SLC7A5*, FWER < 0.01, Fig. 3D, Supplementary Table S9).

### Differential gene co-expression between oocytes and cumulus cells

Given that cumulus cells have differential transcript abundance based on whether they surround an oocyte that is positive or negative for BCB staining, it was important to determine whether there is differential co-expression in oocytes and surrounding cumulus cells based on BCB staining outcomes. Similar to the approach used for all oocytes and cumulus cells, we calculated a Pearson’s correlation coefficient (r) for gene pairs for each group of cumulus-oocyte complexes. Because of the difference in sample size (BCB positive, *n* = 9; BCB negative, *n* = 10), we proceeded with coefficients that showed an eFDR < 1 × 10^–7^. This significance value was associated with *r* = 0.99 and 0.98 for cumulus-oocyte complexes classified as BCB positive and BCB negative (Supplementary figure S4). These low significance and high values of r were set so that we could make robust biological inferences about the results.

**Fig. 4:**
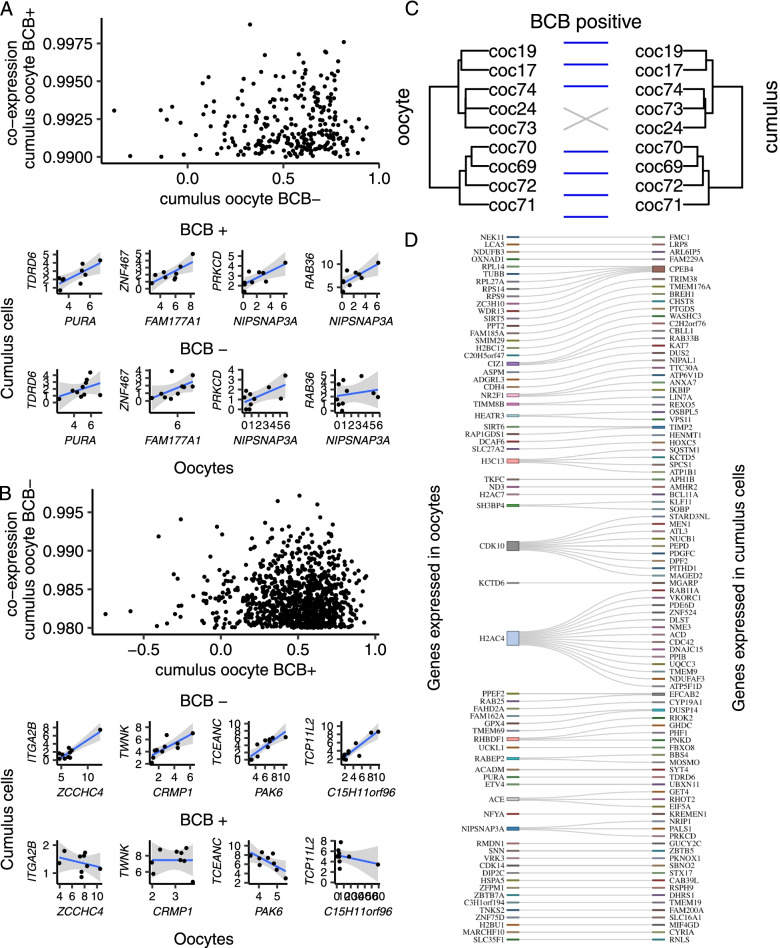
Differential co-expression in oocytes and surrounding cumulus cells classified by BCB staining. **A** Genes significantly co-expressed in BCB positive oocytes and surrounding cumulus cells, comparatively to complex-oocyte complexes obtained from BCB negative oocytes. **B** Genes significantly co-expressed in BCB negative oocytes and surrounding cumulus cells, comparatively to cumulus-oocyte complexes obtained from BCB positive oocytes. **C** Independent clustering of oocytes and cumulus cells using 75 and 108 genes, respectively, co-expressing at r $\ge$ 0.992. **D** Connectivity of the genes expressed in oocytes and surrounding cumulus cells co-expressing at r $\ge$ 0.992. Only genes annotated with a symbol are depicted on panel **D**

We identified 268 pairs of co-expressing genes (*r* > 0.99) in cumulus-oocyte complexes that were positive for BCB staining (*N* = 9 pairs of oocyte and cumulus cells). These were formed by 143 and 239 genes expressed in oocytes and cumulus cells, respectively. The same pair of genes showed r values that averaged 0.54, ranging from -0.38 to 0.93 in cumulus-oocyte complexes that were negative for BCB staining (Fig. 4A, Supplementary Table S10). In cumulus-oocyte complexes that were negative for BCB staining, we identified 1,004 pairs of co-expressing genes (*r* > 0.98), formed by 465 and 645 genes expressed in oocytes and cumulus cells, respectively. The same gene pairs showed r values that averaged 0.49, ranging from -0.75 to 0.94 in cumulus-oocyte complexes that were positive for BCB staining (Fig. 4B, Supplementary Table S11).

Subsequently, it was of interest to investigate if co-expressing genes would have a pattern of gene expression that is unique to the oocyte and surrounding cumulus cells. To that end, we tested if a set of co-expressed genes would produce an equivalent cluster of oocytes and cumulus cells, independently. Most notably, in cumulus-oocyte complexes that were positive for BCB staining, we identified a subset of 75 and 108 genes that formed 118 co-expressing pairs (r $\ge$ 0.992) that produced independent clusters that nearly mirrored each other (seven out of nine pairs, Fig. 4C). Most genes were co-expressed with one gene, with exception of *ACE*, *CDK10*, *CIZ1*, *H2AC4*, *H3C13*, *NIPSNAP3A,* and *RHBDF1* expressed in oocytes and with multiple connections with genes in cumulus cells. In cumulus cells, the gene *CPEB4* had multiple connections with genes expressed in oocytes (Fig. 4D).

We tested this subset of 75 and 108 genes for enrichment of biological processes. There was no significant enrichment for the 75 genes expressed in oocytes; however, it was notable that nine genes were annotated with categories related to the regulation of transcription (*DCAF6*, *ETV4*, *NFYA*, *NR2F1*, *PURA*, *SIRT6*, *ZBTB7A*, *ZFPM1*, *ZNF75D*, Supplementary Table S12). Among the genes expressed in cumulus cells, the biological processes ‘exocytosis’ (*LIN7A*, *RAB11A*, *STX17*, *SYT4*, *WASHC3*) and ‘cell projection organization' (*ATP6V1D*, *BBS4*, *RSPH9*, *TTC30A*) were significantly enriched (FWER < 0.05, Supplementary Table S13). Fifteen genes were associated with categories related to regulation of transcription (*BCL11A*, *DPF2*, *HOXC5*, *KAT7*, *KLF11*, *MAGED2*, *MEN1*, *MEPCE*, *NRIP1*, *PHF1*, *PKNOX1*, *SBNO2*, *SQSTM1*, *ZBTB5*, *ZNF524*).

## Discussion

Folliculogenesis involves a dynamic interaction between the oocyte and surrounding somatic cells [58, 59], which includes the exchange of paracrine signals [60, 61], transfer of metabolites [62], and possibly RNAs [63, 64] from cumulus cells to oocytes. Accounting for the fact that only cumulus-oocyte complexes of equivalent morphological quality (classes 1 and 2 based on the criteria presented in reference [65]) were analyzed here, the main findings of this study were: (i) BCB positive oocytes have fewer mtDNA copies relative to BCB negative oocytes; (ii) BCB positive and BCB negative oocytes have equivalent transcript abundance, but the transcriptome profile of surrounding cumulus cells differs; (iii) there is a pattern of co-expression between BCB positive oocytes and the surrounding cells that is unique to each cumulus-oocyte complex. The data corroborates our hypothesis raised previously [57] that the interaction between the oocyte and surrounding cumulus cells involves correlated transcript abundance between both compartments. We interpret that this co-expression is a result of coordinated gene regulatory networks between the oocyte and surrounding cumulus cells.

This study has some limitations. First, because we collected cumulus-oocyte complexes from ex vivo ovaries, our samples are not controlled for the stage of the follicle in the context of the follicular wave. Hence, we did not control for the impact that different stages of folliculogenesis have on the transcriptome profile of granulosa cells [66]. Second, we have a relatively limited sample size for our sequencing analysis. We produced transcriptome profile of 9 and 10 cumulus-oocyte complexes that were classified as BCB positive and BCB negative, respectively. Even though working with single oocytes allows us to capture the heterogeneity in a pool of growing antral follicles, otherwise not captured when samples are pooled [67], our sample size did not capture all of this heterogeneity. Lastly, we must point out that the classification of oocytes based on BCB staining separates oocytes based on a two-fold difference in their potential to produce a blastocyst (18% versus 9%). This separation limits the degree to which we can detect biologically relevant differences in transcript abundance above the noise. Nonetheless, our carefully designed experiment, robust procedures and thorough analytical approaches allowed us to obtain critical biological insights of the cumulus-oocyte interaction.

Our selection of COCs based on BCB staining confirmed previous findings [12, 16, 19, 23–25] that oocytes capable of degrading the BCB dye produced fewer blastocysts compared to the oocytes that cannot break down BCB, and thus remain blue. This result confirmed that our selection was effective in identifying oocytes with different levels of cytoplasmic maturation and developmental competence.

Mitochondria function [68, 69] and quantity [70] is critical for oocyte developmental potential. The abundance of active mitochondria in oocytes has been measured by fluorescence microscopy [15, 19] in BCB stained oocytes. Here, we quantified mtDNA in single oocytes as a proxy of the number of mitochondria in each oocyte. Our result that BCB negative oocytes have 1.36-fold more mtDNA compared to the BCB positive oocytes is aligned with the findings from Torner and colleagues [19]. We note, however, that these results are contradictory to the idea that the number of mitochondria in oocytes is either stable or increasing in oocytes throughout the folliculogenesis [71, 72] and that the greater the mtDNA copy number in oocytes, the greater their developmental competence [73].

We also interrogated the transcriptome of individual oocytes and cumulus cells. The profiling of these two compartments composing one biological unit (cumulus-oocyte complex) provides critical insight into the interaction between oocytes and surrounding cumulus cells [57]. Most notably, we identified genes expressed in cumulus cells that respond to estradiol stimulus, some of which are also associated with regulation of gene expression, with a high degree of co-expression with genes expressed in oocytes. This is one possible mechanism by which estradiol levels in the follicle can influence oocyte growth and maturation [74].

We also interrogated the differential transcript abundance in oocytes and cumulus cells based on BCB staining outcome. There was no differential transcript abundance between BCB positive and negative oocytes. Our result is consistent with the observation that transcription is quiescent in oocytes enclosed in late tertiary antral follicles [75]. However, this result is contradictory to two previous studies [19, 20]. Torner et al. [19] identified 185 genes with differential transcript abundance between BCB positive and negative bovine oocytes. Liu et al. [20] identified 155 genes with differential transcript abundance between BCB positive and negative porcine oocytes. We note that the first study [19] used pools of bovine oocytes and microarray hybridization on a chip containing approximately 2000 probes, whereas the second study [20] used single oocyte RNA sequencing, but did not adjust the nominal P-values for multiple hypothesis testing. It is also possible that we are using stringent criteria that will not infer significance in potential foreground signals; however, visual inspection of the data confirms the lack of differential transcript abundance. Lastly, it is also possible that our limited sample size combined with the limited separation of blastocyst yield between the two groups (18% vs 9%) did not provide sufficient power to detect signals of differential transcript abundance.

Different from our result in oocytes, we detected 172 genes with differential transcript abundance in cumulus cells based on the classification of oocytes for BCB staining. Only one of the genes inferred with differential transcript abundance in cumulus cells in this study has been associated with oocyte quality. Assidi and colleagues [76] detected greater transcript abundance of the gene Ring Finger Protein 121 (*RFP121*) in cumulus cells of developmentally competent oocytes. The gene *RFP121* had 1.8-fold more transcripts in cumulus cells associated with BCB positive oocytes, compared to those associated with BCB negative oocytes. These observations indicate that although BCB positive oocytes are twice as likely to develop into a blastocyst relative to BCB negative oocytes (see odds ratio, Table 2), the BCB separation may not be sufficient to detect putative biomarkers of oocyte quality by transcriptome analysis.

Gene ontology analysis of the 172 genes with differential transcript abundance in cumulus cells based on the classification of oocytes from BCB staining revealed important genes associated with the progress of maturation of oocytes. For instance, several biological processes enriched among the 122 genes with greater abundance in cumulus cells surrounding BCB negative oocytes are recognizably important for the progress of folliculogenesis. Some examples are the ‘negative regulation of BMP signaling pathway’, which was composed of genes that probably function to balance the effects of GDF9 and BMP15 secretion from the oocyte [77–80], ‘cell differentiation’, that is a critical part of the differentiation from granulosa cells [77], ‘ATP synthesis coupled electron transport’, which is a very important function of the cumulus cell since part of the ATP produced in cumulus cells enters the oocyte serving as a source of energy [81] and regulation of redox status in oocytes [82]. By contrast, the enrichment of the biological process ‘transmembrane transport’ among the 50 genes with greater abundance in cumulus cells obtained from BCB positive oocytes highlights the importance of the transport of micro and macro molecules between oocyte and surrounding cumulus cells [58], more specifically when the oocyte is fully grown.

The identification of co-expressing genes indicates the presence of functional links between genes [83], and our results presented in this study and a previously published study [57] confirm that oocytes and cumulus cells have coordinated gene regulatory networks. Our analysis of differential co-expression based on the oocyte growth phase (BCB negative versus BCB positive) showed that these regulatory networks are dynamic and change during the last steps of oocyte maturation within an antral follicle. Most notably, there is strong evidence that fully grown oocytes and the surrounding cells have tight regulation of gene transcription to the extent that the transcript abundance for several genes in both compartments (oocyte and cumulus cells) is specific to the unit (cumulus-oocyte complex).

There is also evidence that this co-expression is relevant to oocyte maturation. For instance, the enrichment of genes expressed in cumulus cells associated with ‘cell projection organization’ indicates that the oocyte may exert control of the genes responsible for the formation of the transzonal projections [63, 64]. Our results indicate that this tight gene regulatory network in fully grown oocytes and their surrounding cells is essential to the function of the cumulus-oocyte complex.

## Conclusions

Our results of the abundance of mtDNA in single oocytes are aligned with a previous report [19] that BCB positive oocytes have, on average, fewer mitochondria compared with BCB negative oocytes. Our results also show that BCB positive oocytes have a similar transcript pattern when compared to BCB negative oocytes if they are from middle to late tertiary follicles, but there is significant variation in the transcript abundance of several genes in the cumulus cells. In non-dominant antral follicles, the gene regulatory networks between oocytes and cumulus cells indicate regulation of gene transcript abundance that is unique to each of the cumulus-oocyte complexes. Those gene regulatory networks are likely essential for the oocyte’s health, and one can expect that their variation is connected to an oocyte’s developmental potential.

## Supplementary Information

**Additional file 1:** **Supplementary code.** Additional file containing the code used for data processing and analysis.

**Additional file 2:** **Supplementary figures.** Additional file containing supporting figures

**Additional file 3:** **Supplementary table S1.** Analysis of variance for the mtDNA copy number in oocytes separated by briliant cresyl blue

**Additional file 4:** **Supplementary table S2.** Linear hypothesis testing for the least square means for the mtDNA copy number in oocytes separated by briliant cresyl blue.

**Additional file 5:** **Supplementary table S3.** Significant co-expression (absolute *r*>0.85) between oocyte and cumulus cells.

**Additional file 6:** **Supplementary table S4.** Gene ontology enrichment analysis of genes expressed in cumulus cells and correlated with oocytes.

**Additional file 7:** **Supplementary table S5.** Gene ontology enrichment analysis of genes expressed in oocytes cells and correlated with cumulus cells.

**Additional file 8:** **Supplementary table S6.** Analysis of differential transcript abundance in oocytes classified by brilliant cresyl blue.

**Additional file 9:** **Supplementary table S7.** Analysis of differential transcript abundance in cumulus cells surrounding oocytes classified by brilliant cresyl blue.

**Additional file 10:** **Supplementary table S8.** Gene ontology enrichment analysis of genes with greater transcript abundance in cumulus cells surrounding BCB negative oocytes.

**Additional file 11:** **Supplementary table S9.** Gene ontology enrichment analysis of genes with lower transcript abundance in cumulus cells surrounding BCB negative oocytes.

**Additional file 12:** **Supplementary table S10.** Gene co-expression between BCB positive oocytes and cumulus cells.

**Additional file 13:** **Supplementary table S11.** Gene co-expression between BCB negative oocytes and cumulus cells.

**Additional file 14:** **Supplementary table S12.** Gene ontology enrichment analysis of genes expressed in BCB positive oocytes correlated with cumulus cells.

**Additional file 15:** **Supplementary table S13.** Gene ontology enrichment analysis of genes expressed in cumulus cells surrounding BCB positive oocytes.

## Data Availability

The datasets generated during the current study are available in the Gene Expression Omnibus repository [26], accession GSE199210. The codes used for all analytical procedures and processed data were deposited in the figshare repository [27], can be accessed at https://biase-lab.github.io/oocyte_cumulus_BCB/index.html, and are presented as Additional file.

## Abbreviations

BSA: Bovine serum albumin
BCB: Brilliant cresyl blue
CO_2_: Carbonic gas
NADP^+^: Co-enzyme nicotinamide adenine dinucleotide phosphate
CC: cumulus cells
CPM: counts per million
COC: Cumulus oocyte complex
°C: Degree Celsius
dNTPs: Diphosphate nucleotides
EGTA: Egtazic acid
eFDR: Empirical False Discovery Rate
FDR: False discovery rate
FBS: Fetal bovine serum
G6PDH: Glucose-6-Phosphate Dehydrogenase
H^+^: Hydrogen ion
IVM: In vitro maturation
IU: International Units
µg: Microgram
µl: Microliter
µM: Micromolar
mg: Miligram
ml: Mililiter
mM: Millimolar
mtDNA: Mitochondrial DNA
N_2_: Nitrogen
O_2_: Oxygen
PBS: Phosphate buffered saline
PEG: Polyethylene glycol
P: Probability
NaCl: Sodium chloride
SE: Standard error
SOF: Synthetic oviduct fluid
TCM: Tissue culture medium
TPM: Transcript per million
TMM: Trimmed mean of M values
