# Supplementary material for: Tight gene co-expression in BCB positive cattle oocytes and their surrounding cumulus cells
Source: Reprod Biol Endocrinol. 2022 Aug 13;20:119. doi: 10.1186/s12958-022-00994-3 (PMC9375383; doi:10.1186/s12958-022-00994-3)
Supplement: Supplementary file 1 — Additional file 1: Supplementary code. Additional file containing the code used for data processing and analysis. [file 12958_2022_994_MOESM1_ESM.html]

Supplementary code to Tight gene co-expression in BCB positive oocytes and their surrounding cumulus cells.


# Supplementary code to Tight gene co-expression in BCB positive oocytes and their surrounding cumulus cells.

#### Bailey N. Walker, Jada Nix, Chace Wilson, Mackenzie A. Marrella, Savannah L. Speckhart, Lydia Wooldridge, Con-Ning Yen, Jocelyn S. Bodmer, Laila T. Kirkpatrick, Sarah E.D. Moorey, David E. Gerrard, Alan Ealy, Fernando H. Biase

#### 2022-06-05

**Abstract**: Cytoplasmic and nuclear maturation of
oocytes, as well as interaction with the surrounding cumulus cells, are
important features relevant to the acquisition of developmental
competence. Brilliant cresyl blue (BCB) was utilized to distinguish
oocytes with low activity of the enzyme Glucose-6-Phosphate
Dehydrogenase, and thus separated fully grown (BCB positive) oocytes
from those in the growing phase (BCB negative). The BCB positive oocytes
were twice as likely to produce a blastocyst in vitro compared to BCB-
oocytes (P<0.01). We analyzed mitochondrial DNA (mtDNA) copy number
in single oocytes and determined that BCB negative oocytes have 1.3-fold
more copies than BCB positive oocytes (P=0.004). We also investigated
the transcriptome of oocytes and surrounding cumulus cells of BCB
positive versus BCB negative oocytes. There was no differential
transcript abundance of genes expressed in oocytes, however, 172 genes
were identified in cumulus cells with differential transcript abundance
(FDR<0.05) based on the BCB staining of their oocyte. Co-expression
analysis between oocytes and their surrounding cumulus cells revealed a
subset of genes whose co-expression in BCB positive oocytes (n=75) and
their surrounding cumulus cells (n=108) compose a unique profile of the
cumulus-oocyte complex. If oocytes transition from BCB negative to BCB
positive, there is a greater likelihood of producing a blastocyst, and a
reduction of mtDNA copies, but there is no systematic variation of
transcript abundance. Cumulus cells present changes in transcript
abundance, which reflects in a dynamic co-expression between the oocyte
and cumulus cells.

## Overview

Code produced by Fernando Biase. I created this file to permit
reproducibility of the findings described in the paper. Please direct
questions to Fernando Biase: ***fbiase*** at
***vt.edu***

Updated information may be obtained at www.biaselaboatory.com

The raw data is deposited on GEO repository under the following
access GSE199210.
For the reproduction of this code please download the .RData file
containing all the objects corresponding to the data used in this work:
2022\_03\_20\_oocyte\_cumulus\_BCB.RData

## Code

Use the tab below to navigate the different segments
of our code.

### Import files for reproducibility

```
library('biomaRt', lib.loc="/usr/lib/R/site-library")
cow<-useMart("ensembl", dataset = "btaurus_gene_ensembl", host="www.ensembl.org") 
annotation.ensembl.symbol<-getBM(attributes = c('ensembl_gene_id','external_gene_name','description','hgnc_symbol','gene_biotype','transcript_length','chromosome_name','start_position','end_position','strand'),  values = "*", mart = cow)

annotation.ensembl.symbol<-annotation.ensembl.symbol[order(annotation.ensembl.symbol$ensembl_gene_id, -annotation.ensembl.symbol$transcript_length),]
annotation.ensembl.symbol<-annotation.ensembl.symbol[!duplicated(annotation.ensembl.symbol$ensembl_gene_id),]
gene.length<-annotation.ensembl.symbol[,c( "ensembl_gene_id", "transcript_length" )]
annotation.GO.biomart<-getBM(attributes = c('ensembl_gene_id', 'external_gene_name','go_id','name_1006','namespace_1003'),  values = "*", mart = cow)

write.table(annotation.ensembl.symbol,file="/mnt/storage/lab_folder/shared_R_codes/fernando/BCB_oocyte_cumulus/resources/2021_12_31_annotation.ensembl.symbol.txt", sep = "\t",append = FALSE, quote = FALSE)
system('bzip2 --best /mnt/storage/lab_folder/shared_R_codes/fernando/BCB_oocyte_cumulus/resources/2021_12_31_annotation.ensembl.symbol.txt')

write.table(gene.length,file="/mnt/storage/lab_folder/shared_R_codes/fernando/BCB_oocyte_cumulus/resources/2021_12_31_gene.length.txt", sep = "\t",append = FALSE, quote = FALSE)
system('bzip2 --best /mnt/storage/lab_folder/shared_R_codes/fernando/BCB_oocyte_cumulus/resources/2021_12_31_gene.length.txt')

write.table(annotation.GO.biomart,file="/mnt/storage/lab_folder/shared_R_codes/fernando/BCB_oocyte_cumulus/resources/2021_12_31_annotation.GO.biomart.txt", sep = "\t",append = FALSE, quote = FALSE)
system('bzip2 --best /mnt/storage/lab_folder/shared_R_codes/fernando/BCB_oocyte_cumulus/resources/2021_12_31_annotation.GO.biomart.txt')
```

```
annotation.ensembl.symbol<-read.delim("/mnt/storage/lab_folder/shared_R_codes/fernando/BCB_oocyte_cumulus/resources/2021_12_31_annotation.ensembl.symbol.txt.bz2", header=TRUE, sep= "\t",row.names=1, stringsAsFactors = FALSE)
gene.length<-read.delim("/mnt/storage/lab_folder/shared_R_codes/fernando/BCB_oocyte_cumulus/resources/2021_12_31_gene.length.txt.bz2", header=TRUE, sep= "\t",row.names=1, stringsAsFactors = FALSE)
annotation.GO.biomart<-read.delim("/mnt/storage/lab_folder/shared_R_codes/fernando/BCB_oocyte_cumulus/resources/2021_12_31_annotation.GO.biomart.txt.bz2", header=TRUE, sep= "\t",row.names=1, stringsAsFactors = FALSE)

oocyte_count_data<-read.delim("/mnt/storage/lab_folder/shared_R_codes/fernando/BCB_oocyte_cumulus/results/2022_03_19_oocyte_count.txt.bz2", header = TRUE, row.names=1, sep = "\t")
cumulus_count_data<-read.delim("/mnt/storage/lab_folder/shared_R_codes/fernando/BCB_oocyte_cumulus/results/2022_03_19_cumulus_count.txt.bz2", header = TRUE, row.names=1, sep = "\t")

mtDNA_data<-readxl::read_excel("/mnt/storage/lab_folder/shared_R_codes/fernando/BCB_oocyte_cumulus/resources/ALL_mtDNA_Data_Complied_forR.xlsx")

source("/mnt/storage/lab_folder/shared_R_codes/fernando/BCB_oocyte_cumulus/resources/functions_plot_tanglegram.R")

save.image(file = "/mnt/storage/lab_folder/shared_R_codes/fernando/BCB_oocyte_cumulus/resources/2022_03_20_oocyte_cumulus_BCB.RData",compress= "bzip2", safe=TRUE)
```

Load the data and resources needed for reproducibility.

```
load(file = "/mnt/storage/lab_folder/shared_R_codes/fernando/BCB_oocyte_cumulus/resources/2022_03_20_oocyte_cumulus_BCB.RData")
```

### Analysis of blastocyst yield

```
.libPaths("/usr/lib/R/site-library")

library("tidyverse", quietly = TRUE, lib.loc="/usr/lib/R/site-library")
library("multcomp", quietly = TRUE, lib.loc="/usr/lib/R/site-library")
library("aod", quietly = TRUE, lib.loc="/usr/lib/R/site-library")
library("car", quietly = TRUE, lib.loc="/usr/lib/R/site-library")
library("emmeans", quietly = TRUE, lib.loc="/usr/lib/R/site-library")
library("lmtest", quietly = TRUE, lib.loc="/usr/lib/R/site-library")
```

Import the data of blastocyst development.

```
Input = ("
Replicate   Group   B   NB
1 BCB+ 11 59
1   BCB- 12 79
2   BCB+ 7  28
2   BCB-    3   18
2   C   10 65
3   BCB+ 16 49
3   BCB- 16 136
3   C 10 36
4 BCB+ 20 112
4 BCB- 6 125
")

Data = read.table(textConnection(Input),header=TRUE)
Data$Replicate<-as.factor(Data$Replicate)
Data$Group<-as.factor(Data$Group)
```

#### Blastocyst yield from COCs separated based on BCB staining

##### Table 1

Logistic regression to test the effect of BCB staining on blastocyst
yield, followed by the Wald test of significance.

```
model.log<-glm(cbind(B,NB) ~ Group + Replicate, family = binomial(link = "logit"), data = Data)
Anova(model.log,     type="III",      test.statistic="Wald")
```

```
## Analysis of Deviance Table (Type III tests)
## 
## Response: cbind(B, NB)
##             Df   Chisq Pr(>Chisq)    
## (Intercept)  1 69.8847  < 2.2e-16 ***
## Group        2 12.9639   0.001531 ** 
## Replicate    3  6.2416   0.100430    
## ---
## Signif. codes:  0 '***' 0.001 '**' 0.01 '*' 0.05 '.' 0.1 ' ' 1
```

Likelyhood test of the effect of BCB staining on blastocyst
yield.

```
full.model.log<-glm(cbind(B,NB) ~  Replicate + Group, family = binomial(link = "logit"), data = Data)
reduced.model.log<-glm(cbind(B,NB) ~  Replicate, family = binomial(link = "logit"), data = Data)
lrtest(full.model.log,reduced.model.log)
```

```
## Likelihood ratio test
## 
## Model 1: cbind(B, NB) ~ Replicate + Group
## Model 2: cbind(B, NB) ~ Replicate
##   #Df  LogLik Df  Chisq Pr(>Chisq)   
## 1   6 -22.015                        
## 2   4 -28.711 -2 13.394   0.001235 **
## ---
## Signif. codes:  0 '***' 0.001 '**' 0.01 '*' 0.05 '.' 0.1 ' ' 1
```

##### Table 2

Tukey test for multiple comparison of means, and the odds ratio.

```
summary(glht(model.log, linfct = mcp(Group = "Tukey")))
```

```
## 
##   Simultaneous Tests for General Linear Hypotheses
## 
## Multiple Comparisons of Means: Tukey Contrasts
## 
## 
## Fit: glm(formula = cbind(B, NB) ~ Group + Replicate, family = binomial(link = "logit"), 
##     data = Data)
## 
## Linear Hypotheses:
##                  Estimate Std. Error z value Pr(>|z|)    
## BCB+ - BCB- == 0   0.8432     0.2347   3.593  0.00083 ***
## C - BCB- == 0      0.5530     0.3471   1.593  0.24347    
## C - BCB+ == 0     -0.2903     0.3365  -0.863  0.65921    
## ---
## Signif. codes:  0 '***' 0.001 '**' 0.01 '*' 0.05 '.' 0.1 ' ' 1
## (Adjusted p values reported -- single-step method)
```

```
emmeans(model.log, ~Group, type='response') %>% multcomp::cld(Letters=letters)
```

```
##  Group   prob     SE  df asymp.LCL asymp.UCL .group
##  BCB-  0.0908 0.0153 Inf    0.0649     0.126  a    
##  C     0.1479 0.0353 Inf    0.0911     0.231  ab   
##  BCB+  0.1883 0.0240 Inf    0.1457     0.240   b   
## 
## Results are averaged over the levels of: Replicate 
## Confidence level used: 0.95 
## Intervals are back-transformed from the logit scale 
## P value adjustment: tukey method for comparing a family of 3 estimates 
## Tests are performed on the log odds ratio scale 
## significance level used: alpha = 0.05 
## NOTE: Compact letter displays can be misleading
##       because they show NON-findings rather than findings.
##       Consider using 'pairs()', 'pwpp()', or 'pwpm()' instead.
```

```
emmeans(model.log, pairwise ~ Group, type='response')
```

```
## $emmeans
##  Group   prob     SE  df asymp.LCL asymp.UCL
##  BCB-  0.0908 0.0153 Inf    0.0649     0.126
##  BCB+  0.1883 0.0240 Inf    0.1457     0.240
##  C     0.1479 0.0353 Inf    0.0911     0.231
## 
## Results are averaged over the levels of: Replicate 
## Confidence level used: 0.95 
## Intervals are back-transformed from the logit scale 
## 
## $contrasts
##  contrast        odds.ratio    SE  df null z.ratio p.value
##  (BCB-) / (BCB+)      0.430 0.101 Inf    1  -3.593  0.0010
##  (BCB-) / C           0.575 0.200 Inf    1  -1.593  0.2485
##  (BCB+) / C           1.337 0.450 Inf    1   0.863  0.6640
## 
## Results are averaged over the levels of: Replicate 
## P value adjustment: tukey method for comparing a family of 3 estimates 
## Tests are performed on the log odds ratio scale
```

### Analysis of mtDNA copy

#### Mitochondrial DNA abundance in single oocytes classified by BCB staining

Analyze the mtDNA data produced by real-time quantitative polymerase
chain reaction.

```
mtDNA_data$Plate<-as.factor(mtDNA_data$Plate)
mtDNA_data$Group<-as.factor(mtDNA_data$Group)
```

##### Supplemental table 1

Analyze the variance of the mtDNA counts obtained by real-time
quantitative polymerase chain reaction.

```
mtDNAmodel<-lm(Copy_number ~   Plate + Group ,contrasts=list(Plate='contr.sum', Group ='contr.sum'), data = mtDNA_data)
summary(mtDNAmodel)
```

```
## 
## Call:
## lm(formula = Copy_number ~ Plate + Group, data = mtDNA_data, 
##     contrasts = list(Plate = "contr.sum", Group = "contr.sum"))
## 
## Residuals:
##      Min       1Q   Median       3Q      Max 
## -1602259  -520845   -79130   321959  2168324 
## 
## Coefficients:
##             Estimate Std. Error t value Pr(>|t|)    
## (Intercept)  1699487      85792  19.809  < 2e-16 ***
## Plate1       -201216     122398  -1.644  0.10401    
## Plate2        -31977     120206  -0.266  0.79089    
## Group1        258963      88428   2.929  0.00441 ** 
## ---
## Signif. codes:  0 '***' 0.001 '**' 0.01 '*' 0.05 '.' 0.1 ' ' 1
## 
## Residual standard error: 793500 on 82 degrees of freedom
## Multiple R-squared:  0.1123, Adjusted R-squared:  0.0798 
## F-statistic: 3.457 on 3 and 82 DF,  p-value: 0.02012
```

```
car::Anova(mtDNAmodel,type='III')
```

```
## Anova Table (Type III tests)
## 
## Response: Copy_number
##                 Sum Sq Df  F value    Pr(>F)    
## (Intercept) 2.4708e+14  1 392.4159 < 2.2e-16 ***
## Plate       2.5142e+12  2   1.9965  0.142352    
## Group       5.4000e+12  1   8.5763  0.004408 ** 
## Residuals   5.1631e+13 82                       
## ---
## Signif. codes:  0 '***' 0.001 '**' 0.01 '*' 0.05 '.' 0.1 ' ' 1
```

##### Supplemental table 2

Post hoc Tukey test for comparison of means.

```
summary(glht(mtDNAmodel, linfct = mcp(Group = "Tukey")))
```

```
## 
##   Simultaneous Tests for General Linear Hypotheses
## 
## Multiple Comparisons of Means: Tukey Contrasts
## 
## 
## Fit: lm(formula = Copy_number ~ Plate + Group, data = mtDNA_data, 
##     contrasts = list(Plate = "contr.sum", Group = "contr.sum"))
## 
## Linear Hypotheses:
##                          Estimate Std. Error t value Pr(>|t|)   
## Positive - Negative == 0  -517926     176855  -2.929  0.00441 **
## ---
## Signif. codes:  0 '***' 0.001 '**' 0.01 '*' 0.05 '.' 0.1 ' ' 1
## (Adjusted p values reported -- single-step method)
```

```
emmeans(mtDNAmodel, pairwise ~ Group, type='response')
```

```
## $emmeans
##  Group     emmean     SE df lower.CL upper.CL
##  Negative 1958450 126717 82  1706370  2210529
##  Positive 1440524 119592 82  1202618  1678429
## 
## Results are averaged over the levels of: Plate 
## Confidence level used: 0.95 
## 
## $contrasts
##  contrast            estimate     SE df t.ratio p.value
##  Negative - Positive   517926 176855 82   2.929  0.0044
## 
## Results are averaged over the levels of: Plate
```

```
emmeans(mtDNAmodel, ~Group, type='response') %>% multcomp::cld(Letters=letters)
```

```
##  Group     emmean     SE df lower.CL upper.CL .group
##  Positive 1440524 119592 82  1202618  1678429  a    
##  Negative 1958450 126717 82  1706370  2210529   b   
## 
## Results are averaged over the levels of: Plate 
## Confidence level used: 0.95 
## significance level used: alpha = 0.05 
## NOTE: Compact letter displays can be misleading
##       because they show NON-findings rather than findings.
##       Consider using 'pairs()', 'pwpp()', or 'pwpm()' instead.
```

##### Supplemental figure 1

```
ggplot(data=mtDNA_data, aes(x=Group, y=Copy_number))+
  geom_boxplot(color="blue",width=0.15, outlier.color = "red",outlier.shape = 4,outlier.size = 1.5)+
  geom_jitter( width=0.15)+
  scale_y_continuous(name="mtDNA copy number")+
  scale_x_discrete(name=NULL,labels=c("Negative"="BCB negative", "Positive"="BCB positive"))+
  ggsignif::geom_signif(annotations = c("0.0044 "), y_position=4500000, xmin = c(1),xmax=c(2),tip_length = 0.01, textsize=4)+
  theme_classic()+
  theme(
    axis.title = element_text(size=12, color="black"),
    axis.text = element_text(size=12, color="black")
  )
```

Supplemental figure 1. Mitochondrial DNA copy number
quantified in single oocytes classified by brilliant cresyl blue
staining.

### Analysis RNA-seq data

```
.libPaths("/usr/lib/R/site-library")
library("edgeR", quietly = TRUE, lib.loc="/usr/lib/R/site-library")
library("DESeq2", quietly = TRUE, lib.loc="/usr/lib/R/site-library")
library("DEsingle", quietly = TRUE, lib.loc="/usr/lib/R/site-library")
library("biomaRt",quietly = TRUE, lib.loc="/usr/lib/R/site-library")
library("goseq" ,quietly = TRUE, lib.loc="/usr/lib/R/site-library")
library("VennDiagram", quietly = TRUE, lib.loc="/usr/lib/R/site-library")
library("ggpubr", quietly = TRUE, lib.loc="/usr/lib/R/site-library")
library("Rtsne", quietly = TRUE,lib.loc="/usr/lib/R/site-library")
library("MEGENA",quietly = TRUE, lib.loc="/usr/lib/R/site-library")
library("DGCA", quietly = TRUE, lib.loc="/usr/lib/R/site-library")
library('dplyr', quietly = TRUE,lib.loc="/usr/lib/R/site-library")
library("ggplot2", quietly = TRUE, lib.loc="/usr/lib/R/site-library")
library("ggrepel", quietly = TRUE, lib.loc="/usr/lib/R/site-library")
library("gridExtra",quietly = TRUE , lib.loc="/usr/lib/R/site-library")
library("readr" , quietly = TRUE, lib.loc="/usr/lib/R/site-library")
library("ggfortify" , quietly = TRUE, lib.loc="/usr/lib/R/site-library")
library("cowplot" , quietly = TRUE, lib.loc="/usr/lib/R/site-library")
library("tidyverse", quietly = TRUE, lib.loc="/usr/lib/R/site-library")
library("WGCNA", quietly = TRUE, lib.loc="/usr/lib/R/site-library")
library("reshape2", quietly = TRUE, lib.loc="/usr/lib/R/site-library")
library("foreach", quietly = TRUE, lib.loc="/usr/lib/R/site-library")
library("doParallel", quietly = TRUE, lib.loc="/usr/lib/R/site-library")
library("bigmemory", quietly = TRUE, lib.loc="/usr/lib/R/site-library")
library("gtools", quietly = TRUE, lib.loc="/usr/lib/R/site-library")
library("data.table", quietly = TRUE, lib.loc="/usr/lib/R/site-library")
library("flashClust", quietly = TRUE, lib.loc="/usr/lib/R/site-library")
library("dendextend", quietly = TRUE, lib.loc="/usr/lib/R/site-library")
library('vegan', quietly = TRUE, lib.loc="/usr/lib/R/site-library")
library("ade4", quietly = TRUE, lib.loc="/usr/lib/R/site-library")
library("gridGraphics", quietly = TRUE, lib.loc="/usr/lib/R/site-library")
library("networkD3", quietly = TRUE, lib.loc="/usr/lib/R/site-library")
```

```
annotation.ensembl.symbol<-annotation.ensembl.symbol[annotation.ensembl.symbol$gene_biotype %in% c('protein_coding', 'lncRNA'),]

oocyte_count_data_annotated<-merge(oocyte_count_data,annotation.ensembl.symbol, by.x="row.names", by.y="ensembl_gene_id", all.x=FALSE, all.y=FALSE)
oocyte_gene_length<-oocyte_count_data_annotated$transcript_length
oocyte_count_data<-oocyte_count_data_annotated[,c(2:20)]
rownames(oocyte_count_data)<-oocyte_count_data_annotated$Row.names

cumulus_count_data_annotated<-merge(cumulus_count_data,annotation.ensembl.symbol, by.x="row.names", by.y="ensembl_gene_id", all.x=FALSE, all.y=FALSE)
cumulus_gene_length<-cumulus_count_data_annotated$transcript_length
cumulus_count_data<-cumulus_count_data_annotated[,c(2:20)]
rownames(cumulus_count_data)<-cumulus_count_data_annotated$Row.names
```

#### Transcriptome profiling of single oocytes and the corresponding cumulus cells

```
x <- oocyte_count_data / oocyte_gene_length
oocyte_tpm <- as.data.frame(t( t(x) * 1e6 / colSums(x) ))
oocyte_fpkm<-as.data.frame(edgeR::rpkm(oocyte_count_data, oocyte_gene_length))
oocyte_cpm<-as.data.frame(edgeR::cpm(oocyte_count_data))
oocyte_cpm_filtered<-oocyte_cpm[rowSums(oocyte_cpm>2) > 8,]
oocyte_tpm_filtered<-oocyte_fpkm[rowSums(oocyte_tpm>1) > 8,]

genes_expressed_oocyte<-intersect(rownames(oocyte_cpm_filtered), rownames(oocyte_tpm_filtered))
oocyte_tpm_filtered<-oocyte_tpm_filtered[rownames(oocyte_tpm_filtered) %in% genes_expressed_oocyte,]
oocyte_cpm_filtered<-oocyte_cpm_filtered[rownames(oocyte_cpm_filtered) %in% genes_expressed_oocyte,]
oocyte_count_filtered<-oocyte_count_data[rownames(oocyte_count_data) %in% genes_expressed_oocyte,]

oocyte_tpm_filtered<-oocyte_tpm_filtered[complete.cases(oocyte_tpm_filtered),]
oocyte_count_filtered<-oocyte_count_filtered[complete.cases(oocyte_count_filtered),]
#dim(oocyte_tpm_filtered)
#dim(oocyte_count_filtered)

x <- cumulus_count_data / cumulus_gene_length
cumulus_tpm <- as.data.frame(t( t(x) * 1e6 / colSums(x) ))
cumulus_fpkm<-as.data.frame(edgeR::rpkm(cumulus_count_data, cumulus_gene_length))
cumulus_cpm<-as.data.frame(edgeR::cpm(cumulus_count_data))
cumulus_cpm_filtered<-cumulus_cpm[rowSums(cumulus_cpm>2) > 8,]
cumulus_tpm_filtered<-cumulus_tpm[rowSums(cumulus_tpm>1) > 8,]

genes_expressed_cumulus<-intersect(rownames(cumulus_cpm_filtered), rownames(cumulus_tpm_filtered))
cumulus_tpm_filtered<-cumulus_tpm_filtered[rownames(cumulus_tpm_filtered) %in% genes_expressed_cumulus,]
cumulus_cpm_filtered<-cumulus_cpm_filtered[rownames(cumulus_cpm_filtered) %in% genes_expressed_cumulus,]
cumulus_count_filtered<-cumulus_count_data[rownames(cumulus_count_data) %in% genes_expressed_cumulus,]

cumulus_tpm_filtered<-cumulus_tpm_filtered[complete.cases(cumulus_tpm_filtered),]
cumulus_count_filtered<-cumulus_count_filtered[complete.cases(cumulus_count_filtered),]
#dim(cumulus_tpm_filtered)
#dim(cumulus_count_filtered)

oocyte_tpm_filtered_annotated<-merge(oocyte_tpm_filtered, annotation.ensembl.symbol, by.x="row.names", by.y="ensembl_gene_id", all.x=TRUE, all.y=FALSE)
#write_delim(oocyte_tpm_filtered_annotated, "/mnt/storage/lab_folder/shared_R_codes/fernando/BCB_oocyte_cumulus/results/2022_01_18_oocyte_tpm_filtered_annotated.txt", delim = "\t", quote =  "none")

cumulus_tpm_filtered_annotated<-merge(cumulus_tpm_filtered, annotation.ensembl.symbol, by.x="row.names", by.y="ensembl_gene_id", all.x=TRUE, all.y=FALSE)
#write_delim(cumulus_tpm_filtered_annotated, "/mnt/storage/lab_folder/shared_R_codes/fernando/BCB_oocyte_cumulus/results/2022_01_18_cumulus_tpm_filtered_annotated.txt", delim = "\t", quote =  "none")
```

```
gene_numbers_venn_diagram<-venn.diagram(
  list("oocyte"=rownames(oocyte_count_filtered), "cumulus cells"=rownames(cumulus_count_filtered)), cex=1,
  scaled= FALSE, filename = NULL, output=TRUE,
  col="transparent",
  fill=c("#56B4E9", "#E69F00"),
  cat.pos=c(-15,15),
  cat.cex=c(1,1))
#ggdraw(gene_numbers_venn_diagram)
```

```
oocyte_group<-factor(c("BCBpos","BCBpos","BCBneg","BCBpos","BCBneg","BCBneg","BCBneg","BCBpos","BCBpos","BCBpos","BCBpos","BCBpos","BCBpos","BCBneg","BCBneg","BCBneg","BCBneg","BCBneg","BCBneg"), levels=c("BCBneg","BCBpos"))

cumulus_group<-factor(c("BCBpos", "BCBpos", "BCBneg",   "BCBpos",       "BCBneg",   "BCBneg",   "BCBneg",   "BCBpos", "BCBpos", "BCBpos",   "BCBpos",   "BCBpos",   "BCBpos",   "BCBneg",   "BCBneg",   "BCBneg",   "BCBneg",   "BCBneg", "BCBneg"), levels=c( "BCBneg", "BCBpos"))

data_plot<-(cbind(data.frame(t(oocyte_tpm_filtered)), oocyte_group, names=colnames(oocyte_tpm_filtered)))
res.prcomp <-stats::prcomp(data_plot[,-c(dim(data_plot)[2]-1,dim(data_plot)[2])], scale. = TRUE)
pca_plot_oocyte<-ggplot2::autoplot(res.prcomp, data=data_plot, fill='oocyte_group', shape=21, size=2)+
scale_fill_manual(name=NULL,values=c( "white", "blue"))+
ggrepel::geom_text_repel(label=data_plot$names, size=2)+
ggtitle("oocytes")+
theme_bw()+
theme(
axis.text=element_text(size=12, color="black"),
axis.title=element_text(size=12, color="black"),
legend.position="none"
)

data_plot<-(cbind(data.frame(t(cumulus_tpm_filtered)), cumulus_group, names=colnames(cumulus_tpm_filtered)))
res.prcomp <-stats::prcomp(data_plot[,-c(dim(data_plot)[2]-1,dim(data_plot)[2])], scale. = TRUE)
pca_plot_cumulus<-ggplot2::autoplot(res.prcomp, data=data_plot, fill='cumulus_group', shape=21, size=2)+
scale_fill_manual(name=NULL,values=c( "white", "blue"))+
ggrepel::geom_text_repel(label=data_plot$names, size=2)+
ggtitle("cumulus cells")+
theme_bw()+
theme(
axis.text=element_text(size=12, color="black"),
axis.title=element_text(size=12, color="black"),
legend.direction="horizontal"
)

legend<-ggpubr::get_legend(pca_plot_cumulus)

data_plot<-(cbind(data.frame(t(cumulus_tpm_filtered)), cumulus_group, names=colnames(cumulus_tpm_filtered)))
res.prcomp <-stats::prcomp(data_plot[,-c(dim(data_plot)[2]-1,dim(data_plot)[2])], scale. = TRUE)
pca_plot_cumulus<-ggplot2::autoplot(res.prcomp, data=data_plot, fill='cumulus_group', shape=21, size=2)+
scale_fill_manual(name=NULL,values=c( "white", "blue"))+
ggrepel::geom_text_repel(label=data_plot$names, size=2)+
ggtitle("cumulus cells")+
theme_bw()+
theme(
axis.text=element_text(size=12, color="black"),
axis.title=element_text(size=12, color="black"),
legend.position="none"
)
```

##### Figure 1

```
 plot_grid(plot_grid(NULL,ggdraw(gene_numbers_venn_diagram),NULL, nrow=1,labels = c("A", "B"),label_size = 12, label_fontface = "plain", rel_widths=c(1.5,0.5,0.02)), 
           plot_grid(plot_grid(pca_plot_oocyte,pca_plot_cumulus, labels = c("C", "D",""), label_fontface = "plain",label_size = 12), legend, nrow=2,rel_heights=c(1,0.1)), nrow=2, rel_heights=c(0.5,1))
```

Figure 1. Transcriptome analysis of cumulus-oocyte
complexes. (A) Schematics of sample classification based on BCB
staining. (B) Number of protein-coding or long noncoding genes with
transcripts quantified in single oocytes and corresponding cumulus
cells. (C) Principal component analysis of oocytes. (D) Principal
component analysis of cumulus cells. For both C and D, empty circles
indicate BCB negative, and blue circles indicate BCB positive.

#### Co-expression analysis between oocytes and surrounding cumulus cells

Calculate the co-expression for all 19 pairs of
oocyte and cumulus cells.

```
#oocyte_count_filtered
#cumulus_count_filtered

lib_size <- base::colSums(oocyte_count_filtered)
norm_factors <- calcNormFactors(object = oocyte_count_filtered, lib.size = lib_size, method = "TMM")
CTF_normalized_oocyte <- sweep(oocyte_count_filtered, 2, norm_factors, "/")
asinh_transf_CTF_normalized_oocyte<-asinh(CTF_normalized_oocyte)

lib_size <- base::colSums(cumulus_count_filtered)
norm_factors <- calcNormFactors(object = cumulus_count_filtered, lib.size = lib_size, method = "TMM")
CTF_normalized_cumulus <- sweep(cumulus_count_filtered, 2, norm_factors, "/")
asinh_transf_CTF_normalized_cumulus<-asinh(CTF_normalized_cumulus)
```

##### Supplemental figure 2

Obtain null distribution. For reproducibility the
image used in the paper will be uploaded here.

```
sample_a<-data.frame()
random_a<-data.frame()
random_b<-data.frame(matrix(NA, nrow = 150076995, ncol = 1))
for (i in (1:5)){

randomization<-sample(1:19,replace=F)
  
random_a<-WGCNA::cor(t(asinh_transf_CTF_normalized_oocyte[,randomization]),  t(asinh_transf_CTF_normalized_cumulus),  use = "pairwise.complete.obs", method="pearson")
random_a<-reshape2::melt(random_a) 
random_b<-cbind(random_b,random_a$value)

sample_a<-rbind(sample_a,randomization)
}

random_b<-random_b[,-1]

summary(random_b)

plot_null_distribution<-ggplot()+
geom_histogram(aes(x=random_b[,1]), data=random_b, binwidth=0.01, alpha=0.2)+
geom_histogram(aes(x=random_b[,2]), data=random_b, binwidth=0.01, alpha=0.2)+
geom_histogram(aes(x=random_b[,3]), data=random_b, binwidth=0.01, alpha=0.2)+
geom_histogram(aes(x=random_b[,4]), data=random_b, binwidth=0.01, alpha=0.2)+
geom_histogram(aes(x=random_b[,5]), data=random_b, binwidth=0.01, alpha=0.2)+
scale_y_continuous(name="Count")+
scale_x_continuous(name="Correlation")+
theme(
panel.background = element_blank(),
panel.grid.major = element_blank(),
plot.background = element_blank(),
axis.line = element_line(color="black"),
axis.text = element_text(color="black", size=10),
axis.title.y=element_text(color="black", size=10,hjust=0),
axis.title.x = element_text(color="black", size=10)
)

pdf(file="/mnt/storage/lab_folder/shared_R_codes/fernando/BCB_oocyte_cumulus/results/Supplemental_fig_2022_03_23.pdf", width=4, height=4, bg="transparent" , onefile=TRUE)
plot_null_distribution
dev.off()
```

```
ggdraw() + draw_image(magick::image_read_pdf("/mnt/storage/lab_folder/shared_R_codes/fernando/BCB_oocyte_cumulus/results/Supplemental_fig_2022_03_23.pdf", density = 300))
```

Estimate the values for empirical false discovery
rate.

```
setwd("/mnt/storage/lab_folder/shared_R_codes/fernando/BCB_oocyte_cumulus/results/")
asinh_transf_CTF_normalized_oocyte_big_matrix<- as.big.matrix(t(asinh_transf_CTF_normalized_oocyte) , type = "double", 
                    separated = FALSE, 
                    backingfile = "asinh_transf_CTF_normalized_oocyte.bin", 
                    descriptorfile = "asinh_transf_CTF_normalized_oocyte.desc", 
                    share=TRUE)
# get a description of the matrix
mdesc_oocyte <- describe(asinh_transf_CTF_normalized_oocyte_big_matrix)

asinh_transf_CTF_normalized_cumulus_big_matrix <- as.big.matrix(t(asinh_transf_CTF_normalized_cumulus), type = "double", 
                      separated = FALSE, 
                      backingfile = "asinh_transf_CTF_normalized_cumulus.bin", 
                      descriptorfile = "asinh_transf_CTF_normalized_cumulus.desc",
                      share=TRUE)
# get a description of the matrix
mdesc_cumulus<- describe(asinh_transf_CTF_normalized_cumulus_big_matrix)

rand<-20000

sequence.correlation<-seq(0.80, 0.92, 0.01)

results <- filebacked.big.matrix(13,rand, type="double", init=0, separated=FALSE, 
                                 backingfile="incidence_matrix.bin",
                                 descriptor="incidence_matrix.desc")
mdesc_result<- describe(results)

cl <- makeCluster(34)
registerDoParallel(cl)

results[,]<- foreach(i = 1:rand, .combine='cbind', .inorder=FALSE,.packages=c("WGCNA","bigmemory"), .noexport=c("asinh_transf_CTF_normalized_oocyte_big_matrix", "asinh_transf_CTF_normalized_cumulus_big_matrix"), .verbose=FALSE ) %dopar% {
    
    require(bigmemory)
    
    oocyte<- attach.big.matrix("asinh_transf_CTF_normalized_oocyte.desc")
    cumulus<- attach.big.matrix("asinh_transf_CTF_normalized_cumulus.desc")
    random<-WGCNA::cor(oocyte[sample(1:19,replace=FALSE),],  cumulus[,],  use = "pairwise.complete.obs", method="pearson")
    
    matrix( c(length(which(abs(random) > 0.80)) , 
    length(which(abs(random) > 0.81)),
    length(which(abs(random) > 0.82)),
    length(which(abs(random) > 0.83)),
    length(which(abs(random) > 0.84)),
    length(which(abs(random) > 0.85)),
    length(which(abs(random) > 0.86)),
    length(which(abs(random) > 0.87)),
    length(which(abs(random) > 0.88)),
    length(which(abs(random) > 0.89)),
    length(which(abs(random) > 0.90)),
    length(which(abs(random) > 0.91)),
    length(which(abs(random) > 0.92))), nrow = 13, ncol = 1, byrow = TRUE)
    
  }
  
stopCluster(cl)

total.rand <- 20000 * 150076995

qvalue_oocyte_cc<-data.frame(correlation = sequence.correlation, e.pvalue= (rowSums(results[,])+1)/(total.rand+1))

write.table(qvalue_oocyte_cc, file = "qvalue_oocyte_cc_2022_02_01.txt",quote = TRUE, sep = "\t",row.names = TRUE,col.names = TRUE)

system("rm asinh_transf_CTF_normalized_oocyte_pos.bin")
system("rm asinh_transf_CTF_normalized_oocyte_pos.desc")
system("rm asinh_transf_CTF_normalized_cumulus_pos.bin")
system("rm asinh_transf_CTF_normalized_cumulus_pos.desc")
system("rm incidence_matrix.bin")
system("rm incidence_matrix.desc")
```

```
read.delim("/mnt/storage/lab_folder/shared_R_codes/fernando/BCB_oocyte_cumulus/results/qvalue_oocyte_cc_2022_02_01.txt", header=TRUE, sep= "\t",row.names=1, stringsAsFactors = FALSE)
```

```
##    correlation     e.pvalue
## 1         0.80 5.012540e-05
## 2         0.81 3.493701e-05
## 3         0.82 2.392004e-05
## 4         0.83 1.605620e-05
## 5         0.84 1.053819e-05
## 6         0.85 6.744419e-06
## 7         0.86 4.190283e-06
## 8         0.87 2.517387e-06
## 9         0.88 1.452871e-06
## 10        0.89 7.984028e-07
## 11        0.90 4.129484e-07
## 12        0.91 1.988023e-07
## 13        0.92 8.680011e-08
```

##### Supplemental table 3

```
#colnames(asinh_transf_CTF_normalized_oocyte)
#colnames(asinh_transf_CTF_normalized_cumulus)

cor_oocyte_cc<-corAndPvalue(t(asinh_transf_CTF_normalized_oocyte),t(asinh_transf_CTF_normalized_cumulus), use = "pairwise.complete.obs",alternative="two.sided")

cor_oocyte_cc_value<-reshape2::melt(cor_oocyte_cc$cor)
cor_oocyte_cc_value<-as.data.table(cor_oocyte_cc_value)
cor_oocyte_cc_p_value<-reshape2::melt(cor_oocyte_cc$p)
cor_oocyte_cc_p_value<-as.data.table(cor_oocyte_cc_p_value)
cor_oocyte_cc<-cbind(cor_oocyte_cc_value,cor_oocyte_cc_p_value)
rm(cor_oocyte_cc_p_value,cor_oocyte_cc_value)
cor_oocyte_cc<-cor_oocyte_cc[,c(1,2,3,6)]
colnames(cor_oocyte_cc)<-c("gene_oocyte","gene_cumulus","correlation","p_value")
#head(cor_oocyte_cc)
cor_oocyte_cc<-setDT(cor_oocyte_cc[order(cor_oocyte_cc$p_value),])

#cor_oocyte_cc[abs(cor_oocyte_cc$correlation)>0.9]
#dim(cor_oocyte_cc[abs(cor_oocyte_cc$correlation)>0.85])
cor_oocyte_cc085<-cor_oocyte_cc[abs(cor_oocyte_cc$correlation)>0.85]
cor_oocyte_cc085_annotated<-setDT(cor_oocyte_cc085)
cor_oocyte_cc085_annotated$oocyte_gene_symbol <- annotation.ensembl.symbol$external_gene_name[match(cor_oocyte_cc085_annotated$gene_oocyte,annotation.ensembl.symbol$ensembl_gene_id)]
cor_oocyte_cc085_annotated$cumulus_gene_symbol <- annotation.ensembl.symbol$external_gene_name[match(cor_oocyte_cc085_annotated$gene_cumulus,annotation.ensembl.symbol$ensembl_gene_id)]

#write.table(cor_oocyte_cc085_annotated, file = "cor_oocyte_cc085_annotated_2022_02_02.txt",quote = TRUE, sep = "\t",row.names = TRUE,col.names = TRUE)
```

Plot the histogram for all values of coefficient.

```
plot_histogram<-ggplot()+
geom_histogram(aes(x=correlation), data=cor_oocyte_cc, binwidth=0.01)+
scale_y_continuous(name="Count")+
scale_x_continuous(name="Correlation")+
theme(
panel.background = element_blank(),
panel.grid.major = element_blank(),
plot.background = element_blank(),
axis.line = element_line(color="black"),
axis.text = element_text(color="black", size=10),
axis.title.y=element_text(color="black", size=10,hjust=0),
axis.title.x = element_text(color="black", size=10)
)
```

Obtain gene connectivity.

```
gene_connectivity<- cor_oocyte_cc085_annotated %>% dplyr::count(oocyte_gene_symbol) %>% dplyr::arrange(desc(n))
gene_connectivity<-gene_connectivity[gene_connectivity$n > 4,]
gene_connectivity<-gene_connectivity[!(gene_connectivity$oocyte_gene_symbol ==""),]
gene_connectivity$oocyte_gene_symbol<-factor(gene_connectivity$oocyte_gene_symbol, levels=c(gene_connectivity$oocyte_gene_symbol))


gene_connectivity_oocyte<-ggplot()+
geom_col( aes(x=oocyte_gene_symbol, y=n), data=gene_connectivity)+
 scale_x_discrete(name=NULL)+
  scale_y_continuous(name="Frequency")+
  #scale_y_break(c(100, 600 ) )+
  annotate("text",x=9, y=400, label="Oocytes")+
  theme_classic()+
  theme(
    axis.text.x = element_text(angle=90, face="italic", size=7, vjust=0.5,hjust = 0),
    axis.text.y = element_text(color="black", size=10),
    axis.title.y=element_text(color="black", size=10,hjust=0),
    axis.title.x=element_blank()
  )

gene_connectivity<- cor_oocyte_cc085_annotated %>% dplyr::count(cumulus_gene_symbol) %>% dplyr::arrange(desc(n))
gene_connectivity<-gene_connectivity[gene_connectivity$n > 4,]
gene_connectivity<-gene_connectivity[!(gene_connectivity$cumulus_gene_symbol ==""),]
gene_connectivity$cumulus_gene_symbol<-factor(gene_connectivity$cumulus_gene_symbol, levels=c(gene_connectivity$cumulus_gene_symbol))


gene_connectivity_cumulus<-ggplot()+
geom_col( aes(x=cumulus_gene_symbol, y=n), data=gene_connectivity)+
 scale_x_discrete(name=NULL)+
  scale_y_continuous(name="Frequency")+
  annotate("text",x=9, y=20, label="Cumulus cells")+
  theme_classic()+
  theme(
    axis.text.x = element_text(angle=90, face="italic", size=7, vjust=0.5,hjust = 0),
    axis.text.y = element_text(color="black", size=10),
    axis.title.y=element_text(color="black", size=10,hjust=0),
    axis.title.x=element_blank()
  )
```

Plot examples of co-expressed genes beteen oocyte and
cumulus cells.

```
cor_oocyte_cc_filtered<-cor_oocyte_cc[abs(cor_oocyte_cc$correlation)>0.85,]
cor_oocyte_cc_filtered$oocyte_gene_symbol <- annotation.ensembl.symbol$external_gene_name[match(cor_oocyte_cc_filtered$gene_oocyte,annotation.ensembl.symbol$ensembl_gene_id)]
cor_oocyte_cc_filtered$cumulus_gene_symbol <- annotation.ensembl.symbol$external_gene_name[match(cor_oocyte_cc_filtered$gene_cumulus,annotation.ensembl.symbol$ensembl_gene_id)]

cor_oocyte_cc_filtered$angle<-NA
cor_oocyte_cc_filtered$r.squared<-NA
cor_oocyte_cc_filtered$rmse<-NA
cor_oocyte_cc_filtered$fstatistic<-NA

for (i in seq(dim(cor_oocyte_cc_filtered)[1]))
{
gene_oocyte<-as.character(cor_oocyte_cc_filtered$gene_oocyte)[i]
gene_cumulus<-as.character(cor_oocyte_cc_filtered$gene_cumulus)[i]

gene_symbol_oocyte<-as.character(cor_oocyte_cc_filtered$oocyte_gene_symbol)[i]
gene_symbol_cumulus<-as.character(cor_oocyte_cc_filtered$cumulus_gene_symbol)[i]

expression_oocyte<-data.frame(expression_oocyte=t(oocyte_tpm_filtered[rownames(oocyte_tpm_filtered)==gene_oocyte,])[,1])
expression_cumulus<-data.frame( expression_cumulus=t(cumulus_tpm_filtered[rownames(cumulus_tpm_filtered)==gene_cumulus,])[,1])

model_oocyte_cc<-lm(expression_oocyte$expression_oocyte ~ expression_cumulus$expression_cumulus)

cor_oocyte_cc_filtered$angle[i]<-unname(atan(coef(model_oocyte_cc)[2]) * (180 / pi))
cor_oocyte_cc_filtered$r.squared[i]<-summary(model_oocyte_cc)$adj.r.squared
cor_oocyte_cc_filtered$rmse[i]<-sqrt(mean(model_oocyte_cc$residuals^2))
cor_oocyte_cc_filtered$fstatistic[i]<-unname(summary(model_oocyte_cc)$fstatistic[1])
}

cor_oocyte_cc_filtered<-cor_oocyte_cc_filtered[cor_oocyte_cc_filtered$angle > 40 & cor_oocyte_cc_filtered$angle < 60,]
cor_oocyte_cc_filtered<-cor_oocyte_cc_filtered[order(cor_oocyte_cc_filtered$rmse , decreasing = FALSE),]
cor_oocyte_cc_filtered<-cor_oocyte_cc_filtered[cor_oocyte_cc_filtered$r.squared>0.3,]
cor_oocyte_cc_filtered<-cor_oocyte_cc_filtered[c(2:4,6:14),]

dataframegraph<-data.frame()

for (i in c(1:12)){

gene_oocyte<-as.character(cor_oocyte_cc_filtered$gene_oocyte)[i]
gene_cumulus<-as.character(cor_oocyte_cc_filtered$gene_cumulus)[i]

gene_symbol_oocyte<-as.character(cor_oocyte_cc_filtered$oocyte_gene_symbol)[i]
gene_symbol_cumulus<-as.character(cor_oocyte_cc_filtered$cumulus_gene_symbol)[i]

expression_oocyte<-data.frame(expression_oocyte=t(oocyte_tpm_filtered[rownames(oocyte_tpm_filtered)==gene_oocyte,])[,1])
expression_cumulus<-data.frame( expression_cumulus=t(cumulus_tpm_filtered[rownames(cumulus_tpm_filtered)==gene_cumulus,])[,1])

graph<-i

dataframegraph<-rbind(data.frame(gene_oocyte,gene_cumulus,gene_symbol_oocyte,gene_symbol_cumulus,expression_oocyte,expression_cumulus,graph),dataframegraph)

}

plots<-list()

for (i in c(1:12)){

plots[[i]]<- ggplot(dataframegraph[dataframegraph$graph==i,], aes(x=expression_oocyte, y= expression_cumulus ))+
geom_point()+
geom_smooth(method='lm', formula= y~x)+
scale_x_continuous(name=dataframegraph[dataframegraph$graph==i,]$gene_symbol_oocyte[1])+
scale_y_continuous(name=dataframegraph[dataframegraph$graph==i,]$gene_symbol_cumulus[1])+
theme_classic(base_size=12)+
theme(
axis.title=element_text(face="italic", size=10),
axis.text=element_text(size=10, color="black"),
)

}

y.grob <- textGrob("cumulus cells", gp=gpar(fontface="plain", col="black", fontsize=10), rot=90)
x.grob <- textGrob("oocyte", gp=gpar(fontface="plain", col="black", fontsize=10))

plot_regr<-plot_grid(  grid.arrange(arrangeGrob(plot_grid(plotlist=plots, nrow=3), left = y.grob, bottom = x.grob)))
```

Test cumulus genes (significantly co-expressed with
oocytes) for enrichment of biological processes.

##### Supplemental table 4

```
gene.length<-read.delim("/mnt/storage/lab_folder/shared_R_codes/fernando/BCB_oocyte_cumulus/resources/2021_12_31_gene.length.txt.bz2", header=TRUE, sep= "\t",row.names=1, stringsAsFactors = FALSE)

all_genes<-data.frame( gene=rownames(cumulus_count_filtered), stringsAsFactors=FALSE )
rownames(all_genes)<-all_genes$gene
N_expressed_genes<-length(all_genes$gene)

gene.length<-gene.length[gene.length$ensembl_gene_id %in% all_genes$gene,]

annotation.genelength.biomart_vector<-gene.length$transcript_length
names(annotation.genelength.biomart_vector)<-gene.length$ensembl_gene_id

annotation.GO.BP.biomart<-annotation.GO.biomart[annotation.GO.biomart$namespace_1003=="biological_process", c(1,3)]
annotation.GO.BP.biomart<-annotation.GO.BP.biomart[annotation.GO.BP.biomart$ensembl_gene_id %in% rownames(all_genes),]
annotation.GO.MF.biomart<-annotation.GO.biomart[annotation.GO.biomart$namespace_1003=="molecular_function", c(1,3)]
annotation.GO.MF.biomart<-annotation.GO.MF.biomart[annotation.GO.MF.biomart$ensembl_gene_id %in% rownames(all_genes),]
```

```
test.genes<-data.frame(a=unique(cor_oocyte_cc[abs(cor_oocyte_cc$correlation)>0.85]$gene_cumulus), stringsAsFactors=FALSE)
all_genes_numeric<-as.integer(all_genes$gene %in%test.genes$a)
names(all_genes_numeric)<-all_genes$gene

N_sig_genes<-length(test.genes$a)

set.seed(88972)
pwf<-nullp(all_genes_numeric, bias.data=annotation.genelength.biomart_vector, plot.fit=FALSE ) 
GO_BP_Cats_cumulus_corr_oocyte<-goseq(pwf,gene2cat=annotation.GO.BP.biomart, method ="Sampling", repcnt = 5000, use_genes_without_cat=FALSE)
#GO_BP_Cats_cumulus_corr_oocyte<-goseq(pwf,gene2cat=annotation.GO.BP.biomart, method ="Wallenius", use_genes_without_cat=FALSE)
GO_BP_Cats_cumulus_corr_oocyte<-GO_BP_Cats_cumulus_corr_oocyte[GO_BP_Cats_cumulus_corr_oocyte$numDEInCat>3,]
GO_BP_Cats_cumulus_corr_oocyte$FWER<-p.adjust(GO_BP_Cats_cumulus_corr_oocyte$over_represented_pvalue, method ="holm")
GO_BP_Cats_cumulus_corr_oocyte<-GO_BP_Cats_cumulus_corr_oocyte[with(GO_BP_Cats_cumulus_corr_oocyte, order(FWER,over_represented_pvalue, -numDEInCat)), ]
#head(GO_BP_Cats_cumulus_corr_oocyte, n=20)

GO_BP_Cats_cumulus_corr_oocyte$fold_enrichment<-(GO_BP_Cats_cumulus_corr_oocyte$numDEInCat/N_sig_genes)/(GO_BP_Cats_cumulus_corr_oocyte$numInCat/N_expressed_genes)
annotation.GO.BP.biomart_testgenes<-annotation.GO.BP.biomart[annotation.GO.BP.biomart$ensembl_gene_id %in% test.genes$a, ]
GO_BP_Cats_cumulus_corr_oocyte<-merge(GO_BP_Cats_cumulus_corr_oocyte,annotation.GO.BP.biomart_testgenes, by.x="category", by.y="go_id", all.x=TRUE, all.y=FALSE)
GO_BP_Cats_cumulus_corr_oocyte<-merge(GO_BP_Cats_cumulus_corr_oocyte, annotation.ensembl.symbol, by.x="ensembl_gene_id", by.y="ensembl_gene_id", all=FALSE, all.x=TRUE, all.y=FALSE)
GO_BP_Cats_cumulus_corr_oocyte<-GO_BP_Cats_cumulus_corr_oocyte[with(GO_BP_Cats_cumulus_corr_oocyte, order(FWER,term)), ]
GO_BP_Cats_cumulus_corr_oocyte<-GO_BP_Cats_cumulus_corr_oocyte[GO_BP_Cats_cumulus_corr_oocyte$FWER <= 0.11,]
GO_BP_Cats_cumulus_corr_oocyte$term<-factor(GO_BP_Cats_cumulus_corr_oocyte$term, levels=c(rev(unique(GO_BP_Cats_cumulus_corr_oocyte$term))))
#write.table(GO_BP_Cats_cumulus_corr_oocyte, file= "/mnt/storage/lab_folder/shared_R_codes/fernando/BCB_oocyte_cumulus/results/2022_02_02_GO_BP_Cats_cumulus_corr_oocyte_at_fdr01.txt", append = FALSE, quote = FALSE, sep = "\t" ,row.names = FALSE)
```

##### Supplemental table 5

Test oocyte genes (significantly co-expressed with
cumulus cells) for enrichment of biological processes.

```
gene.length<-read.delim("/mnt/storage/lab_folder/shared_R_codes/fernando/BCB_oocyte_cumulus/resources/2021_12_31_gene.length.txt.bz2", header=TRUE, sep= "\t",row.names=1, stringsAsFactors = FALSE)

all_genes<-data.frame( gene=rownames(oocyte_count_filtered), stringsAsFactors=FALSE )
rownames(all_genes)<-all_genes$gene
N_expressed_genes<-length(all_genes$gene)

gene.length<-gene.length[gene.length$ensembl_gene_id %in% all_genes$gene,]

annotation.genelength.biomart_vector<-gene.length$transcript_length
names(annotation.genelength.biomart_vector)<-gene.length$ensembl_gene_id

annotation.GO.BP.biomart<-annotation.GO.biomart[annotation.GO.biomart$namespace_1003=="biological_process", c(1,3)]
annotation.GO.BP.biomart<-annotation.GO.BP.biomart[annotation.GO.BP.biomart$ensembl_gene_id %in% rownames(all_genes),]
annotation.GO.MF.biomart<-annotation.GO.biomart[annotation.GO.biomart$namespace_1003=="molecular_function", c(1,3)]
annotation.GO.MF.biomart<-annotation.GO.MF.biomart[annotation.GO.MF.biomart$ensembl_gene_id %in% rownames(all_genes),]
```

```
test.genes<-data.frame(a=unique(cor_oocyte_cc[abs(cor_oocyte_cc$correlation)>0.85]$gene_oocyte), stringsAsFactors=FALSE)
all_genes_numeric<-as.integer(all_genes$gene %in%test.genes$a)
names(all_genes_numeric)<-all_genes$gene

N_sig_genes<-length(test.genes$a)

set.seed(87175)
pwf<-nullp(all_genes_numeric, bias.data=annotation.genelength.biomart_vector, plot.fit=FALSE ) 
GO_BP_Cats_oocyte_corr_cumulus<-goseq(pwf,gene2cat=annotation.GO.BP.biomart, method ="Sampling", repcnt = 5000, use_genes_without_cat=FALSE)
GO_BP_Cats_oocyte_corr_cumulus<-GO_BP_Cats_oocyte_corr_cumulus[GO_BP_Cats_oocyte_corr_cumulus$numDEInCat>3,]
GO_BP_Cats_oocyte_corr_cumulus$FWER<-p.adjust(GO_BP_Cats_oocyte_corr_cumulus$over_represented_pvalue, method ="holm")
GO_BP_Cats_oocyte_corr_cumulus<-GO_BP_Cats_oocyte_corr_cumulus[with(GO_BP_Cats_oocyte_corr_cumulus, order(FWER,over_represented_pvalue, -numDEInCat)), ]
#head(GO_BP_Cats_oocyte_corr_cumulus, n=20)

GO_BP_Cats_oocyte_corr_cumulus$fold_enrichment<-(GO_BP_Cats_oocyte_corr_cumulus$numDEInCat/N_sig_genes)/(GO_BP_Cats_oocyte_corr_cumulus$numInCat/N_expressed_genes)
annotation.GO.BP.biomart_testgenes<-annotation.GO.BP.biomart[annotation.GO.BP.biomart$ensembl_gene_id %in% test.genes$a, ]
GO_BP_Cats_oocyte_corr_cumulus<-merge(GO_BP_Cats_oocyte_corr_cumulus,annotation.GO.BP.biomart_testgenes, by.x="category", by.y="go_id", all.x=TRUE, all.y=FALSE)
GO_BP_Cats_oocyte_corr_cumulus<-merge(GO_BP_Cats_oocyte_corr_cumulus, annotation.ensembl.symbol, by.x="ensembl_gene_id", by.y="ensembl_gene_id", all=FALSE, all.x=TRUE, all.y=FALSE)
GO_BP_Cats_oocyte_corr_cumulus<-GO_BP_Cats_oocyte_corr_cumulus[GO_BP_Cats_oocyte_corr_cumulus$FWER<0.1,]
GO_BP_Cats_oocyte_corr_cumulus<-GO_BP_Cats_oocyte_corr_cumulus[with(GO_BP_Cats_oocyte_corr_cumulus, order(FWER,term)), ]
GO_BP_Cats_oocyte_corr_cumulus$term<-factor(GO_BP_Cats_oocyte_corr_cumulus$term, levels=c(rev(unique(GO_BP_Cats_oocyte_corr_cumulus$term))))
#write.table(GO_BP_Cats_oocyte_corr_cumulus, file= "/mnt/storage/lab_folder/shared_R_codes/fernando/BCB_oocyte_cumulus/results/2022_02_01_GO_BP_Cats_oocyte_corr_cumulus_at_fdr01.txt", append = FALSE, quote = FALSE, sep = "\t" ,row.names = FALSE)
```

```
GO_oocyte_plot<-ggplot(data=GO_BP_Cats_oocyte_corr_cumulus, aes(x=term, y=-log10(FWER)))+
  geom_point(aes(color=fold_enrichment))+
  scale_y_continuous(name=bquote(-Log[10](FWER)), limits=c(1,2.5))+
  coord_flip()+
  scale_colour_gradient(name= "Fold enrichment",low = "#B8B8B8", high = "#080808", na.value = NA,limits = c(1, 16))+
  scale_x_discrete(name=NULL)+
  theme_classic() + 
  ggtitle("Oocytes - Biological processes") +
  theme(
    legend.direction = "horizontal",
    legend.position = c(0.9,1),
    legend.key.size = unit(0.5, 'mm'),
    legend.text=element_text(size=5,margin = margin(t = 0, r = 0, b = 0, l = 0, unit = "cm")),
    legend.title=element_text(size=5),
    legend.background=element_blank(),
    axis.text.x = element_text(color="black", size=10),
    axis.text.y = element_text(color="black", size=8),
    axis.title.y = element_blank(),
    axis.title.x = element_text(vjust=1,margin = margin(t = 0, r = 0, b = 0, l = 0, unit = "cm"), size = 10),
    plot.margin=unit(c(1,5,0,0), units="mm"),
    plot.title = element_text(size=8)
  ) + guides(color=guide_legend(title.position="top",title.hjust =0.5))

GO_cumulus_plot<-ggplot(data=GO_BP_Cats_cumulus_corr_oocyte, aes(x=term, y=-log10(FWER)))+
  geom_point(aes(color=fold_enrichment))+
  scale_y_continuous(name=bquote(-Log[10](FWER)), limits=c(0,2))+
  coord_flip()+
  scale_colour_gradient(name= "Fold enrichment",low = "#B8B8B8", high = "#080808", na.value = NA,limits = c(1, 15))+
  scale_x_discrete(name=NULL)+
  ggtitle("Cumulus cells - Biological processes") +
  theme_classic() + 
  theme(
    legend.direction = "horizontal",
    legend.position = c(0.9,0.96),
    legend.key.size = unit(0.5, 'mm'),
    legend.text=element_text(size=5,margin = margin(t = 0, r = 0, b = 0, l = 0, unit = "cm")),
    legend.title=element_text(size=5),
    legend.background=element_blank(),
    axis.text.x = element_text(color="black", size=10),
    axis.text.y = element_text(color="black", size=8),
    axis.title.y = element_blank(),
    axis.title.x = element_text(vjust=1,margin = margin(t = 0, r = 0, b = 0, l = 0, unit = "cm"), size = 10),
    plot.margin=unit(c(1,5,0,0), units="mm"),
    plot.title = element_text(size=8)
  ) + guides(color=guide_legend(title.position="top",title.hjust =0.5))
```

##### Figure 2

```
cowplot::plot_grid(
  cowplot::plot_grid(cowplot::plot_grid(plot_histogram, gene_connectivity_oocyte, gene_connectivity_cumulus, nrow =3, ncol=1, rel_heights=c(0.7,1,1),labels = c("A", "C","D"), label_fontface = "plain", label_size = 12 ),cowplot::plot_grid(plot_regr , label_fontface = "plain", label_size = 12 , labels = c("B")) ,ncol=2,rel_widths=c(0.5,1)),
  cowplot::plot_grid(cowplot::plot_grid(GO_cumulus_plot,GO_oocyte_plot, nrow =2, align = 'v',labels = c("E", "F"), label_fontface = "plain", label_size = 12), NULL, rel_widths=c(1,0.5)),
  nrow=2, rel_heights=c(1,0.6))
```

Figure 2. Gene co-expression networks between oocytes
and surrounding cumulus cells. (A) Distribution of Pearson’s correlation
coefficients for genes expressed in oocytes and cumulus cells. (B)
Scatterplots with representative genes co-expressing (r ≥ 0.85, eFDR
< 1x10^-5^) between oocytes and cumulus cells. Connectivity of genes
significantly co-expressed (r ≥ 0.85, eFDR < 1x10^-5^) in (C) oocytes
and (D) cumulus cells. Biological processes significantly enriched
(FWER<0.1) in genes co-expressed (r ≥ 0.85, eFDR < 1x10^-5^) between
(E) cumulus cells and (F) oocytes.

```
rm(plot_histogram,gene_connectivity_oocyte,gene_connectivity_cumulus,GO_cumulus_plot,GO_oocyte_plot,GO_plot,volcano_plot_deg_cc,pca_plot_deg_cc,plot_1,plot_2,plot_regr)
```

#### Differential gene expression in oocytes and cumulus cells

Test for differential transcript abundance in oocytes
or cumulus cells based on oocyte BCB coloration.

##### Supplemental table 6

DEG oocyte

```
oocyte_design<-(model.matrix(~oocyte_group))
oocyte_dge<-DGEList(count=oocyte_count_filtered, group=oocyte_group)
oocyte_dge<-estimateDisp(oocyte_dge, oocyte_design, robust=TRUE)
oocyte_dge<- glmFit(oocyte_dge, oocyte_design)
oocyte_dge<- glmLRT(oocyte_dge)
oocyte_dge_edger<- topTags(oocyte_dge, adjust.method = "fdr", n=Inf)$table

rm(oocyte_dge)
oocyte_colData<- data.frame("group"=oocyte_group)
rownames(oocyte_colData)<-colnames(oocyte_count_filtered)
oocyte_dge<-DESeqDataSetFromMatrix(countData=oocyte_count_filtered,colData=oocyte_colData, design= ~ group)
oocyte_dge<-DESeq(oocyte_dge)
oocyte_dge_DESeq<-results(oocyte_dge, contrast=c("group", "BCBpos", "BCBneg"), pAdjustMethod="fdr", tidy=TRUE)
rm(oocyte_dge)

merged_dge_oocyte_edger_DESeq<-merge(oocyte_dge_edger,oocyte_dge_DESeq, by.x='row.names', by.y="row")

merged_dge_oocyte_edger_DESeq_annotated<-merge(merged_dge_oocyte_edger_DESeq,annotation.ensembl.symbol, by.x="Row.names", by.y= "ensembl_gene_id", all.x=TRUE, all.y=FALSE)

#write.table(merged_dge_oocyte_edger_DESeq_annotated, file= "/mnt/storage/lab_folder/shared_R_codes/fernando/BCB_oocyte_cumulus/results/2022_03_08_merged_dge_oocyte_edger_DESeq_annotated.txt", append = FALSE, quote = FALSE, sep = "\t" ,row.names = FALSE)
```

##### Supplemental table 7

DEG cumulus cells

```
cumulus_design<-(model.matrix(~cumulus_group))
cumulus_dge<-DGEList(count=cumulus_count_filtered, group=cumulus_group)
cumulus_dge<-estimateDisp(cumulus_dge, cumulus_design, robust=TRUE)
cumulus_dge<- glmFit(cumulus_dge, cumulus_design)
cumulus_dge<- glmLRT(cumulus_dge)
cumulus_dge_edger<- topTags(cumulus_dge, adjust.method = "fdr", n=Inf)$table

rm(cumulus_dge)
cumulus_colData<- data.frame("group"=cumulus_group)
rownames(cumulus_colData)<-colnames(cumulus_count_filtered)
cumulus_dge<-DESeqDataSetFromMatrix(countData=cumulus_count_filtered,colData=cumulus_colData, design= ~ group)
cumulus_dge<-DESeq(cumulus_dge)
cumulus_dge_DESeq<-results(cumulus_dge, contrast=c("group", "BCBpos", "BCBneg"), pAdjustMethod="fdr", tidy=TRUE)
rm(cumulus_dge)

merged_dge_cumulus_edger_DESeq<-merge(cumulus_dge_edger,cumulus_dge_DESeq, by.x='row.names', by.y="row")

merged_dge_cumulus_edger_DESeq_annotated<-merge(merged_dge_cumulus_edger_DESeq,annotation.ensembl.symbol, by.x="Row.names", by.y= "ensembl_gene_id", all.x=TRUE, all.y=FALSE)

#write.table(merged_dge_cumulus_edger_DESeq_annotated, file= "/mnt/storage/lab_folder/shared_R_codes/fernando/BCB_oocyte_cumulus/results/2022_03_08_merged_dge_cumulus_edger_DESeq.txt", append = FALSE, quote = FALSE, sep = "\t" ,row.names = FALSE)

merged_dge_cumulus_edger_DESeq_annotated<-merged_dge_cumulus_edger_DESeq_annotated[(merged_dge_cumulus_edger_DESeq_annotated$FDR<0.01) & (merged_dge_cumulus_edger_DESeq_annotated$padj <0.01 ),]
#write.table(merged_dge_cumulus_edger_DESeq_annotated, file= "/mnt/storage/lab_folder/shared_R_codes/fernando/BCB_oocyte_cumulus/results/2022_03_08_merged_dge_cumulus_edger_DESeq.txt", append = FALSE, quote = FALSE, sep = "\t" ,row.names = FALSE)
```

```
oocyte_gene_annotation<-merge(merged_dge_oocyte_edger_DESeq, annotation.ensembl.symbol, by.x="Row.names", by.y="ensembl_gene_id", all.y=FALSE)
cumulus_gene_annotation<-merge(merged_dge_cumulus_edger_DESeq, annotation.ensembl.symbol, by.x="Row.names", by.y="ensembl_gene_id", all.y=FALSE)

cumulus_gene_annotation<-cumulus_gene_annotation[!is.na(cumulus_gene_annotation$logFC),]
cumulus_gene_annotation$logFC<-as.numeric(cumulus_gene_annotation$logFC)
cumulus_gene_annotation<-cumulus_gene_annotation[!is.na(cumulus_gene_annotation$logFC),]
cumulus_gene_annotation$diffexpressed <- "NO"
cumulus_gene_annotation$diffexpressed[cumulus_gene_annotation$logFC > 0 & cumulus_gene_annotation$FDR < 0.01 & cumulus_gene_annotation$padj < 0.01] <- "UP"
cumulus_gene_annotation$diffexpressed[cumulus_gene_annotation$logFC < 0 & cumulus_gene_annotation$FDR < 0.01 & cumulus_gene_annotation$padj < 0.01] <- "DOWN"
cumulus_gene_annotation$plot_label<-NA
cumulus_gene_annotation$plot_label[(abs(cumulus_gene_annotation$logFC) > 2)  & (cumulus_gene_annotation$FDR < 0.01) &  (cumulus_gene_annotation$padj < 0.01)]<-cumulus_gene_annotation$external_gene_name[(abs(cumulus_gene_annotation$logFC) > 2)  & (cumulus_gene_annotation$FDR < 0.01) &  (cumulus_gene_annotation$padj < 0.01)]

volcano_plot_deg_cc<-ggplot(data=cumulus_gene_annotation, aes(x=logFC, y=-log10(FDR),col=diffexpressed)) + 
  geom_point(size=0.5) + 
  scale_color_manual(name=NULL, values=c("blue", "gray", "red"), labels=c("down in BCB+ up in BCB-", "no DEG", "up in BCB+ down in BCB-"))+
  scale_y_continuous(name=bquote(-Log[10](FDR)))+
  scale_x_continuous(name=bquote(Log[2](FC)))+
  #ggrepel::geom_text_repel(label=cumulus_gene_annotation$plot_label, size=2)+
  theme_minimal()+
  theme(legend.position="right",
        axis.text = element_text(color="black", size=10),
        axis.title = element_text(color="black", size=10),
        legend.text = element_text(color="black", size=8, margin = margin(l = -10)),
        plot.margin=unit(c(0,0,3,0), "mm"),
        legend.box.margin=margin(0,0,0,-20))
```

```
cumulus_tpm_filtered_deg<-cumulus_tpm_filtered[rownames(cumulus_tpm_filtered) %in% cumulus_gene_annotation[(cumulus_gene_annotation$FDR<0.01) & (cumulus_gene_annotation$padj<0.01) ,1] , ]
data_plot<-(cbind(data.frame(t(cumulus_tpm_filtered_deg)), cumulus_group, names=colnames(cumulus_tpm_filtered_deg)))
res.prcomp <-stats::prcomp(data_plot[,-c(dim(data_plot)[2]-1,dim(data_plot)[2])], scale. = TRUE)
pca_plot_deg_cc<-ggplot2::autoplot(res.prcomp, data=data_plot, fill='cumulus_group', shape=21, size=1)+
scale_fill_manual(name=NULL,values=c( "white", "blue"), labels=c("BCB-", "BCB+"))+
#ggrepel::geom_text_repel(label=data_plot$names)+
theme_minimal(base_size=12)+
theme(legend.position="right",
        axis.text = element_text(color="black", size=10),
        axis.title = element_text(color="black", size=10),
        legend.text = element_text(color="black", size=8, margin = margin(l = -10)),
        plot.margin=unit(c(0,0,3,5), "mm"),
        legend.box.margin=margin(0,0,0,-20))
```

```
gene.length<-read.delim("/mnt/storage/lab_folder/shared_R_codes/fernando/BCB_oocyte_cumulus/resources/2021_12_31_gene.length.txt.bz2", header=TRUE, sep= "\t",row.names=1, stringsAsFactors = FALSE)
all_genes<-data.frame( gene=rownames(cumulus_count_filtered), stringsAsFactors=FALSE )
rownames(all_genes)<-all_genes$gene
N_expressed_genes<-length(all_genes$gene)

gene.length<-gene.length[gene.length$ensembl_gene_id %in% all_genes$gene,]

annotation.genelength.biomart_vector<-gene.length$transcript_length
names(annotation.genelength.biomart_vector)<-gene.length$ensembl_gene_id

annotation.GO.BP.biomart<-annotation.GO.biomart[annotation.GO.biomart$namespace_1003=="biological_process", c(1,3)]
annotation.GO.BP.biomart<-annotation.GO.BP.biomart[annotation.GO.BP.biomart$ensembl_gene_id %in% rownames(all_genes),]
annotation.GO.MF.biomart<-annotation.GO.biomart[annotation.GO.biomart$namespace_1003=="molecular_function", c(1,3)]
annotation.GO.MF.biomart<-annotation.GO.MF.biomart[annotation.GO.MF.biomart$ensembl_gene_id %in% rownames(all_genes),]
```

```
test.genes<-data.frame(a=unique(cumulus_gene_annotation[(cumulus_gene_annotation$FDR<0.01) & (cumulus_gene_annotation$padj<0.01) & (cumulus_gene_annotation$logFC<0),1] ), stringsAsFactors=FALSE)
all_genes_numeric<-as.integer(all_genes$gene %in%test.genes$a)
names(all_genes_numeric)<-all_genes$gene

N_sig_genes<-length(test.genes$a)

set.seed(9830)
pwf<-nullp(all_genes_numeric, bias.data=annotation.genelength.biomart_vector, plot.fit=FALSE ) 
GO_BP_Cats_cumulus_up_BCB_neg<-goseq(pwf,gene2cat=annotation.GO.BP.biomart, method ="Sampling", repcnt = 5000, use_genes_without_cat=FALSE)
GO_BP_Cats_cumulus_up_BCB_neg<-GO_BP_Cats_cumulus_up_BCB_neg[GO_BP_Cats_cumulus_up_BCB_neg$numDEInCat>3,]
GO_BP_Cats_cumulus_up_BCB_neg$FWER<-p.adjust(GO_BP_Cats_cumulus_up_BCB_neg$over_represented_pvalue, method ="holm")
GO_BP_Cats_cumulus_up_BCB_neg<-GO_BP_Cats_cumulus_up_BCB_neg[with(GO_BP_Cats_cumulus_up_BCB_neg, order(FWER,over_represented_pvalue, -numDEInCat)), ]
#head(GO_BP_Cats_cumulus_up_BCB_neg, n=20)

GO_BP_Cats_cumulus_up_BCB_neg$fold_enrichment<-(GO_BP_Cats_cumulus_up_BCB_neg$numDEInCat/N_sig_genes)/(GO_BP_Cats_cumulus_up_BCB_neg$numInCat/N_expressed_genes)
annotation.GO.BP.biomart_testgenes<-annotation.GO.BP.biomart[annotation.GO.BP.biomart$ensembl_gene_id %in% test.genes$a, ]
GO_BP_Cats_cumulus_up_BCB_neg<-merge(GO_BP_Cats_cumulus_up_BCB_neg,annotation.GO.BP.biomart_testgenes, by.x="category", by.y="go_id", all.x=TRUE, all.y=FALSE)
GO_BP_Cats_cumulus_up_BCB_neg<-merge(GO_BP_Cats_cumulus_up_BCB_neg, annotation.ensembl.symbol, by.x="ensembl_gene_id", by.y="ensembl_gene_id", all=FALSE, all.x=TRUE, all.y=FALSE)
GO_BP_Cats_cumulus_up_BCB_neg<-GO_BP_Cats_cumulus_up_BCB_neg[with(GO_BP_Cats_cumulus_up_BCB_neg, order(FWER,term)), ]
GO_BP_Cats_cumulus_up_BCB_neg<-GO_BP_Cats_cumulus_up_BCB_neg[GO_BP_Cats_cumulus_up_BCB_neg$FWER<0.05,]
#write.table(GO_BP_Cats_cumulus_up_BCB_neg, file= "/mnt/storage/lab_folder/shared_R_codes/fernando/BCB_oocyte_cumulus/results/2022_01_18_GO_BP_Cats_cumulus_up_BCB_neg_at_fdr01.txt", append = FALSE, quote = FALSE, sep = "\t" ,row.names = FALSE)


test.genes<-data.frame(a=unique(cumulus_gene_annotation[(cumulus_gene_annotation$FDR<0.01) & (cumulus_gene_annotation$padj<0.01) & (cumulus_gene_annotation$logFC>0),1] ), stringsAsFactors=FALSE)
all_genes_numeric<-as.integer(all_genes$gene %in%test.genes$a)
names(all_genes_numeric)<-all_genes$gene

N_sig_genes<-length(test.genes$a)

set.seed(9830)
pwf<-nullp(all_genes_numeric, bias.data=annotation.genelength.biomart_vector, plot.fit=FALSE ) 
GO_BP_Cats_cumulus_down_BCB_neg<-goseq(pwf,gene2cat=annotation.GO.BP.biomart, method ="Sampling", repcnt = 5000, use_genes_without_cat=FALSE)
GO_BP_Cats_cumulus_down_BCB_neg<-GO_BP_Cats_cumulus_down_BCB_neg[GO_BP_Cats_cumulus_down_BCB_neg$numDEInCat>3,]
GO_BP_Cats_cumulus_down_BCB_neg$FWER<-p.adjust(GO_BP_Cats_cumulus_down_BCB_neg$over_represented_pvalue, method ="holm")
GO_BP_Cats_cumulus_down_BCB_neg<-GO_BP_Cats_cumulus_down_BCB_neg[with(GO_BP_Cats_cumulus_down_BCB_neg, order(FWER,over_represented_pvalue, -numDEInCat)), ]
#head(GO_BP_Cats_cumulus_down_BCB_neg, n=20)

GO_BP_Cats_cumulus_down_BCB_neg$fold_enrichment<-(GO_BP_Cats_cumulus_down_BCB_neg$numDEInCat/N_sig_genes)/(GO_BP_Cats_cumulus_down_BCB_neg$numInCat/N_expressed_genes)
annotation.GO.BP.biomart_testgenes<-annotation.GO.BP.biomart[annotation.GO.BP.biomart$ensembl_gene_id %in% test.genes$a, ]
GO_BP_Cats_cumulus_down_BCB_neg<-merge(GO_BP_Cats_cumulus_down_BCB_neg,annotation.GO.BP.biomart_testgenes, by.x="category", by.y="go_id", all.x=TRUE, all.y=FALSE)
GO_BP_Cats_cumulus_down_BCB_neg<-merge(GO_BP_Cats_cumulus_down_BCB_neg, annotation.ensembl.symbol, by.x="ensembl_gene_id", by.y="ensembl_gene_id", all=FALSE, all.x=TRUE, all.y=FALSE)
GO_BP_Cats_cumulus_down_BCB_neg<-GO_BP_Cats_cumulus_down_BCB_neg[with(GO_BP_Cats_cumulus_down_BCB_neg, order(FWER,term)), ]
GO_BP_Cats_cumulus_down_BCB_neg<-GO_BP_Cats_cumulus_down_BCB_neg[GO_BP_Cats_cumulus_down_BCB_neg$FWER<0.05,]
#write.table(GO_BP_Cats_cumulus_down_BCB_neg, file= "/mnt/storage/lab_folder/shared_R_codes/fernando/BCB_oocyte_cumulus/results/2022_01_18_GO_BP_Cats_cumulus_down_BCB_neg_at_fdr01.txt", append = FALSE, quote = FALSE, sep = "\t" ,row.names = FALSE)
```

```
GO_plot<-ggplot(data=GO_BP_Cats_cumulus_up_BCB_neg, aes(x=term, y=-log10(FWER)))+
  geom_point(aes(color=fold_enrichment))+
  scale_y_continuous(name=bquote(-Log[10](FWER)), limits=c(1,2.5))+
  coord_flip()+
  scale_colour_gradient(name= "Fold enrichment",low = "#B8B8B8", high = "#080808", na.value = NA,limits = c(1, 75))+
  scale_x_discrete(name=NULL)+
  theme_classic() + 
  theme(
    legend.direction = "horizontal",
    legend.position = c(0.9,1),
    legend.key.size = unit(0.5, 'mm'),
    legend.text=element_text(size=5,margin = margin(t = 0, r = 0, b = 0, l = 0, unit = "cm")),
    legend.title=element_text(size=5),
    axis.text.x = element_text(color="black", size=9),
    axis.text.y = element_text(color="black", size=7),
    axis.title.y = element_blank(),
    axis.title.x = element_text(vjust=1,margin = margin(t = 0, r = 0, b = 0, l = 0, unit = "cm"), size = 10),
    plot.margin=unit(c(1,5,0,0), units="mm")
  ) + guides(color=guide_legend(title.position="top",title.hjust =0.5))
```

```
tpm_cumulus_bcb_pos<-cumulus_tpm[,grep("pos", cumulus_group)]
tpm_cumulus_bcb_neg<-cumulus_tpm[,grep("neg", cumulus_group)]

GO_BP_Cats_cumulus_up_BCB_neg_gene_exp<-GO_BP_Cats_cumulus_up_BCB_neg[GO_BP_Cats_cumulus_up_BCB_neg$term == "positive regulation of transcription by RNA polymerase II",]

GO_BP_Cats_cumulus_up_BCB_neg_gene_exp_1<-GO_BP_Cats_cumulus_up_BCB_neg[GO_BP_Cats_cumulus_up_BCB_neg$term == "regulation of transcription, DNA-templated",]

GO_BP_Cats_cumulus_up_BCB_neg_gene_exp<-GO_BP_Cats_cumulus_up_BCB_neg_gene_exp[GO_BP_Cats_cumulus_up_BCB_neg_gene_exp$ensembl_gene_id %in% intersect(GO_BP_Cats_cumulus_up_BCB_neg_gene_exp$ensembl_gene_id, GO_BP_Cats_cumulus_up_BCB_neg_gene_exp_1$ensembl_gene_id),]

data_cumulus_deg_chart_1<-data.frame( stringsAsFactors=FALSE)
data_cumulus_deg_chart_2<-data.frame( stringsAsFactors=FALSE)

for (i in  seq(dim(GO_BP_Cats_cumulus_up_BCB_neg_gene_exp)[1])){
  
  cumulus_gene_id<-GO_BP_Cats_cumulus_up_BCB_neg_gene_exp[i,1]
  cumulus_gene_symbol<-GO_BP_Cats_cumulus_up_BCB_neg_gene_exp$external_gene_name[i]
  if(cumulus_gene_symbol==""){GO_BP_Cats_cumulus_up_BCB_neg_gene_exp<-ensembl_gene_id}
  
  tpm_bcb_pos<-tpm_cumulus_bcb_pos[rownames(tpm_cumulus_bcb_pos)==cumulus_gene_id,]
  tpm_bcb_neg<-tpm_cumulus_bcb_neg[rownames(tpm_cumulus_bcb_neg)==cumulus_gene_id,]
  
  data_cumulus_deg_chart_1<-rbind(data.frame(t(tpm_bcb_pos)), data.frame(t(tpm_bcb_neg)))
  colnames(data_cumulus_deg_chart_1)<-"tpm"
  data_cumulus_deg_chart_1$group<-rep(c("BCBpos", "BCBneg"), c(9,10))
  data_cumulus_deg_chart_1$gene<-cumulus_gene_symbol
  data_cumulus_deg_chart_1$chart<-i
  data_cumulus_deg_chart_2<-rbind(data_cumulus_deg_chart_2,data_cumulus_deg_chart_1)
}

data_cumulus_deg_chart_2<-data_cumulus_deg_chart_2[data_cumulus_deg_chart_2$tpm >0,]
data_cumulus_deg_chart_2$group<-factor(data_cumulus_deg_chart_2$group, levels=c("BCBpos", "BCBneg"))

cumulus_deg_plots <- list()
k<-1
for (j in c(1:10)){
  data_cumulus_deg_chart_3<-data_cumulus_deg_chart_2[data_cumulus_deg_chart_2$chart == j , ]
  cumulus_deg_plot<-ggplot(data=data_cumulus_deg_chart_3, aes(x=group , y=tpm) )+
    stat_summary(fun = mean,colour = "gray", size = 0.2,  geom = "crossbar")+
    geom_jitter(position = position_jitter(width = .1, height=0), size=1, color="black",shape=21,aes(fill=as.factor(group)))+
    scale_x_discrete(name=NULL)+
    scale_fill_manual(name=NULL, values = c("#0072B2", "#ffffff"))+
    scale_y_continuous(name=NULL,breaks = function(x) unique(floor(pretty(seq(0, (max(x) + 1) * 1.1)))))+
    ggtitle(data_cumulus_deg_chart_3$gene[1])+
    #ggrepel::geom_text_repel(data = data_cumulus_deg_chart_3, aes(label = sample))+
    theme_bw()+
    theme(panel.grid= element_blank(),
          panel.background = element_blank(),
          panel.grid.minor = element_blank(), 
          panel.grid.major = element_blank(),
          plot.background = element_blank(),
          plot.margin=unit(c(0,1,3,0), units="mm"),
          strip.background = element_rect(fill = "white"),
          #strip.text.x = element_text(colour = 'black', face="italic",size = 9),
          #legend.text = element_text( colour = 'black', size = 10 ),
          axis.text.x = element_blank(),
          axis.text.y = element_text( colour = 'black', size = 8 ),
          #axis.title.x = element_text( colour = 'black', size = 9, face="italic" ),
          #axis.title.y = element_text( colour = 'black', size = 9 ),
          legend.key.size = unit(0.9,"cm"),
          legend.position="none",
          axis.ticks.x = element_blank(),
          plot.title = element_text(size = 7,face = "italic",margin=margin(0,0,0,0))
    ) 
  
  cumulus_deg_plots[[k]] <- cumulus_deg_plot
  k<-k+1
}
plot_1<-plot_grid(plotlist=cumulus_deg_plots,nrow=2)


data_cumulus_deg_chart_1<-data.frame( stringsAsFactors=FALSE)
data_cumulus_deg_chart_2<-data.frame( stringsAsFactors=FALSE)

for (i in  seq(dim(GO_BP_Cats_cumulus_down_BCB_neg)[1])){
  
  cumulus_gene_id<-GO_BP_Cats_cumulus_down_BCB_neg[i,1]
  cumulus_gene_symbol<-GO_BP_Cats_cumulus_down_BCB_neg$external_gene_name[i]
  if(cumulus_gene_symbol==""){GO_BP_Cats_cumulus_down_BCB_neg<-ensembl_gene_id}
  
  tpm_bcb_pos<-tpm_cumulus_bcb_pos[rownames(tpm_cumulus_bcb_pos)==cumulus_gene_id,]
  tpm_bcb_neg<-tpm_cumulus_bcb_neg[rownames(tpm_cumulus_bcb_neg)==cumulus_gene_id,]
  
  data_cumulus_deg_chart_1<-rbind(data.frame(t(tpm_bcb_pos)), data.frame(t(tpm_bcb_neg)))
  colnames(data_cumulus_deg_chart_1)<-"tpm"
  data_cumulus_deg_chart_1$group<-rep(c("BCBpos", "BCBneg"), c(9,10))
  data_cumulus_deg_chart_1$gene<-cumulus_gene_symbol
  data_cumulus_deg_chart_1$chart<-i
  data_cumulus_deg_chart_2<-rbind(data_cumulus_deg_chart_2,data_cumulus_deg_chart_1)
}

data_cumulus_deg_chart_2<-data_cumulus_deg_chart_2[data_cumulus_deg_chart_2$tpm >0,]
data_cumulus_deg_chart_2$group<-factor(data_cumulus_deg_chart_2$group, levels=c("BCBpos", "BCBneg"))

cumulus_deg_plots <- list()
k<-1
for (j in c(1:4)){
  data_cumulus_deg_chart_3<-data_cumulus_deg_chart_2[data_cumulus_deg_chart_2$chart == j , ]
  cumulus_deg_plot<-ggplot(data=data_cumulus_deg_chart_3, aes(x=group , y=tpm) )+
    stat_summary(fun = mean,colour = "gray", size = 0.2,  geom = "crossbar")+
    geom_jitter(position = position_jitter(width = .1, height=0), size=1, color="black",shape=21,aes(fill=as.factor(group)))+
    scale_x_discrete(name=NULL)+
    scale_fill_manual(name=NULL, values = c("#0072B2", "#ffffff"))+
    scale_y_continuous(name=NULL,breaks = function(x) unique(floor(pretty(seq(0, (max(x) + 1) * 1.1)))))+
    ggtitle(data_cumulus_deg_chart_3$gene[1])+
    #ggrepel::geom_text_repel(data = data_cumulus_deg_chart_3, aes(label = sample))+
    theme_bw()+
    theme(panel.grid= element_blank(),
          panel.background = element_blank(),
          panel.grid.minor = element_blank(), 
          panel.grid.major = element_blank(),
          plot.background = element_blank(),
          plot.margin=unit(c(0,1,0,0), units="mm"),
          strip.background = element_rect(fill = "white"),
          #strip.text.x = element_text(colour = 'black', face="italic",size = 9),
          #legend.text = element_text( colour = 'black', size = 10 ),
          axis.text.x = element_blank(),
          axis.text.y = element_text( colour = 'black', size = 8 ),
          #axis.title.x = element_text( colour = 'black', size = 9, face="italic" ),
          #axis.title.y = element_text( colour = 'black', size = 9 ),
          legend.key.size = unit(0.9,"cm"),
          legend.position="none",
          axis.ticks.x = element_blank(),
          plot.title = element_text(size = 7,face = "italic",margin=margin(0,0,0,0))
    ) 
  
  cumulus_deg_plots[[k]] <- cumulus_deg_plot
  k<-k+1
}
plot_2<-plot_grid(plotlist=cumulus_deg_plots,nrow=1)
```

##### Figure 3

```
plot_grid(plot_grid(volcano_plot_deg_cc,pca_plot_deg_cc, rel_widths=c(1,0.8), align="h", labels = c('A', 'B'),label_size = 12, label_fontface="plain"), plot_grid(GO_plot,plot_grid(plot_1,plot_grid(NULL,plot_2,rel_widths=c(0.25,1) , nrow=1, labels = c('', 'D'),label_size = 12, label_fontface="plain",hjust=2), nrow=2, rel_heights=c(1,0.5)), align="none",labels = c('C'),label_size = 12, label_fontface="plain"),nrow=2, rel_heights=c(1,1.4))
```

Figure 3. Differential transcript abundance in
cumulus cells. (A) Depiction of the genes with significant differences
in transcript abundance in cumulus cells surrounding oocytes categorized
by BCB staining. (B) PCA plot of the 172 differentially expressed genes
in cumulus cells collected from BCB positive or negative oocytes. (C)
Biological processes categories enriched in 122 with greater transcript
abundance in cumulus cells obtained from BCB negative oocytes relative
to their counterparts. (D) Four genes with greater abundance in cumulus
cells surrounding BCB positive oocytes and present in ‘transmembrane
transport’.

#### Differential gene coexpression between oocytes and cumulus cells

Calculate the gene co-expression between oocytes and
cumulus cells within each group.

```
asinh_transf_CTF_normalized_oocyte_pos<-asinh_transf_CTF_normalized_oocyte[grep("pos",oocyte_group)]

asinh_transf_CTF_normalized_cumulus_pos<-asinh_transf_CTF_normalized_cumulus[grep("pos",cumulus_group)]

cor_oocyte_BCB_pos<-corAndPvalue(t(asinh_transf_CTF_normalized_oocyte_pos),t(asinh_transf_CTF_normalized_cumulus_pos), use = "pairwise.complete.obs",alternative="two.sided")

cor_oocyte_BCB_pos_value<-reshape2::melt(cor_oocyte_BCB_pos$cor)
cor_oocyte_BCB_pos_value<-as.data.table(cor_oocyte_BCB_pos_value)
cor_oocyte_BCB_pos_p_value<-melt(cor_oocyte_BCB_pos$p)
cor_oocyte_BCB_pos_p_value<-as.data.table(cor_oocyte_BCB_pos_p_value)
cor_oocyte_BCB_pos<-cbind(cor_oocyte_BCB_pos_value,cor_oocyte_BCB_pos_p_value)
rm(cor_oocyte_BCB_pos_p_value,cor_oocyte_BCB_pos_value)
cor_oocyte_BCB_pos<-cor_oocyte_BCB_pos[,c(1,2,3,6)]
cor_oocyte_BCB_pos<-cor_oocyte_BCB_pos[order(cor_oocyte_BCB_pos$value),]
#head(cor_oocyte_BCB_pos)
colnames(cor_oocyte_BCB_pos)<-c("gene_oocyte","gene_cumulus","correlation_pos","p_pos")

asinh_transf_CTF_normalized_oocyte_neg<-asinh_transf_CTF_normalized_oocyte[grep("neg",oocyte_group)]
asinh_transf_CTF_normalized_cumulus_neg<-asinh_transf_CTF_normalized_cumulus[grep("neg",cumulus_group)]
cor_oocyte_BCB_neg<-corAndPvalue(t(asinh_transf_CTF_normalized_oocyte_neg),t(asinh_transf_CTF_normalized_cumulus_neg), use = "pairwise.complete.obs",alternative="two.sided")

cor_oocyte_BCB_neg_value<-reshape2::melt(cor_oocyte_BCB_neg$cor)
cor_oocyte_BCB_neg_value<-as.data.table(cor_oocyte_BCB_neg_value)
cor_oocyte_BCB_neg_p_value<-melt(cor_oocyte_BCB_neg$p)
cor_oocyte_BCB_neg_p_value<-as.data.table(cor_oocyte_BCB_neg_p_value)
cor_oocyte_BCB_neg<-cbind(cor_oocyte_BCB_neg_value,cor_oocyte_BCB_neg_p_value)
rm(cor_oocyte_BCB_neg_p_value,cor_oocyte_BCB_neg_value)
cor_oocyte_BCB_neg<-cor_oocyte_BCB_neg[,c(1,2,3,6)]
cor_oocyte_BCB_neg<-cor_oocyte_BCB_neg[order(cor_oocyte_BCB_neg$value),]
#head(cor_oocyte_BCB_neg)
colnames(cor_oocyte_BCB_neg)<-c("gene_oocyte","gene_cumulus","correlation_neg","p_neg")

cor_oocyte_BCB_pos_neg<-merge(cor_oocyte_BCB_pos, cor_oocyte_BCB_neg, by=c("gene_oocyte","gene_cumulus"), all=TRUE)

cor_oocyte_BCB_pos_neg<-cor_oocyte_BCB_pos_neg[order(cor_oocyte_BCB_pos_neg$p_pos),]
```

Estimate the empirical false discovery rate for
co-expression between BCB positive oocytes and cumulus cells.

```
setwd("/mnt/storage/lab_folder/shared_R_codes/fernando/BCB_oocyte_cumulus/results/")
asinh_transf_CTF_normalized_oocyte_pos_big_matrix<- as.big.matrix(t(asinh_transf_CTF_normalized_oocyte_pos) , type = "double", 
                    separated = FALSE, 
                    backingfile = "asinh_transf_CTF_normalized_oocyte_pos.bin", 
                    descriptorfile = "asinh_transf_CTF_normalized_oocyte_pos.desc", 
                    share=TRUE)
# get a description of the matrix
mdesc_oocyte <- describe(asinh_transf_CTF_normalized_oocyte_pos_big_matrix)

asinh_transf_CTF_normalized_cumulus_pos_big_matrix <- as.big.matrix(t(asinh_transf_CTF_normalized_cumulus_pos), type = "double", 
                      separated = FALSE, 
                      backingfile = "asinh_transf_CTF_normalized_cumulus_pos.bin", 
                      descriptorfile = "asinh_transf_CTF_normalized_cumulus_pos.desc",
                      share=TRUE)
# get a description of the matrix
mdesc_cumulus<- describe(asinh_transf_CTF_normalized_cumulus_pos_big_matrix)

permutation<-permutations(n = 9, r = 9, v = 1:9,repeats.allowed=FALSE)
permutation<-permutation[342880:362879,]

rand<-dim(permutation)[1]

sequence.correlation<-seq(0.90, 1, 0.01)

results <- filebacked.big.matrix(length(sequence.correlation),rand, type="double", init=0, separated=FALSE, 
                                 backingfile="incidence_matrix.bin",
                                 descriptor="incidence_matrix.desc")
mdesc_result<- describe(results)

cl <- makeCluster(30)
registerDoParallel(cl)

results[,]<-foreach(i = sequence.correlation, .combine='rbind', .inorder=TRUE,  .packages=c("WGCNA","reshape","bigmemory"), .noexport=c("eet", "endo"), .verbose=FALSE) %:%
  
  foreach(j = 1:rand, .combine='cbind', .inorder=FALSE,.packages=c("WGCNA","reshape","bigmemory"), .noexport=c("asinh_transf_CTF_normalized_oocyte_pos_big_matrix", "asinh_transf_CTF_normalized_cumulus_pos_big_matrix"), .verbose=FALSE ) %dopar% {
    
    require(bigmemory)
    
    oocyte<- attach.big.matrix("asinh_transf_CTF_normalized_oocyte_pos.desc")
    cumulus<- attach.big.matrix("asinh_transf_CTF_normalized_cumulus_pos.desc")
    random<-WGCNA::cor(oocyte[permutation[j,],],  cumulus[,],  use = "pairwise.complete.obs", method="pearson")
    
    length(which(abs(random) > i))  
    
  }

stopCluster(cl)

total.rand <- 20000 * 150076995

qvalue_BCB_pos<-data.frame(correlation = sequence.correlation, e.pvalue= (rowSums(results[,])+1)/(total.rand+1))

write.table(qvalue_BCB_pos, file = "qvalue_BCB_pos_2022_01_25.txt",quote = TRUE, sep = "\t",row.names = TRUE,col.names = TRUE)

system("rm asinh_transf_CTF_normalized_oocyte_pos.bin")
system("rm asinh_transf_CTF_normalized_oocyte_pos.desc")
system("rm asinh_transf_CTF_normalized_cumulus_pos.bin")
system("rm asinh_transf_CTF_normalized_cumulus_pos.desc")
system("rm incidence_matrix.bin")
system("rm incidence_matrix.desc")
```

Estimate the empirical false discovery rate for
co-expression between BCB negative oocytes and cumulus cells.

```
setwd("/mnt/storage/lab_folder/shared_R_codes/fernando/BCB_oocyte_cumulus/results/")
asinh_transf_CTF_normalized_oocyte_neg_big_matrix<- as.big.matrix(t(asinh_transf_CTF_normalized_oocyte_neg) , type = "double", 
                    separated = FALSE, 
                    backingfile = "asinh_transf_CTF_normalized_oocyte_neg.bin", 
                    descriptorfile = "asinh_transf_CTF_normalized_oocyte_neg.desc", 
                    share=TRUE)
# get a description of the matrix
mdesc_oocyte <- describe(asinh_transf_CTF_normalized_oocyte_neg_big_matrix)

asinh_transf_CTF_normalized_cumulus_neg_big_matrix <- as.big.matrix(t(asinh_transf_CTF_normalized_cumulus_neg), type = "double", 
                      separated = FALSE, 
                      backingfile = "asinh_transf_CTF_normalized_cumulus_neg.bin", 
                      descriptorfile = "asinh_transf_CTF_normalized_cumulus_neg.desc",
                      share=TRUE)
# get a description of the matrix
mdesc_cumulus<- describe(asinh_transf_CTF_normalized_cumulus_neg_big_matrix)

permutation<-permutations(n = 10, r = 10, v = 1:10,repeats.allowed=FALSE)
permutation<-permutation[500320:520320,]

rand<-dim(permutation)[1]

sequence.correlation<-seq(0.90, 1, 0.01)

results <- filebacked.big.matrix(length(sequence.correlation),rand, type="double", init=0, separated=FALSE, 
                                 backingfile="incidence_matrix.bin",
                                 descriptor="incidence_matrix.desc")
mdesc_result<- describe(results)

cl <- makeCluster(30)
registerDoParallel(cl)

results[,]<-foreach(i = sequence.correlation, .combine='rbind', .inorder=TRUE,  .packages=c("WGCNA","reshape","bigmemory"), .noexport=c("eet", "endo"), .verbose=FALSE) %:%
  
  foreach(j = 1:rand, .combine='cbind', .inorder=FALSE,.packages=c("WGCNA","reshape","bigmemory"), .noexport=c("asinh_transf_CTF_normalized_oocyte_neg_big_matrix", "asinh_transf_CTF_normalized_cumulus_neg_big_matrix"), .verbose=FALSE ) %dopar% {
    
    require(bigmemory)
    
    oocyte<- attach.big.matrix("asinh_transf_CTF_normalized_oocyte_neg.desc")
    cumulus<- attach.big.matrix("asinh_transf_CTF_normalized_cumulus_neg.desc")
    random<-WGCNA::cor(oocyte[permutation[j,],],  cumulus[,],  use = "pairwise.complete.obs", method="pearson")
    
    length(which(abs(random) > i))  
    
  }

stopCluster(cl)

total.rand <- 20000 * 150076995

qvalue_BCB_neg<-data.frame(correlation = sequence.correlation, e.pvalue= (rowSums(results[,])+1)/(total.rand+1))

write.table(qvalue_BCB_neg, file = "qvalue_BCB_neg_2022_01_18.txt",quote = TRUE, sep = "\t",row.names = TRUE,col.names = TRUE)

system("rm asinh_transf_CTF_normalized_oocyte_neg.bin")
system("rm asinh_transf_CTF_normalized_oocyte_neg.desc")
system("rm asinh_transf_CTF_normalized_cumulus_neg.bin")
system("rm asinh_transf_CTF_normalized_cumulus_neg.desc")
system("rm incidence_matrix.bin")
system("rm incidence_matrix.desc")
```

##### Supplemental figure 4

```
qvalue_BCB_pos<-read.table("/mnt/storage/lab_folder/shared_R_codes/fernando/BCB_oocyte_cumulus/results/qvalue_BCB_pos_2022_01_19.txt",stringsAsFactors=FALSE, header =TRUE, sep="\t")

qvalue_BCB_neg<-read.table("/mnt/storage/lab_folder/shared_R_codes/fernando/BCB_oocyte_cumulus/results/qvalue_BCB_neg_2022_01_18.txt",stringsAsFactors=FALSE, header =TRUE, sep="\t")

qvalue_BCB_pos$log_pvalue <- -log((qvalue_BCB_pos$e.pvalue),10)
qvalue_BCB_neg$log_pvalue <- -log((qvalue_BCB_neg$e.pvalue),10)

font_size=12
ggplot()+
  geom_point(data=qvalue_BCB_pos, aes(x=correlation , y=log_pvalue), color="black", fill="blue", size=4, shape=21)+
  geom_line(data=qvalue_BCB_pos, aes(x=correlation , y=log_pvalue), color="black", size=0.1,linetype=3)+
  
  geom_point(data=qvalue_BCB_neg, aes(x=correlation , y=log_pvalue), color="black", fill="white", size=4, shape=21)+
  geom_line(data=qvalue_BCB_neg, aes(x=correlation , y=log_pvalue), color="black", size=0.1,linetype=3)+
  
  geom_hline(yintercept=-log((0.0000005),10))+
  
  scale_x_continuous(name="Pearson's correlation", limits = c(0.9, 1), breaks=seq(0.9,1, 0.01))+
  scale_y_continuous( limits = c(3, 13), breaks=seq(03,13, 1))+
  labs(y=expression(paste(-Log [10] ,"(empirical FDR)", sep="")))+
  ggtitle("eFDR pair-wise gene correlations oocyte-cumulus")+
  theme_bw()+
  theme(panel.grid= element_blank(),
        panel.background = element_blank(),
        panel.grid.minor = element_blank(),
        panel.grid.major = element_line(color="lightgray"),
        plot.background = element_blank(),
        axis.title=element_text(color="black", size=font_size),
        axis.text=element_text(color="black", size=font_size),
        panel.spacing = unit(c(0.4,0.4,0.4,0.4),"cm"),
        plot.margin = unit(c(0.5,0.5,0.5,0.5),"cm"),
        legend.position="none",
        plot.title = element_text(lineheight=.8, hjust=0.5))
```

Supplemental figure 4. Null distribution of empirical
false discovery rate calculated for different values of correlation.
Blue and white circles indicate the values calculated for BCB positive
and negative cumulus oocyte complexes, respectively. Horizontal line
crosses y-axis at -Log10(0.0000005).

##### Supplemental table 10

```
cor_oocyte_BCB_pos_neg$oocyte_gene_symbol <- annotation.ensembl.symbol$external_gene_name[match(cor_oocyte_BCB_pos_neg$gene_oocyte,annotation.ensembl.symbol$ensembl_gene_id)]
cor_oocyte_BCB_pos_neg$cumulus_gene_symbol <- annotation.ensembl.symbol$external_gene_name[match(cor_oocyte_BCB_pos_neg$gene_cumulus,annotation.ensembl.symbol$ensembl_gene_id)]
cor_oocyte_BCB_pos_neg_099<-cor_oocyte_BCB_pos_neg[abs(correlation_pos)>0.99]
write.table(cor_oocyte_BCB_pos_neg_099, file= "/mnt/storage/lab_folder/shared_R_codes/fernando/BCB_oocyte_cumulus/results/cor_oocyte_BCB_pos_neg_099.txt", append = FALSE, quote = FALSE, sep = "\t" ,row.names = FALSE)
```

##### Supplemental table 11

```
cor_oocyte_BCB_pos_neg_098<-cor_oocyte_BCB_pos_neg[abs(correlation_neg)>0.98]
write.table(cor_oocyte_BCB_pos_neg_098, file= "/mnt/storage/lab_folder/shared_R_codes/fernando/BCB_oocyte_cumulus/results/cor_oocyte_BCB_pos_neg_098.txt", append = FALSE, quote = FALSE, sep = "\t" ,row.names = FALSE)
```

```
cor_oocyte_BCB_pos_neg$oocyte_gene_symbol <- annotation.ensembl.symbol$external_gene_name[match(cor_oocyte_BCB_pos_neg$gene_oocyte,annotation.ensembl.symbol$ensembl_gene_id)]
cor_oocyte_BCB_pos_neg$cumulus_gene_symbol <- annotation.ensembl.symbol$external_gene_name[match(cor_oocyte_BCB_pos_neg$gene_cumulus,annotation.ensembl.symbol$ensembl_gene_id)]
cor_oocyte_BCB_pos_neg_099<-cor_oocyte_BCB_pos_neg[abs(correlation_pos)>0.99]
write.table(cor_oocyte_BCB_pos_neg_099, file= "/mnt/storage/lab_folder/shared_R_codes/fernando/BCB_oocyte_cumulus/results/cor_oocyte_BCB_pos_neg_099.txt", append = FALSE, quote = FALSE, sep = "\t" ,row.names = FALSE)
cor_oocyte_BCB_pos_neg_098<-cor_oocyte_BCB_pos_neg[abs(correlation_neg)>0.98]
write.table(cor_oocyte_BCB_pos_neg_098, file= "/mnt/storage/lab_folder/shared_R_codes/fernando/BCB_oocyte_cumulus/results/cor_oocyte_BCB_pos_neg_098.txt", append = FALSE, quote = FALSE, sep = "\t" ,row.names = FALSE)
```

##### Supplemental table 12

GO oocyte genes

```
gene.length<-read.delim("/mnt/storage/lab_folder/shared_R_codes/fernando/BCB_oocyte_cumulus/resources/2021_12_31_gene.length.txt.bz2", header=TRUE, sep= "\t",row.names=1, stringsAsFactors = FALSE)

all_genes<-data.frame( gene=oocyte_gene_annotation$Row.names, stringsAsFactors=FALSE )
rownames(all_genes)<-all_genes$gene
N_expressed_genes<-length(all_genes$gene)

gene.length<-gene.length[gene.length$ensembl_gene_id %in% all_genes$gene,]

annotation.genelength.biomart_vector<-gene.length$transcript_length
names(annotation.genelength.biomart_vector)<-gene.length$ensembl_gene_id

annotation.GO.BP.biomart<-annotation.GO.biomart[annotation.GO.biomart$namespace_1003=="biological_process", c(1,3)]
annotation.GO.BP.biomart<-annotation.GO.BP.biomart[annotation.GO.BP.biomart$ensembl_gene_id %in% rownames(all_genes),]
annotation.GO.MF.biomart<-annotation.GO.biomart[annotation.GO.biomart$namespace_1003=="molecular_function", c(1,3)]
annotation.GO.MF.biomart<-annotation.GO.MF.biomart[annotation.GO.MF.biomart$ensembl_gene_id %in% rownames(all_genes),]
```

```
test.genes<-data.frame(a=unique(as.character(unique(cor_oocyte_BCB_pos[abs(correlation_pos) > 0.992 ]$gene_oocyte))), stringsAsFactors=FALSE)
all_genes_numeric<-as.integer(all_genes$gene %in%test.genes$a)
names(all_genes_numeric)<-all_genes$gene

N_sig_genes<-length(test.genes$a)

set.seed(87175)
pwf<-nullp(all_genes_numeric, bias.data=annotation.genelength.biomart_vector, plot.fit=FALSE ) 
GO_BP_Cats_oocyte_corr_cumulus<-goseq(pwf,gene2cat=annotation.GO.BP.biomart, method ="Sampling", repcnt = 5000, use_genes_without_cat=FALSE)
GO_BP_Cats_oocyte_corr_cumulus<-GO_BP_Cats_oocyte_corr_cumulus[GO_BP_Cats_oocyte_corr_cumulus$numDEInCat>3,]
GO_BP_Cats_oocyte_corr_cumulus$FWER<-p.adjust(GO_BP_Cats_oocyte_corr_cumulus$over_represented_pvalue, method ="holm")
GO_BP_Cats_oocyte_corr_cumulus<-GO_BP_Cats_oocyte_corr_cumulus[with(GO_BP_Cats_oocyte_corr_cumulus, order(FWER,over_represented_pvalue, -numDEInCat)), ]
#head(GO_BP_Cats_oocyte_corr_cumulus, n=20)

GO_BP_Cats_oocyte_corr_cumulus$fold_enrichment<-(GO_BP_Cats_oocyte_corr_cumulus$numDEInCat/N_sig_genes)/(GO_BP_Cats_oocyte_corr_cumulus$numInCat/N_expressed_genes)
annotation.GO.BP.biomart_testgenes<-annotation.GO.BP.biomart[annotation.GO.BP.biomart$ensembl_gene_id %in% test.genes$a, ]
GO_BP_Cats_oocyte_corr_cumulus<-merge(GO_BP_Cats_oocyte_corr_cumulus,annotation.GO.BP.biomart_testgenes, by.x="category", by.y="go_id", all.x=TRUE, all.y=FALSE)
GO_BP_Cats_oocyte_corr_cumulus<-merge(GO_BP_Cats_oocyte_corr_cumulus, annotation.ensembl.symbol, by.x="ensembl_gene_id", by.y="ensembl_gene_id", all=FALSE, all.x=TRUE, all.y=FALSE)
#GO_BP_Cats_oocyte_corr_cumulus<-GO_BP_Cats_oocyte_corr_cumulus[GO_BP_Cats_oocyte_corr_cumulus$FWER<0.1,]
GO_BP_Cats_oocyte_corr_cumulus<-GO_BP_Cats_oocyte_corr_cumulus[with(GO_BP_Cats_oocyte_corr_cumulus, order(FWER,term)), ]
GO_BP_Cats_oocyte_corr_cumulus$term<-factor(GO_BP_Cats_oocyte_corr_cumulus$term, levels=c(rev(unique(GO_BP_Cats_oocyte_corr_cumulus$term))))
#write.table(GO_BP_Cats_oocyte_corr_cumulus, file= "/mnt/storage/lab_folder/shared_R_codes/fernando/BCB_oocyte_cumulus/results/2022_02_22_GO_BP_Cats_subset_oocyte_corr_cumulus.txt", append = FALSE, quote = FALSE, sep = "\t" ,row.names = FALSE)
```

##### Supplemental table 13

GO cumulus genes

```
gene.length<-read.delim("/mnt/storage/lab_folder/shared_R_codes/fernando/BCB_oocyte_cumulus/resources/2021_12_31_gene.length.txt.bz2", header=TRUE, sep= "\t",row.names=1, stringsAsFactors = FALSE)

all_genes<-data.frame( gene=cumulus_gene_annotation$Row.names, stringsAsFactors=FALSE )
rownames(all_genes)<-all_genes$gene
N_expressed_genes<-length(all_genes$gene)

gene.length<-gene.length[gene.length$ensembl_gene_id %in% all_genes$gene,]

annotation.genelength.biomart_vector<-gene.length$transcript_length
names(annotation.genelength.biomart_vector)<-gene.length$ensembl_gene_id

annotation.GO.BP.biomart<-annotation.GO.biomart[annotation.GO.biomart$namespace_1003=="biological_process", c(1,3)]
annotation.GO.BP.biomart<-annotation.GO.BP.biomart[annotation.GO.BP.biomart$ensembl_gene_id %in% rownames(all_genes),]
annotation.GO.MF.biomart<-annotation.GO.biomart[annotation.GO.biomart$namespace_1003=="molecular_function", c(1,3)]
annotation.GO.MF.biomart<-annotation.GO.MF.biomart[annotation.GO.MF.biomart$ensembl_gene_id %in% rownames(all_genes),]
```

```
test.genes<-data.frame(a=as.character(unique(cor_oocyte_BCB_pos[abs(correlation_pos) > 0.992 ]$gene_cumulus)), stringsAsFactors=FALSE)
all_genes_numeric<-as.integer(all_genes$gene %in%test.genes$a)
names(all_genes_numeric)<-all_genes$gene

N_sig_genes<-length(test.genes$a)

set.seed(88972)
pwf<-nullp(all_genes_numeric, bias.data=annotation.genelength.biomart_vector, plot.fit=FALSE ) 
GO_BP_Cats_cumulus_corr_oocyte<-goseq(pwf,gene2cat=annotation.GO.BP.biomart, method ="Sampling", repcnt = 5000, use_genes_without_cat=FALSE)
#GO_BP_Cats_cumulus_corr_oocyte<-goseq(pwf,gene2cat=annotation.GO.BP.biomart, method ="Wallenius", use_genes_without_cat=FALSE)
GO_BP_Cats_cumulus_corr_oocyte<-GO_BP_Cats_cumulus_corr_oocyte[GO_BP_Cats_cumulus_corr_oocyte$numDEInCat>3,]
GO_BP_Cats_cumulus_corr_oocyte$FWER<-p.adjust(GO_BP_Cats_cumulus_corr_oocyte$over_represented_pvalue, method ="holm")
GO_BP_Cats_cumulus_corr_oocyte<-GO_BP_Cats_cumulus_corr_oocyte[with(GO_BP_Cats_cumulus_corr_oocyte, order(FWER,over_represented_pvalue, -numDEInCat)), ]
#head(GO_BP_Cats_cumulus_corr_oocyte, n=20)

GO_BP_Cats_cumulus_corr_oocyte$fold_enrichment<-(GO_BP_Cats_cumulus_corr_oocyte$numDEInCat/N_sig_genes)/(GO_BP_Cats_cumulus_corr_oocyte$numInCat/N_expressed_genes)
annotation.GO.BP.biomart_testgenes<-annotation.GO.BP.biomart[annotation.GO.BP.biomart$ensembl_gene_id %in% test.genes$a, ]
GO_BP_Cats_cumulus_corr_oocyte<-merge(GO_BP_Cats_cumulus_corr_oocyte,annotation.GO.BP.biomart_testgenes, by.x="category", by.y="go_id", all.x=TRUE, all.y=FALSE)
GO_BP_Cats_cumulus_corr_oocyte<-merge(GO_BP_Cats_cumulus_corr_oocyte, annotation.ensembl.symbol, by.x="ensembl_gene_id", by.y="ensembl_gene_id", all=FALSE, all.x=TRUE, all.y=FALSE)
GO_BP_Cats_cumulus_corr_oocyte<-GO_BP_Cats_cumulus_corr_oocyte[with(GO_BP_Cats_cumulus_corr_oocyte, order(FWER,term)), ]
#GO_BP_Cats_cumulus_corr_oocyte<-GO_BP_Cats_cumulus_corr_oocyte[GO_BP_Cats_cumulus_corr_oocyte$FWER <= 0.1,]
GO_BP_Cats_cumulus_corr_oocyte$term<-factor(GO_BP_Cats_cumulus_corr_oocyte$term, levels=c(rev(unique(GO_BP_Cats_cumulus_corr_oocyte$term))))
#write.table(GO_BP_Cats_cumulus_corr_oocyte, file= "/mnt/storage/lab_folder/shared_R_codes/fernando/BCB_oocyte_cumulus/results/2022_02_22_GO_BP_Cats_subset_cumulus_corr_oocyte.txt", append = FALSE, quote = FALSE, sep = "\t" ,row.names = FALSE)
```

##### Figure 4

```
cor_oocyte_BCB_pos_neg_subset<- cor_oocyte_BCB_pos_neg[abs(correlation_pos)>0.99]

plot_coexpression_positive<-ggplot(aes(correlation_neg,correlation_pos), data=cor_oocyte_BCB_pos_neg_subset)+
geom_point(size=0.5)+
scale_y_continuous(name="co-expression \n cumulus oocyte BCB+")+
scale_x_continuous(name="cumulus oocyte BCB-")+
theme_classic(base_size=12)+
theme(
axis.title=element_text(color="black", size=10),
axis.text=element_text(color="black",  size=10),
)

cor_oocyte_BCB_pos_neg_subset$oocyte_gene_symbol <- annotation.ensembl.symbol$external_gene_name[match(cor_oocyte_BCB_pos_neg_subset$gene_oocyte,annotation.ensembl.symbol$ensembl_gene_id)]
cor_oocyte_BCB_pos_neg_subset$cumulus_gene_symbol <- annotation.ensembl.symbol$external_gene_name[match(cor_oocyte_BCB_pos_neg_subset$gene_cumulus,annotation.ensembl.symbol$ensembl_gene_id)]

cor_oocyte_BCB_pos_neg_subset$angle<-NA
cor_oocyte_BCB_pos_neg_subset$r.squared<-NA
cor_oocyte_BCB_pos_neg_subset$rmse<-NA
cor_oocyte_BCB_pos_neg_subset$fstatistic<-NA

for (i in seq(dim(cor_oocyte_BCB_pos_neg_subset)[1]))
{
gene_oocyte<-as.character(cor_oocyte_BCB_pos_neg_subset$gene_oocyte)[i]
gene_cumulus<-as.character(cor_oocyte_BCB_pos_neg_subset$gene_cumulus)[i]

gene_symbol_oocyte<-as.character(cor_oocyte_BCB_pos_neg_subset$oocyte_gene_symbol)[i]
gene_symbol_cumulus<-as.character(cor_oocyte_BCB_pos_neg_subset$cumulus_gene_symbol)[i]

expression_oocyte<-data.frame(expression_oocyte=t(oocyte_tpm_filtered[rownames(oocyte_tpm_filtered)==gene_oocyte,grep("pos",oocyte_group)])[,1])
expression_cumulus<-data.frame( expression_cumulus=t(cumulus_tpm_filtered[rownames(cumulus_tpm_filtered)==gene_cumulus,grep("pos",cumulus_group)])[,1])

model_oocyte_cc<-lm(expression_oocyte$expression_oocyte ~ expression_cumulus$expression_cumulus)

cor_oocyte_BCB_pos_neg_subset$angle[i]<-unname(atan(coef(model_oocyte_cc)[2]) * (180 / pi))
cor_oocyte_BCB_pos_neg_subset$r.squared[i]<-summary(model_oocyte_cc)$adj.r.squared
cor_oocyte_BCB_pos_neg_subset$rmse[i]<-sqrt(mean(model_oocyte_cc$residuals^2))
cor_oocyte_BCB_pos_neg_subset$fstatistic[i]<-unname(summary(model_oocyte_cc)$fstatistic[1])
}

cor_oocyte_BCB_pos_neg_subset<-cor_oocyte_BCB_pos_neg_subset[!(cor_oocyte_BCB_pos_neg_subset$oocyte_gene_symbol=="") & !(cor_oocyte_BCB_pos_neg_subset$cumulus_gene_symbol=="" )]
cor_oocyte_BCB_pos_neg_subset<-cor_oocyte_BCB_pos_neg_subset[cor_oocyte_BCB_pos_neg_subset$angle > 35 & cor_oocyte_BCB_pos_neg_subset$angle < 60,]
cor_oocyte_BCB_pos_neg_subset<-cor_oocyte_BCB_pos_neg_subset[order(cor_oocyte_BCB_pos_neg_subset$rmse , decreasing = FALSE),]

dataframegraph<-data.frame()

for (i in c(1:4)){

gene_oocyte<-as.character(cor_oocyte_BCB_pos_neg_subset$gene_oocyte)[i]
gene_cumulus<-as.character(cor_oocyte_BCB_pos_neg_subset$gene_cumulus)[i]

gene_symbol_oocyte<-as.character(cor_oocyte_BCB_pos_neg_subset$oocyte_gene_symbol)[i]
gene_symbol_cumulus<-as.character(cor_oocyte_BCB_pos_neg_subset$cumulus_gene_symbol)[i]

expression_oocyte<-data.frame(expression_oocyte=t(oocyte_tpm_filtered[rownames(oocyte_tpm_filtered)==gene_oocyte,grep("pos",oocyte_group)])[,1])
expression_cumulus<-data.frame( expression_cumulus=t(cumulus_tpm_filtered[rownames(cumulus_tpm_filtered)==gene_cumulus,grep("pos",cumulus_group)])[,1])

graph<-i

dataframegraph<-rbind(data.frame(gene_oocyte,gene_cumulus,gene_symbol_oocyte,gene_symbol_cumulus,expression_oocyte,expression_cumulus,graph),dataframegraph)
}

plots<-list()

for (i in c(1:4)){

plots[[i]]<- ggplot(dataframegraph[dataframegraph$graph==i,], aes(x=expression_oocyte, y= expression_cumulus ))+
geom_smooth(method='lm', formula= y~x, size=0.5)+
geom_point( size=0.5)+
scale_x_continuous(name=dataframegraph[dataframegraph$graph==i,]$gene_symbol_oocyte[1],breaks = function(x) unique(floor(pretty(seq(0, (max(x) + 1) * 1.1)))))+
scale_y_continuous(name=dataframegraph[dataframegraph$graph==i,]$gene_symbol_cumulus[1], breaks = function(x) unique(floor(pretty(seq(0, (max(x) + 1) * 1.1)))))+
theme_classic()+
theme(
axis.title=element_text(face="italic", size=7),
axis.text=element_text( color="black",size=7),
)
}

top.grob <- textGrob("BCB +", gp=gpar(fontface="plain", col="black", fontsize=10))

plot_correlation_bcb_pos<-plot_grid(  grid.arrange(arrangeGrob(plot_grid(plotlist=plots, nrow=1), top = top.grob)))

dataframegraph<-data.frame()

for (i in c(1:4)){

gene_oocyte<-as.character(cor_oocyte_BCB_pos_neg_subset$gene_oocyte)[i]
gene_cumulus<-as.character(cor_oocyte_BCB_pos_neg_subset$gene_cumulus)[i]

gene_symbol_oocyte<-as.character(cor_oocyte_BCB_pos_neg_subset$oocyte_gene_symbol)[i]
gene_symbol_cumulus<-as.character(cor_oocyte_BCB_pos_neg_subset$cumulus_gene_symbol)[i]

expression_oocyte<-data.frame(expression_oocyte=t(oocyte_tpm_filtered[rownames(oocyte_tpm_filtered)==gene_oocyte,grep("neg",oocyte_group)])[,1])
expression_cumulus<-data.frame( expression_cumulus=t(cumulus_tpm_filtered[rownames(cumulus_tpm_filtered)==gene_cumulus,grep("neg",cumulus_group)])[,1])

graph<-i

dataframegraph<-rbind(data.frame(gene_oocyte,gene_cumulus,gene_symbol_oocyte,gene_symbol_cumulus,expression_oocyte,expression_cumulus,graph),dataframegraph)
}

plots<-list()

for (i in c(1:4)){

plots[[i]]<- ggplot(dataframegraph[dataframegraph$graph==i,], aes(x=expression_oocyte, y= expression_cumulus ))+
geom_smooth(method='lm', formula= y~x, size=0.5)+
geom_point( size=0.5)+
scale_x_continuous(name=dataframegraph[dataframegraph$graph==i,]$gene_symbol_oocyte[1],breaks = function(x) unique(floor(pretty(seq(0, (max(x) + 1) * 1.1)))))+
scale_y_continuous(name=dataframegraph[dataframegraph$graph==i,]$gene_symbol_cumulus[1], breaks = function(x) unique(floor(pretty(seq(0, (max(x) + 1) * 1.1)))))+
theme_classic()+
theme(
axis.title=element_text(face="italic", size=7),
axis.text=element_text( color="black",size=7),
)
}

top.grob <- textGrob("BCB -", gp=gpar(fontface="plain", col="black", fontsize=10))

plot_bcb_neg<-plot_grid(  grid.arrange(arrangeGrob(plot_grid(plotlist=plots, nrow=1), top = top.grob)))

y.grob <- textGrob("Cumulus cells", gp=gpar(fontface="plain", col="black", fontsize=10), rot=90)

x.grob <- textGrob("Oocytes", gp=gpar(fontface="plain", col="black", fontsize=10))

plot_bcb_pos_scatter_examples<-grid.arrange(arrangeGrob(plot_grid(plot_correlation_bcb_pos,plot_bcb_neg, nrow=2), left = y.grob,bottom = x.grob))

plot_a<-cowplot::plot_grid(plot_coexpression_positive,
                          plot_bcb_pos_scatter_examples, nrow=2, labels = c("A"), label_fontface = "plain", label_size = 12, rel_heights=c(0.8,1))


cor_oocyte_BCB_pos_neg_subset<- cor_oocyte_BCB_pos_neg[abs(correlation_neg)>0.98]

plot_coexpression_negative<-ggplot(aes(x=correlation_pos, y=correlation_neg), data=cor_oocyte_BCB_pos_neg_subset)+
geom_point(size=0.5)+
scale_y_continuous(name="co-expression \n cumulus oocyte BCB-")+
scale_x_continuous(name="cumulus oocyte BCB+")+
theme_classic(base_size=12)+
theme(
axis.title=element_text(color="black", size=10),
axis.text=element_text(color="black",  size=10),
)

cor_oocyte_BCB_pos_neg_subset$oocyte_gene_symbol <- annotation.ensembl.symbol$external_gene_name[match(cor_oocyte_BCB_pos_neg_subset$gene_oocyte,annotation.ensembl.symbol$ensembl_gene_id)]
cor_oocyte_BCB_pos_neg_subset$cumulus_gene_symbol <- annotation.ensembl.symbol$external_gene_name[match(cor_oocyte_BCB_pos_neg_subset$gene_cumulus,annotation.ensembl.symbol$ensembl_gene_id)]

cor_oocyte_BCB_pos_neg_subset$angle<-NA
cor_oocyte_BCB_pos_neg_subset$r.squared<-NA
cor_oocyte_BCB_pos_neg_subset$rmse<-NA
cor_oocyte_BCB_pos_neg_subset$fstatistic<-NA

for (i in seq(dim(cor_oocyte_BCB_pos_neg_subset)[1]))
{
gene_oocyte<-as.character(cor_oocyte_BCB_pos_neg_subset$gene_oocyte)[i]
gene_cumulus<-as.character(cor_oocyte_BCB_pos_neg_subset$gene_cumulus)[i]

gene_symbol_oocyte<-as.character(cor_oocyte_BCB_pos_neg_subset$oocyte_gene_symbol)[i]
gene_symbol_cumulus<-as.character(cor_oocyte_BCB_pos_neg_subset$cumulus_gene_symbol)[i]

expression_oocyte<-data.frame(expression_oocyte=t(oocyte_tpm_filtered[rownames(oocyte_tpm_filtered)==gene_oocyte,grep("neg",oocyte_group)])[,1])
expression_cumulus<-data.frame( expression_cumulus=t(cumulus_tpm_filtered[rownames(cumulus_tpm_filtered)==gene_cumulus,grep("neg",cumulus_group)])[,1])

model_oocyte_cc<-lm(expression_oocyte$expression_oocyte ~ expression_cumulus$expression_cumulus)

cor_oocyte_BCB_pos_neg_subset$angle[i]<-unname(atan(coef(model_oocyte_cc)[2]) * (180 / pi))
cor_oocyte_BCB_pos_neg_subset$r.squared[i]<-summary(model_oocyte_cc)$adj.r.squared
cor_oocyte_BCB_pos_neg_subset$rmse[i]<-sqrt(mean(model_oocyte_cc$residuals^2))
cor_oocyte_BCB_pos_neg_subset$fstatistic[i]<-unname(summary(model_oocyte_cc)$fstatistic[1])
}
cor_oocyte_BCB_pos_neg_subset<-cor_oocyte_BCB_pos_neg_subset[!(cor_oocyte_BCB_pos_neg_subset$oocyte_gene_symbol=="") & !(cor_oocyte_BCB_pos_neg_subset$cumulus_gene_symbol=="" )]
cor_oocyte_BCB_pos_neg_subset<-cor_oocyte_BCB_pos_neg_subset[cor_oocyte_BCB_pos_neg_subset$angle > 35 & cor_oocyte_BCB_pos_neg_subset$angle < 60,]
cor_oocyte_BCB_pos_neg_subset<-cor_oocyte_BCB_pos_neg_subset[order(cor_oocyte_BCB_pos_neg_subset$rmse , decreasing = FALSE),]

dataframegraph<-data.frame()

for (i in c(1:4)){

gene_oocyte<-as.character(cor_oocyte_BCB_pos_neg_subset$gene_oocyte)[i]
gene_cumulus<-as.character(cor_oocyte_BCB_pos_neg_subset$gene_cumulus)[i]

gene_symbol_oocyte<-as.character(cor_oocyte_BCB_pos_neg_subset$oocyte_gene_symbol)[i]
gene_symbol_cumulus<-as.character(cor_oocyte_BCB_pos_neg_subset$cumulus_gene_symbol)[i]

expression_oocyte<-data.frame(expression_oocyte=t(oocyte_tpm_filtered[rownames(oocyte_tpm_filtered)==gene_oocyte,grep("neg",oocyte_group)])[,1])
expression_cumulus<-data.frame( expression_cumulus=t(cumulus_tpm_filtered[rownames(cumulus_tpm_filtered)==gene_cumulus,grep("neg",cumulus_group)])[,1])

graph<-i

dataframegraph<-rbind(data.frame(gene_oocyte,gene_cumulus,gene_symbol_oocyte,gene_symbol_cumulus,expression_oocyte,expression_cumulus,graph),dataframegraph)
}

plots<-list()

for (i in c(1:4)){

plots[[i]]<- ggplot(dataframegraph[dataframegraph$graph==i,], aes(x=expression_oocyte, y= expression_cumulus ))+
geom_smooth(method='lm', formula= y~x, size=0.5)+
geom_point( size=0.5)+
scale_x_continuous(name=dataframegraph[dataframegraph$graph==i,]$gene_symbol_oocyte[1],breaks = function(x) unique(floor(pretty(seq(0, (max(x) + 1) * 1.1)))))+
scale_y_continuous(name=dataframegraph[dataframegraph$graph==i,]$gene_symbol_cumulus[1], breaks = function(x) unique(floor(pretty(seq(0, (max(x) + 1) * 1.1)))))+
theme_classic()+
theme(
axis.title=element_text(face="italic", size=7),
axis.text=element_text( color="black",size=7),
)
}

top.grob <- textGrob("BCB -", gp=gpar(fontface="plain", col="black", fontsize=10))

plot_correlation_bcb_neg<-plot_grid(  grid.arrange(arrangeGrob(plot_grid(plotlist=plots, nrow=1), top = top.grob)))

dataframegraph<-data.frame()

for (i in c(1:4)){

gene_oocyte<-as.character(cor_oocyte_BCB_pos_neg_subset$gene_oocyte)[i]
gene_cumulus<-as.character(cor_oocyte_BCB_pos_neg_subset$gene_cumulus)[i]

gene_symbol_oocyte<-as.character(cor_oocyte_BCB_pos_neg_subset$oocyte_gene_symbol)[i]
gene_symbol_cumulus<-as.character(cor_oocyte_BCB_pos_neg_subset$cumulus_gene_symbol)[i]

expression_oocyte<-data.frame(expression_oocyte=t(oocyte_tpm_filtered[rownames(oocyte_tpm_filtered)==gene_oocyte,grep("pos",oocyte_group)])[,1])
expression_cumulus<-data.frame( expression_cumulus=t(cumulus_tpm_filtered[rownames(cumulus_tpm_filtered)==gene_cumulus,grep("pos",cumulus_group)])[,1])

graph<-i

dataframegraph<-rbind(data.frame(gene_oocyte,gene_cumulus,gene_symbol_oocyte,gene_symbol_cumulus,expression_oocyte,expression_cumulus,graph),dataframegraph)
}

plots<-list()

for (i in c(1:4)){

plots[[i]]<- ggplot(dataframegraph[dataframegraph$graph==i,], aes(x=expression_oocyte, y= expression_cumulus ))+
geom_smooth(method='lm', formula= y~x, size=0.5)+
geom_point( size=0.5)+
scale_x_continuous(name=dataframegraph[dataframegraph$graph==i,]$gene_symbol_oocyte[1],breaks = function(x) unique(floor(pretty(seq(0, (max(x) + 1) * 1.1)))))+
scale_y_continuous(name=dataframegraph[dataframegraph$graph==i,]$gene_symbol_cumulus[1], breaks = function(x) unique(floor(pretty(seq(0, (max(x) + 1) * 1.1)))))+
theme_classic()+
theme(
axis.title=element_text(face="italic", size=7),
axis.text=element_text( color="black",size=7),
)
}

top.grob <- textGrob("BCB +", gp=gpar(fontface="plain", col="black", fontsize=10))

plot_bcb_pos<-plot_grid(  grid.arrange(arrangeGrob(plot_grid(plotlist=plots, nrow=1), top = top.grob)))

y.grob <- textGrob("Cumulus cells", gp=gpar(fontface="plain", col="black", fontsize=10), rot=90)

x.grob <- textGrob("Oocytes", gp=gpar(fontface="plain", col="black", fontsize=10))


plot_bcb_neg_scatter_examples<-cowplot::plot_grid(grid.arrange(arrangeGrob(plot_grid(plot_correlation_bcb_neg,plot_bcb_pos, nrow=2), left = y.grob,bottom = x.grob)))

plot_b<-cowplot::plot_grid(plot_coexpression_negative,
                            plot_bcb_neg_scatter_examples,
                             nrow=2, labels = c("B"), label_fontface = "plain", label_size = 12, rel_heights = c(0.8,1))


treshold<-0.992

asinh_transf_CTF_normalized_oocyte_pos_subset<-asinh_transf_CTF_normalized_oocyte_pos[rownames(asinh_transf_CTF_normalized_oocyte_pos) %in%  as.character(unique(cor_oocyte_BCB_pos[abs(correlation_pos) >= treshold ]$gene_oocyte)), ]
asinh_transf_CTF_normalized_cumulus_pos_subset<-asinh_transf_CTF_normalized_cumulus_pos[rownames(asinh_transf_CTF_normalized_cumulus_pos) %in%  as.character(unique(cor_oocyte_BCB_pos[abs(correlation_pos) >= treshold ]$gene_cumulus)), ]
#colnames(asinh_transf_CTF_normalized_oocyte_pos_subset)
colnames(asinh_transf_CTF_normalized_oocyte_pos_subset)<-c("coc17", "coc19", "coc24" ,"coc69", "coc70", "coc71" ,"coc72", "coc73", "coc74")
#colnames(asinh_transf_CTF_normalized_cumulus_pos_subset)
colnames(asinh_transf_CTF_normalized_cumulus_pos_subset)<-c("coc17", "coc19", "coc24" ,"coc69", "coc70", "coc71" ,"coc72", "coc73", "coc74")

source("/mnt/storage/lab_folder/shared_R_codes/fernando/BCB_oocyte_cumulus/resources/functions_plot_tanglegram.R")

hc1     <- t(asinh_transf_CTF_normalized_oocyte_pos_subset)  %>% dist %>%  flashClust(method = "average")
hc2     <- t(asinh_transf_CTF_normalized_cumulus_pos_subset) %>% dist %>%  flashClust(method = "average")
hcdata1 <- dendro_data_k(hc1,1)
hcdata2 <- dendro_data_k(hc2,1)

p1 <- plot_ggdendro(hcdata1,
                    direction   = "lr",
                    scale.color = "black",
                    branch.size = 0.5,
                    expand.y    = 1,
                    label.size  = 4)  +
                    theme(plot.background=element_blank(),
                    panel.background=element_blank(),
                    axis.text=element_blank(),
                    axis.title=element_blank(),
                    axis.ticks=element_blank())

p2 <- plot_ggdendro(hcdata2,
                    direction   = "rl",
                    scale.color = "black",
                    branch.size = 0.5,
                    expand.y    = 1,
                    label.size  = 4)+
                    theme(plot.background=element_blank(),
                    panel.background=element_blank(),
                    axis.text=element_blank(),
                    axis.title=element_blank(),
                    axis.ticks=element_blank())

idx <- data.frame(y1 = 1:nrow(hcdata1$labels),
                  y2 = match(hcdata1$labels$label, hcdata2$labels$label))

p3 <- ggplot() +
  geom_segment(data     = idx, 
               aes(x    = 0,
                   y    = y1,
                   xend = 1,
                   yend = y2),
               color    = c("blue", "blue", "blue","blue", "gray","gray","blue", "blue", "blue")) +
  theme_void()


plot_dendrogram_BCB_pos<-grid.arrange(arrangeGrob(p1, p3, p2, ncol = 3, widths = c(2, 0.3, 2), 
left = textGrob("oocyte", gp=gpar(fontface="plain", col="black", fontsize=font_size), rot=90), 
right = textGrob("cumulus", gp=gpar(fontface="plain", col="black", fontsize=font_size), rot=90),
top  = textGrob("BCB positive", gp=gpar(fontface="plain", col="black", fontsize=font_size))))
```

```
cor_oocyte_BCB_pos_subset<-cor_oocyte_BCB_pos[abs(correlation_pos) >= 0.992 ]

cor_oocyte_BCB_pos_subset$oocyte_symbol<- annotation.ensembl.symbol$external_gene_name[match(cor_oocyte_BCB_pos_subset$gene_oocyte,annotation.ensembl.symbol$ensembl_gene_id)]
cor_oocyte_BCB_pos_subset$cumulus_symbol<- annotation.ensembl.symbol$external_gene_name[match(cor_oocyte_BCB_pos_subset$gene_cumulus,annotation.ensembl.symbol$ensembl_gene_id)]

cor_oocyte_BCB_pos_subset<-cor_oocyte_BCB_pos_subset[!(cor_oocyte_BCB_pos_subset$oocyte_symbol =="") &  !(cor_oocyte_BCB_pos_subset$cumulus_symbol ==""),]

#write.table(cor_oocyte_BCB_pos_subset, file= "/mnt/storage/lab_folder/shared_R_codes/fernando/BCB_oocyte_cumulus/results/2022_02_24_cor_oocyte_BCB_pos_subset.txt", append = FALSE, quote = FALSE, sep = "\t" ,row.names = FALSE)

links<-data.frame(source=cor_oocyte_BCB_pos_subset$oocyte_symbol, target=cor_oocyte_BCB_pos_subset$cumulus_symbol)

links<-links[complete.cases(links),]
links<-links[!duplicated(links),]

nodes <- data.frame(name = unique(c(links$source, links$target)))
nodes$target<-nodes$name %in%cor_oocyte_BCB_pos_subset$cumulus_symbol
nodes$source<-nodes$name %in%cor_oocyte_BCB_pos_subset$oocyte_symbol


links$source <- match(links$source, nodes$name) - 1
links$target <- match(links$target, nodes$name) - 1
links$value <- 1

sn<-sankeyNetwork(Links = links, Nodes = nodes, Source = 'source',
                    Target = 'target',Value = 'value', NodeID = 'name',sinksRight=FALSE,  nodePadding = 8, fontSize = 10, margin=1500)
sn$x$nodes$target <- nodes$target
sn$x$nodes$source <- nodes$source

sn <- onRender(sn,
  '
  function(el) {
    d3.select(el)
      .selectAll(".node text")
      .filter(d => d.source)
      .attr("x", -3)
      .attr("text-anchor", "end");
  }
  '
)

setwd("/mnt/storage/lab_folder/shared_R_codes/fernando/BCB_oocyte_cumulus/results/")
saveNetwork(sn, "sn.html")
```

```
y.grob <- textGrob("Genes expressed in oocytes", gp=gpar(fontface="plain", col="black", fontsize=10), rot=90)
x.grob <- textGrob("Genes expressed in cumulus cells", gp=gpar(fontface="plain", col="black", fontsize=10),rot=90)
fig <- ggdraw() + draw_image(magick::image_read_pdf("/mnt/storage/lab_folder/shared_R_codes/fernando/BCB_oocyte_cumulus/results/sankeyNetwork.pdf", density = 600))
```

```
cowplot::plot_grid(
cowplot::plot_grid(plot_a,plot_b,ncol=1), 
cowplot::plot_grid( plot_dendrogram_BCB_pos, 
  cowplot::plot_grid(NULL, y.grob, cowplot::plot_grid(fig) ,x.grob, NULL, nrow=1, rel_widths=c(0.1,0.2,1,0.2,0.1)), 
  rel_heights=c(0.3,1),rel_widths=c(0.2,1),ncol=1, labels = c("C", "D"),hjust=-1 ,  label_fontface = "plain", label_size = 12)
, ncol=2
)
```

Figure 4. Differential co-expression in oocytes and
surrounding cumulus cells classified by BCB staining. (A) Genes
significantly co-expressed in BCB positive oocytes and surrounding
cumulus cells, comparatively to complex-oocyte complexes obtained from
BCB negative oocytes. (B) Genes significantly co-expressed in BCB
negative oocytes and surrounding cumulus cells, comparatively to
cumulus-oocyte complexes obtained from BCB positive oocytes. (C)
Independent clustering of oocytes and cumulus cells using 75 and 108
genes, respectively, co-expressing at r≥ 0.992. (D) Connectivity of the
genes expressed in oocytes and surrounding cumulus cells co-expressing
at r≥ 0.992. Only genes annotated with a symbol are depicted on panel D.

### sessionInfo

```
sessionInfo()
```

```
## R version 4.2.0 (2022-04-22)
## Platform: x86_64-pc-linux-gnu (64-bit)
## Running under: Ubuntu 20.04.4 LTS
## 
## Matrix products: default
## BLAS:   /usr/lib/x86_64-linux-gnu/blas/libblas.so.3.9.0
## LAPACK: /usr/lib/x86_64-linux-gnu/lapack/liblapack.so.3.9.0
## 
## locale:
##  [1] LC_CTYPE=en_US.UTF-8       LC_NUMERIC=C              
##  [3] LC_TIME=en_US.UTF-8        LC_COLLATE=en_US.UTF-8    
##  [5] LC_MONETARY=en_US.UTF-8    LC_MESSAGES=en_US.UTF-8   
##  [7] LC_PAPER=en_US.UTF-8       LC_NAME=C                 
##  [9] LC_ADDRESS=C               LC_TELEPHONE=C            
## [11] LC_MEASUREMENT=en_US.UTF-8 LC_IDENTIFICATION=C       
## 
## attached base packages:
##  [1] parallel  grid      stats4    stats     graphics  grDevices utils    
##  [8] datasets  methods   base     
## 
## other attached packages:
##  [1] ggdendro_0.1.23             networkD3_0.4              
##  [3] gridGraphics_0.5-1          ade4_1.7-19                
##  [5] vegan_2.6-2                 lattice_0.20-45            
##  [7] permute_0.9-7               dendextend_1.15.2          
##  [9] flashClust_1.01-2           data.table_1.14.2          
## [11] gtools_3.9.2.1              bigmemory_4.6.1            
## [13] reshape2_1.4.4              WGCNA_1.71                 
## [15] fastcluster_1.2.3           dynamicTreeCut_1.63-1      
## [17] cowplot_1.1.1               ggfortify_0.4.14           
## [19] gridExtra_2.3               ggrepel_0.9.1              
## [21] DGCA_1.0.2                  MEGENA_1.3.7               
## [23] igraph_1.3.1                doParallel_1.0.17          
## [25] iterators_1.0.14            foreach_1.5.2              
## [27] Rtsne_0.16                  ggpubr_0.4.0               
## [29] VennDiagram_1.7.3           futile.logger_1.4.3        
## [31] goseq_1.46.0                geneLenDataBase_1.30.0     
## [33] BiasedUrn_1.07              biomaRt_2.50.3             
## [35] DEsingle_1.14.0             DESeq2_1.34.0              
## [37] SummarizedExperiment_1.24.0 Biobase_2.54.0             
## [39] MatrixGenerics_1.6.0        matrixStats_0.62.0         
## [41] GenomicRanges_1.46.1        GenomeInfoDb_1.30.1        
## [43] IRanges_2.28.0              S4Vectors_0.32.4           
## [45] BiocGenerics_0.40.0         edgeR_3.36.0               
## [47] limma_3.50.3                lmtest_0.9-40              
## [49] zoo_1.8-10                  emmeans_1.7.4-1            
## [51] car_3.0-13                  carData_3.0-5              
## [53] aod_1.3.2                   multcomp_1.4-19            
## [55] TH.data_1.1-1               MASS_7.3-57                
## [57] survival_3.3-1              mvtnorm_1.1-3              
## [59] forcats_0.5.1               stringr_1.4.0              
## [61] dplyr_1.0.9                 purrr_0.3.4                
## [63] readr_2.1.2                 tidyr_1.2.0                
## [65] tibble_3.1.7                ggplot2_3.3.6              
## [67] tidyverse_1.3.1            
## 
## loaded via a namespace (and not attached):
##   [1] estimability_1.3         rappdirs_0.3.3           rtracklayer_1.54.0      
##   [4] prabclus_2.3-2           maxLik_1.5-2             coda_0.19-4             
##   [7] bit64_4.0.5              knitr_1.39               DelayedArray_0.20.0     
##  [10] rpart_4.1.16             KEGGREST_1.34.0          RCurl_1.98-1.6          
##  [13] generics_0.1.2           preprocessCore_1.56.0    GenomicFeatures_1.46.5  
##  [16] lambda.r_1.2.4           RSQLite_2.2.14           VGAM_1.1-6              
##  [19] bit_4.0.4                tzdb_0.3.0               xml2_1.3.3              
##  [22] lubridate_1.8.0          assertthat_0.2.1         viridis_0.6.2           
##  [25] xfun_0.31                hms_1.1.1                jquerylib_0.1.4         
##  [28] evaluate_0.15            DEoptimR_1.0-11          fansi_1.0.3             
##  [31] restfulr_0.0.13          progress_1.2.2           dbplyr_2.1.1            
##  [34] readxl_1.4.0             htmlwidgets_1.5.4        DBI_1.1.2               
##  [37] geneplotter_1.72.0       reshape_0.8.9            ellipsis_0.3.2          
##  [40] backports_1.4.1          annotate_1.72.0          vctrs_0.4.1             
##  [43] abind_1.4-5              cachem_1.0.6             withr_2.5.0             
##  [46] ggforce_0.3.3            robustbase_0.95-0        checkmate_2.1.0         
##  [49] bdsmatrix_1.3-4          GenomicAlignments_1.30.0 gamlss_5.4-3            
##  [52] prettyunits_1.1.1        mclust_5.4.10            cluster_2.1.3           
##  [55] crayon_1.5.1             genefilter_1.76.0        pkgconfig_2.0.3         
##  [58] labeling_0.4.2           tweenr_1.0.2             nlme_3.1-157            
##  [61] nnet_7.3-17              rlang_1.0.2              diptest_0.76-0          
##  [64] lifecycle_1.0.1          sandwich_3.0-1           bigmemory.sri_0.1.3     
##  [67] gamlss.data_6.0-2        filelock_1.0.2           BiocFileCache_2.2.1     
##  [70] modelr_0.1.8             cellranger_1.1.0         polyclip_1.10-0         
##  [73] Matrix_1.4-1             base64enc_0.1-3          reprex_2.0.1            
##  [76] png_0.1-7                viridisLite_0.4.0        rjson_0.2.21            
##  [79] bitops_1.0-7             Biostrings_2.62.0        blob_1.2.3              
##  [82] pdftools_3.2.0           multcompView_0.1-8       qpdf_1.1                
##  [85] jpeg_0.1-9               rstatix_0.7.0            ggsignif_0.6.3          
##  [88] scales_1.2.0             memoise_2.0.1            magrittr_2.0.3          
##  [91] plyr_1.8.7               zlibbioc_1.40.0          compiler_4.2.0          
##  [94] miscTools_0.6-26         BiocIO_1.4.0             bbmle_1.0.25            
##  [97] RColorBrewer_1.1-3       Rsamtools_2.10.0         cli_3.3.0               
## [100] XVector_0.34.0           htmlTable_2.4.0          Formula_1.2-4           
## [103] formatR_1.12             mgcv_1.8-40              tidyselect_1.1.2        
## [106] stringi_1.7.6            highr_0.9                yaml_2.3.5              
## [109] askpass_1.1              locfit_1.5-9.5           latticeExtra_0.6-29     
## [112] sass_0.4.1               tools_4.2.0              uuid_1.1-0              
## [115] rstudioapi_0.13          gamlss.dist_6.0-3        foreign_0.8-82          
## [118] farver_2.1.0             ggraph_2.0.5             digest_0.6.29           
## [121] fpc_2.2-9                Rcpp_1.0.8.3             broom_0.8.0             
## [124] pscl_1.5.5               httr_1.4.3               AnnotationDbi_1.56.2    
## [127] kernlab_0.9-30           colorspace_2.0-3         rvest_1.0.2             
## [130] XML_3.99-0.9             fs_1.5.2                 splines_4.2.0           
## [133] statmod_1.4.36           graphlayouts_0.8.0       flexmix_2.3-17          
## [136] xtable_1.8-4             jsonlite_1.8.0           futile.options_1.0.1    
## [139] tidygraph_1.2.1          modeltools_0.2-23        R6_2.5.1                
## [142] Hmisc_4.7-0              pillar_1.7.0             htmltools_0.5.2         
## [145] glue_1.6.2               fastmap_1.1.0            BiocParallel_1.28.3     
## [148] class_7.3-20             codetools_0.2-18         utf8_1.2.2              
## [151] bslib_0.3.1              numDeriv_2016.8-1.1      curl_4.3.2              
## [154] magick_2.7.3             GO.db_3.14.0             rmarkdown_2.14          
## [157] munsell_0.5.0            GenomeInfoDbData_1.2.7   impute_1.68.0           
## [160] haven_2.5.0              gtable_0.3.0
```
