# Supplementary material for: Tight gene co-expression in BCB positive cattle oocytes and their surrounding cumulus cells
Source: Reprod Biol Endocrinol. 2022 Aug 13;20:119. doi: 10.1186/s12958-022-00994-3 (PMC9375383; doi:10.1186/s12958-022-00994-3)
Supplement: Supplementary file 2 — Additional file 2: Supplementary figures. Additional file containing supporting figures [file 12958_2022_994_MOESM2_ESM.docx]

Supplemental figure 1. Mitochondrial DNA copy number quantified in single oocytes classified by brilliant cresyl blue staining.

Supplemental figure 2. Examples of null distribution created by randomizing the index of oocytes and breaking the link with the corresponding cumulus cells. Five randomizations are plotted, and their averages are: -0.02971, -0.002549, 0.08812, -0.04716, 0.06077.


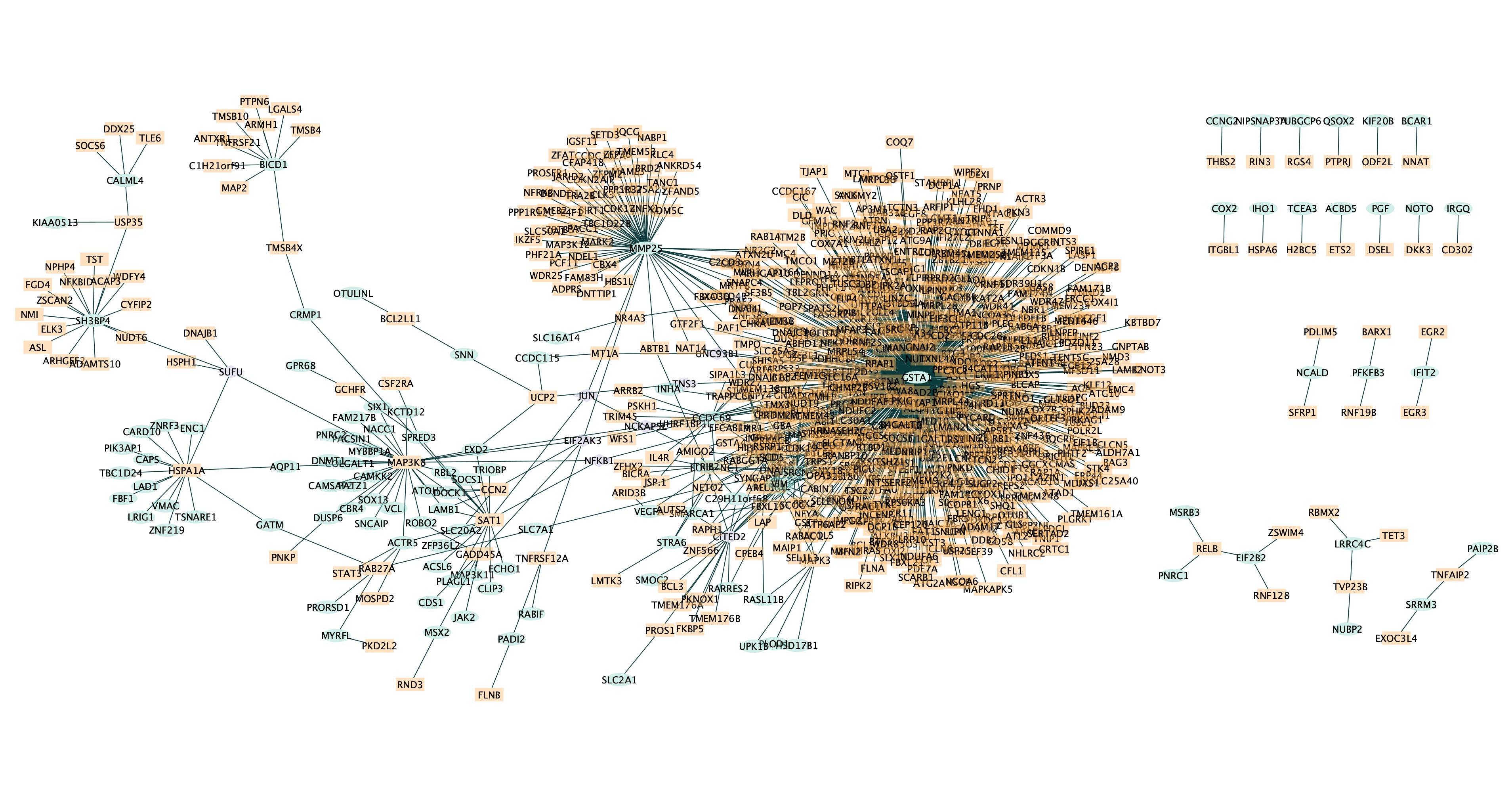


Supplemental figure 3. Network representation of the co-expression between oocytes and cumulus cells. Orange squares indicate genes expressed in cumulus cells, green ellipses indicate genes expressed in oocytes, purple ellipses indicate genes expressed in both cell types.

Supplemental figure 4. Null distribution of empirical false discovery rate calculated for different values of correlation. Blue and white circles indicate the values calculated for BCB positive and negative cumulus oocyte complexes, respectively. Horizontal line crosses y-axis at -Log_10_(0.0000005).
